# Wearable‐Based Monitoring of Autonomic and Gastrointestinal Function in Disorders of Gut‐Brain Interaction: A Systematic Review and Meta‐Analyses

**DOI:** 10.1111/nmo.70232

**Published:** 2026-01-19

**Authors:** Fleur Veldman, Michelle Bosman, Ali Rezaie, Sarvee Moosavi, Daniel Keszthelyi

**Affiliations:** ^1^ Department of Gastroenterology‐Hepatology, NUTRIM Research Institute of Nutrition and Translational Research in Metabolism Maastricht University Medical Center Maastricht the Netherlands; ^2^ GI Motility Program, Division of Gastroenterology, Department of Medicine Cedars‐Sinai Los Angeles California USA; ^3^ Neurogastroenterology & GI Motility, Department of Gastroenterology University of British Columbia Vancouver Canada

**Keywords:** autonomic dysfunction, disorders of gut‐brain interaction, gut‐brain axis, meta‐analysis, wearable electronic devices

## Abstract

**Background:**

Autonomic nervous system (ANS) activity is implicated in the pathogenesis of disorders of gut‐brain interaction (DGBI). Technological advances enable more accurate investigation of ANS function.

**Aim:**

This study aimed to evaluate the clinical utility of wearable devices in monitoring autonomic and gastrointestinal (GI) function in DGBI.

**Methods:**

A systematic search identified studies in adults with DGBI using wearables to assess heart rate variability (HRV), sleep, skin conductance, or gastric myoelectric activity as clinical readouts for ANS and GI function. The review provides an overview of available devices, while the meta‐analysis evaluates consistency in detecting differences between DGBI and healthy controls (HCs). Associations between autonomic function and GI symptom severity were explored. Methodological quality was assessed using the Cochrane risk of bias tool and ROBINS‐I. Meta‐analyses used random‐effects models with standardized mean differences (SMDs).

**Results:**

Thirty‐six studies (3 RCTs, 33 observational) involving 3986 DGBI patients were included (HRV: *n* = 16, sleep: *n* = 7, gastric myoelectric activity: *n* = 14, skin conductance: *n* = 0). Meta‐analyses showed lower Root Mean Square of Successive Differences (SMD = −0.503, SE 0.189, 95% CI [−0.873, −0.132]) and percentage of successive RR intervals differing by > 50 ms (SMD = −0.430, SE 0.176, 95% CI [−0.775, −0.085]), reflecting HRV alterations in DGBI versus HCs. No consistent differences were found for other metrics, except normal gastric slow waves (SMD = −0.722, SE 0.216, 95% CI [−1.146, −0.298]). Heterogeneous ANS‐symptom associations precluded definitive conclusions.

**Conclusions:**

Wearables show potential for detecting altered ANS and GI function in DGBI, particularly via HRV. Result variability highlights need for further research to confirm accuracy and clinical utility.

## Key Points

- Wearable devices hold potential for detecting altered autonomic nervous system and gastrointestinal function in disorders of gut‐brain interaction, particularly via HRV.
- Further standardized research is needed to confirm the accuracy and clinical utility of wearable devices.

## Graphical abstract

Wearables show promise for identifying autonomic and GI dysfunction in disorders of gut–brain interaction, particularly via HRV, though further standardized studies are required to validate their clinical use.

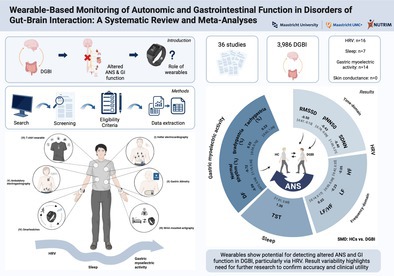

## 1 Introduction

Disorders of gut‐brain interaction (DGBI) are characterized by chronic gastrointestinal symptoms that lack detectable abnormalities on routine clinical evaluations [1, 2, 3, 4]. The pathophysiology is thought to be linked to dysregulation of the gut‐brain axis, which governs the coordinated interplay between gastrointestinal motility, sensory responses, and central nervous system (CNS) activity, via neural (i.e., autonomic [ANS] and the enteric nervous system [ENS]), immune, and endocrine mechanisms [5, 6, 7, 8].

The ANS plays a pivotal role in the bidirectional communication between the gut and the brain as well as in gastrointestinal (GI) function, providing key input to the ENS [9]. Given this role, disruptions in ANS activity may be central to the etiology and symptom generation in DGBI [5, 10, 11, 12, 13, 14]. As for measuring ANS function, the sham feeding pancreatic polypeptide (PP) test has previously been suggested as an objective indicator of vagus nerve integrity [15, 16]. However, studies in DGBI report inconsistent ANS dysfunction, with evidence of both heightened and diminished sympathetic [17, 18, 19, 20] and parasympathetic activity [10, 14, 21, 22, 23], possibly linked to symptom exacerbations [24, 25, 26, 27]. Methodological heterogeneity and small sample sizes complicate the interpretation of findings, precluding definitive conclusions regarding the role of ANS dysfunction in DGBI, and a comprehensive synthesis of ANS alterations in these conditions is currently lacking [5, 28]. Furthermore, it remains unclear whether ANS dysfunction, if at all present in DGBI, represents a causal mechanism or merely an epiphenomenon of other underlying pathophysiological processes.

Recent technological advancements enable continuous, non‐invasive ANS monitoring in real word‐settings through wearable devices, offering a promising alternative to conventional laboratory‐based measurements [9, 29]. These devices capture physiological metrics, such as heart rate variability (HRV), sleep patterns, and skin conductivity, which are considered markers of autonomic tone [10, 30, 31, 32, 33, 34, 35, 36]. Recent innovations within the wearable space also enable non‐invasive GI function assessment, which is largely influenced by ANS activity. Gastric myoelectric activity can now be measured using body surface gastric mapping (BSGM) [9, 37, 38, 39]. Together, these advances underscore the potential of real‐time monitoring of the autonomic brain–body connection through wearable technology, which may provide valuable insights into the role of the ANS in DGBI [9, 40]. Integrating these metrics with symptom‐tracking applications may enhance disease monitoring and understanding of autonomic and GI dysfunction in DGBI [29, 40].

Despite their growing accessibility, a comprehensive overview of wearables available for DGBI is lacking, with most studies focusing solely on HRV [10, 41, 42, 43]. However, a recent review on the use of wearables in inflammatory bowel disease suggested that these devices can detect autonomic alterations and may support timely clinical interventions [44]. Despite their promise, significant challenges remain (e.g., device‐related issues, regulatory and ethical considerations, patient concerns, data overload, and research limitations), and the interpretation of ANS function continues to be debated, highlighting the need for further research in this field [40, 44].

This study aims to provide a comprehensive overview of available wearable devices and systematically review their clinical utility in assessing autonomic and GI function in adults with DGBI. The meta‐analyses specifically evaluate whether wearables can distinguish DGBI patients from healthy controls (HCs).

## 2 Materials and Methods

This study adhered to the Preferred Reporting Items for Systematic Reviews and Meta‐Analyses (PRISMA) and the Cochrane Handbook for Systematic Reviews of Interventions [45]. It was registered in PROSPERO (CRD42022368536).

### 2.1 Eligibility Criteria

Inclusion criteria were: (I) any study design (II) involving adult participants (aged ≥ 18 years), (III) with a diagnosis of a DGBI, (IV) reporting the use of any wearable device, (V) for assessment of autonomic or GI function (i.e., HRV, sleep, gastric myoelectric activity, and skin conductance). There were no restrictions based on the study setting or other patient characteristics.

### 2.2 Search Strategy

A systematic search was conducted independently by two researchers (FV, MB) in PubMed, EMBASE, and the Cochrane Library (June 1964–November 2024). The results were compared, duplicates were removed, and the final set of search results was managed using Rayyan and EndNote21 software. See Supporting Information 1.1 for a detailed overview of the search.

### 2.3 Study Selection

Two researchers (FV, MB) independently screened all retrieved articles by title and abstract, followed by full‐text review of potentially eligible studies. Studies not meeting predefined criteria were excluded. Risk of bias was reviewed using the Cochrane risk of bias tool (RoB2) [46] for randomized controlled trials (RCTs) and ROBINS‐I [47] for observational studies. Disagreements were resolved through discussion or, if needed, by a third investigator (DK). See Supporting Information 1.2 and 1.3 for details.

### 2.4 Outcome Measures

The primary aim was to provide an overview of available wearable devices and evaluate their clinical utility in assessing autonomic and GI function in DGBI, including HRV, sleep, skin conductivity, and gastric myoelectric activity (see Table 1 and Supporting Information 1.4). For each autonomic and gastrointestinal metric, a qualitative synthesis was conducted to summarize available wearable devices. The meta‐analysis assessed measurement consistency, defined as the agreement in autonomic and GI measurements across studies, in DGBI and HCs.

**TABLE 1:** Autonomic and gastrointestinal parameters.

Parameter	Description	Autonomic activity
HRV		
Time‐domain parameters		
RMSSD (ms)	Root mean square of the successive differences between RR intervals	Parasympathetic activity
pNN50 (%)	Percentage of successive RR intervals differing by more than 50 ms	Parasympathetic activity
SDNN (ms)	Standard deviation of NN intervals (RR intervals after removing artifacts from the recorded ECG signal)	Both sympathetic and parasympathetic activity
SDNN index (ms)	Average of the standard deviations of all NN intervals for each 5‐min segment of a 24‐h recording	Both sympathetic and parasympathetic activity
SDANN (ms)	Standard deviation of the average NN intervals for each 5‐min segment of a 24‐h HRV recording	Both sympathetic and parasympathetic activity
SD‐5 min (ms)	Standard deviation of NN intervals within a single 5‐min segment	Both sympathetic and parasympathetic activity
Frequency‐domain parameters		
HF (ms^2^)	High frequency power	Parasympathetic activity
LF (ms^2^)	Low frequency power	Both sympathetic and parasympathetic activity
LF/HF ratio	Ratio of low‐frequency to high‐frequency power	Index of autonomic balance (sympathovagal balance)
TP (ms^2^)	Total power	Overall autonomic activity
Sleep		
TST (min)	Total sleep time, referring to the total amount of sleep achieved during one night	—
SE	Sleep efficiency, defined as the ratio of total sleep time to the total time spent in bed	—
SL (min)	Sleep latency, defined as the amount of time it takes to transition from full wakefulness to any stage of sleep	—
WASO (min)	Wake after sleep onset, representing the total number of minutes a person remains awake after initially falling asleep	—
Number of awakenings	The total number of times a person wakes up during the night	—
Gastric myoelectric activity		
DF (Hz)	Dominant frequency, defined as the frequency with the greatest power or amplitude in the signal, representing the most prominent oscillation in the signal spectrum	—
DP (dB)	Dominant power, defined as the amplitude or power at the dominant frequency, reflecting the intensity of the dominant oscillatory activity in the signal	—
Normal rhythm (%)	The percentage of time during which gastric slow waves fall within the normal frequency range (2.5–3.7 cycles per minute)	—
Bradygastria (%)	The percentage of time during which gastric slow waves occur within the bradygastria range (< 2.5 cycles per minute)	—
Tachygastria (%)	The percentage of time during which gastric slow waves are observed in the tachygastria range (> 3.7–10 cycles per minute)	—
Power ratio	The ratio of the dominant power of the gastric electrical activity within different frequency bands, indicating the relative power distribution of gastric rhythms	—
Sweat		
Skin conductivity (μS)	Measurement of the electrical conductance of the skin, which varies in response to moisture levels influenced by the activity of the sympathetic nervous system	Sympathetic activity

*Note:* The relationship between HRV metrics and autonomic activity is based on the studies by Ali et al. [10] and Gullett et al. [48]. For sleep and gastric myoelectric activity, no direct link to autonomic activity can be established. Skin conductivity is associated with sympathetic activity [49]. Bradygastria (%), percentage of slow waves in the bradygastria range (< 2.5 cycles per minute) [50]; DF, dominant frequency; DP, dominant power; HF, high frequency; LF, low frequency; LF/HF ratio, low frequency and high frequency ratio; Normal rhythm (%), percentage of gastric slow waves within the normal range (2.5–3.7 cycles per minute) [50]; pNN50, percentage of successive RR intervals differing by more than 50 ms; RMSSD, Root Mean Square of the successive differences between RR intervals; SDANN, Standard Deviation of the Averages of NN intervals; SD‐5 min, standard deviation over 5 min; SDNN, standard deviation of normal‐to‐normal intervals; SDNN index, average of the standard deviations of all the NN intervals of each 5 min segment; SE, sleep efficiency; SL, sleep latency; Tachygastria (%), percentage of slow waves in the tachygastria range (> 3.7–10 cycles per minute) [50]; TP, total power; TST, total sleep time; WASO, wake after sleep onset.

The secondary aim was to qualitatively examine associations between autonomic function and GI symptom severity in DGBI.

### 2.5 Statistical Analyses

Data were analyzed using R (version 4.4.2, *metaphor* package). Means with standard deviations were reported for continuous variables and frequencies (%) for categorical variables. Meta‐analyses evaluated measurement consistency across studies comparing DGBI to HCs, requiring ≥ 5 studies per metric [51]. Descriptive analyses were conducted for metrics with fewer studies. Data were pooled using a random‐effects model with standardized mean differences (SMD) to account for between‐study heterogeneity. The SMDs indicate the contrast between DGBI and HCs, with negative SMDs reflecting lower values in DGBI. Given the exploratory nature of the analyses, emphasis was placed on pooled effect sizes with 95% confidence intervals (CI), while unadjusted *p*‐values were provided only in tables for completeness. Hence, results should be interpreted with caution and considered hypothesis‐generating, requiring replication. Forest plots were created to visualize study‐level results and overall pooled effects. Funnel plots were generated to examine potential publication bias. Heterogeneity was assessed using *I* ^2^ (low: < 25%, moderate: 25%–50%, high: > 50%) and *Q*‐statistics (*p* ≤ 0.10) [52]. Sensitivity analyses (leave‐one‐out) were used to identify influential studies. Subgroup or meta‐regression analyses were not conducted due to the limited number of studies per variable. See Supporting Information 1.5 for full details per metric.

## 3 Results

### 3.1 Study Selection

**FIGURE 1:**
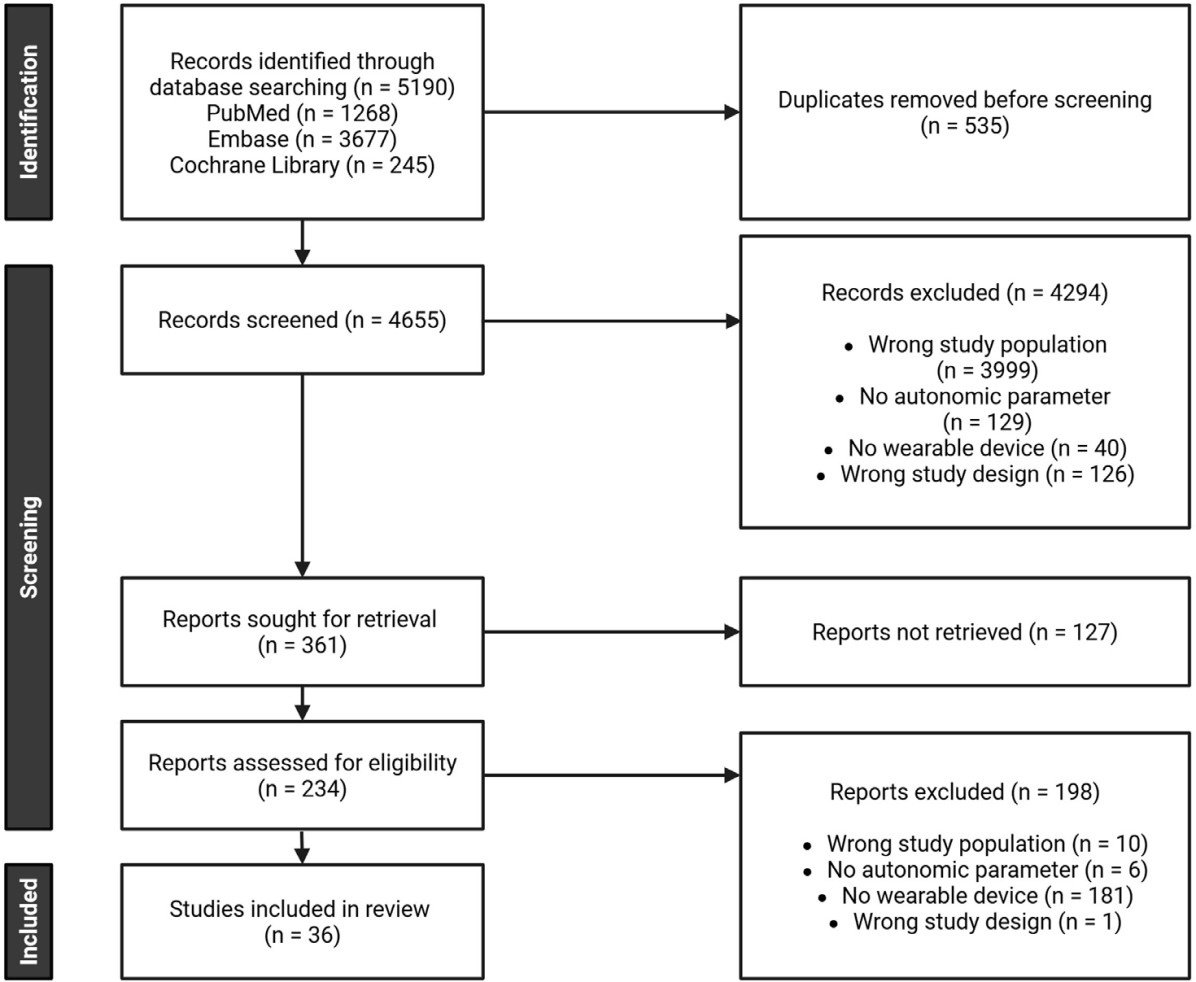
Study selection. PRISMA flow chart illustrating the selection process. PRISMA, Preferred Reporting Items for Systematic Reviews and Meta‐Analysis.

The initial search yielded 5190 records (PubMed: 1268, Embase: 3677, Cochrane Library: 245). After removing duplicates, 4655 titles and abstracts were screened, and 234 full‐text articles were assessed for eligibility. In total, 36 studies were included (Figure 1).

### 3.2 Study Characteristics

**FIGURE 2:**
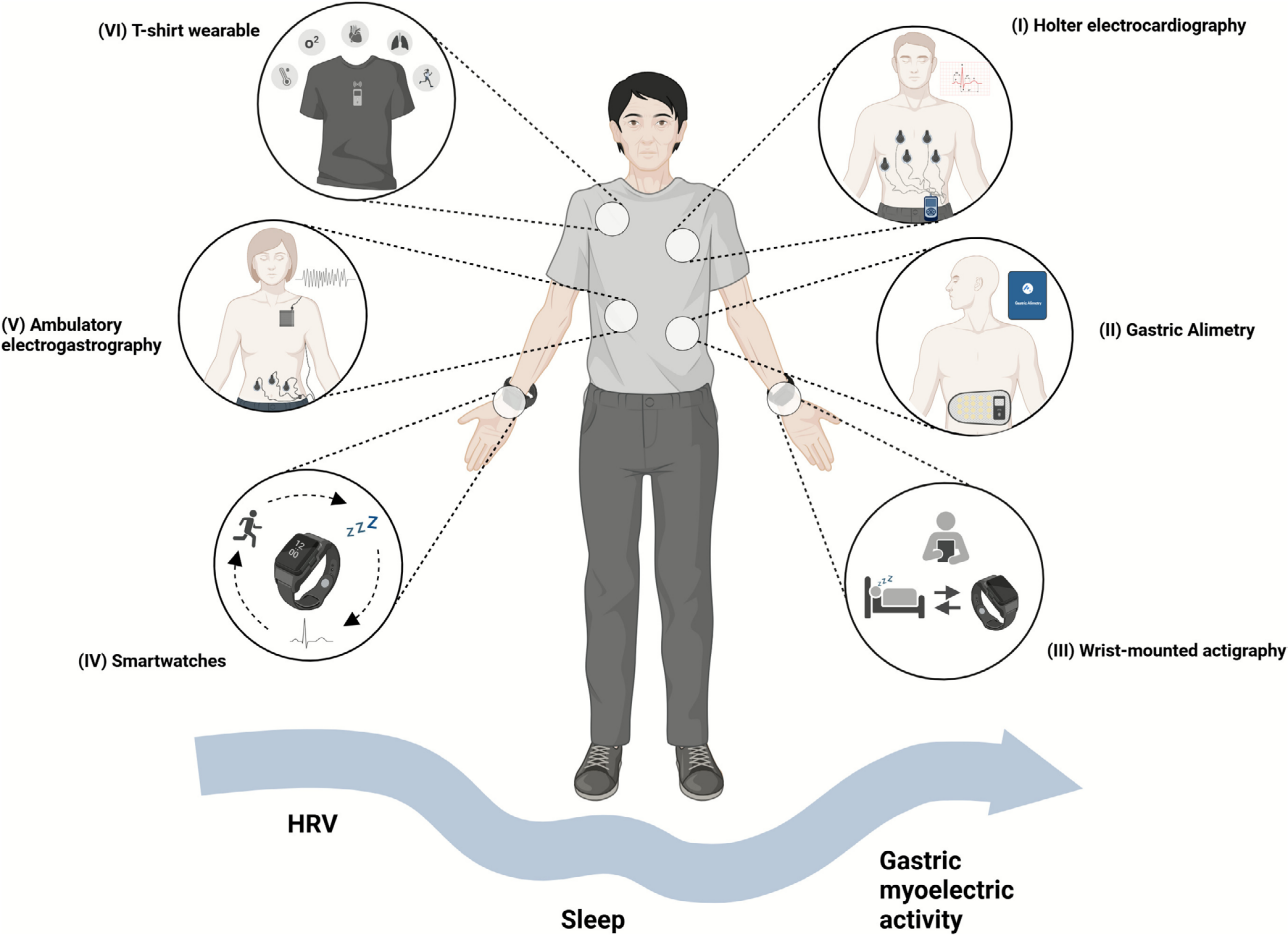
Overview of wearable devices for assessing autonomic and gastrointestinal function. (I) Holter electrocardiography for HRV monitoring, (II) gastric alimetry for gastric myoelectric activity, (III) wrist‐mounted actigraphy for sleep monitoring, (IV) smartwatches, including Fitbit, Active Tracer, and Sleepthing for HRV or sleep monitoring, (V) Ambulatory electrogastrography, including Digitrapper and wireless motility patches, for gastric myoelectric activity, (VI) a T‐shirt wearable for HRV monitoring. This figure was made with BioRender. HRV, heart rate variability.

**FIGURE 3:**
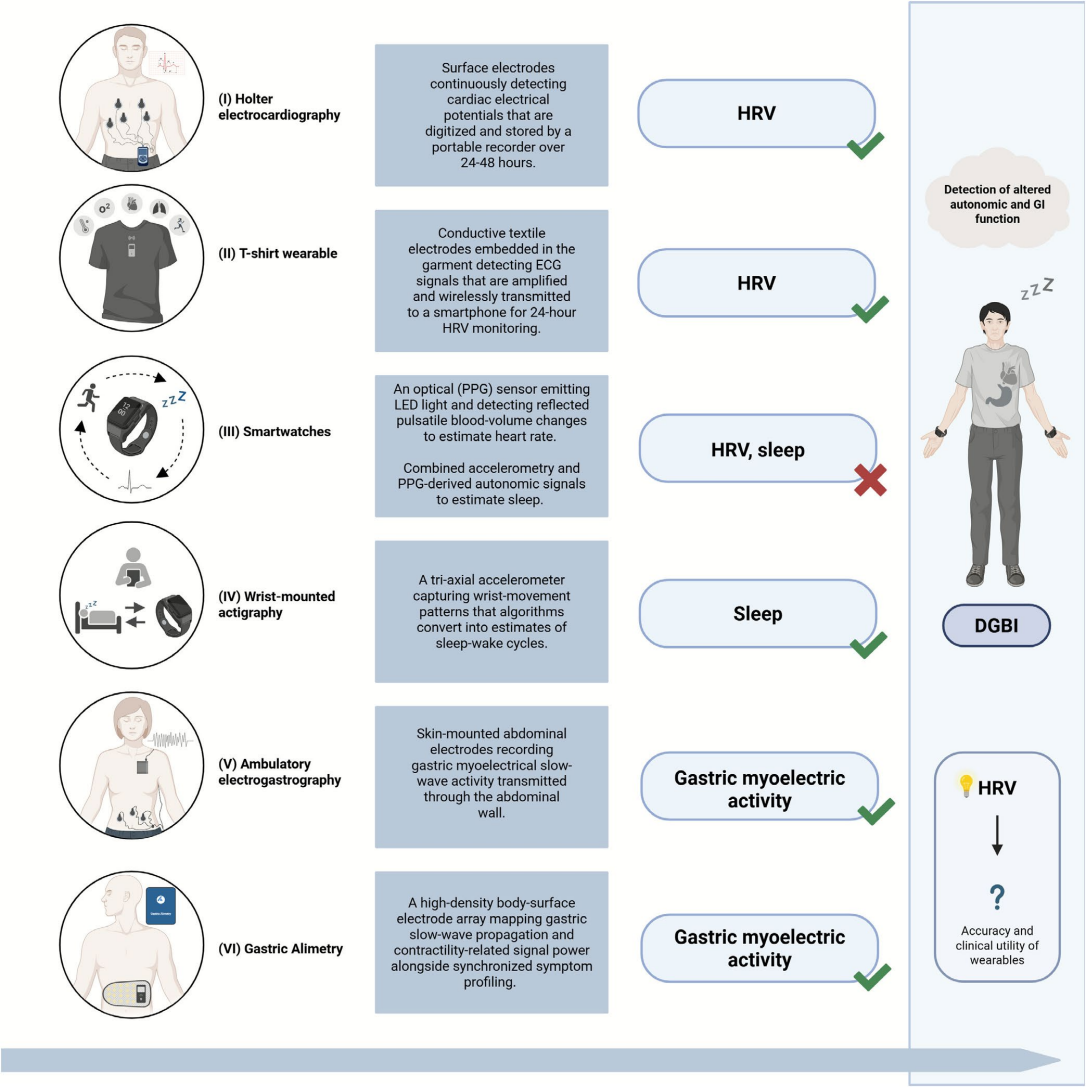
Technical and practical overview of wearable devices. Summary of wearable devices, their underlying technical aspects, and their validation status for the measured parameters (check mark = validated; cross mark = not validated). Validation status reflects device performance in general populations and does not imply validation specifically in DGBI. Wearable‐based detection of autonomic and gastrointestinal alterations in DGBI shows potential, particularly through HRV‐based assessment, although device accuracy and clinical utility remain to be confirmed in future studies. DGBI, disorder of gut‐brain interaction; ECG, electrocardiography; HRV, heart rate variability; PPG, pulse plethysmography.

Thirty‐six studies (3 RCTs, 33 observational) published between 1997 and 2024 assessed autonomic and GI function in adults with DGBI (*n* = 3986) using wearable devices, including Holter electrocardiography (*n* = 13), wearable T‐shirt (*n* = 1), smartwatches (*Active Tracer* [*n* = 1], *Fitbit* [*n* = 1], *Sleepthing* [*n* = 1]), wrist‐mounted actigraphy (*n* = 5), ambulatory electrogastrography (*n* = 13, 4 BSGM, 1 wireless motility patches) (Figures 2 and 3). These measured HRV (*n* = 16), sleep (*n* = 7), or gastric myoelectric activity (*n* = 14). No included studies investigated skin conductance. Among the 24 studies comparing DGBI and HCs, 20 were included in meta‐analyses. Four were excluded due to missing data. See Supporting Information 2.1–2.3 for details and bias assessment.

### 3.3 Heart Rate Variability

Sixteen studies investigated HRV in DGBI (*n* = 2645), including IBS (*n* = 928, 11 studies), FD (*n* = 177, 4 studies), and functional constipation (*n* = 1540, 1 study) (Table 2). Devices included Holter electrocardiography (13 studies), a T‐shirt device (1), Active Tracer (1), and Fitbit (1). Time‐domain (9 studies) and frequency‐domain (13 studies) HRV parameters were reported; see Table 1 for specification.

**TABLE 2:** Overview of study results by autonomic and gastrointestinal parameters.

Study	Study design	Study objective	Total study population (*n*=)	DGBI	Wearable	Study groups	Outcome parameters	Results
Studies assessing HRV as an outcome measure								
Akhan et al. (2023)^a^	Non‐RCT	To examine HRV in IBS and compare time‐domain HRV metrics between IBS‐D and IBS‐C subgroups	100	IBS	Holter electrocardiography	1. IBS‐D 2. IBS‐C 3. HC	Minimum HR, maximum HR, mean HR, HRD width, RMSSD, RRSD, pNN50, SDNN index	The total heartbeat, minHR, RMMSD, pNN50, and SDNN index were significantly lower in IBS patients than in HCs (all *p* < 0.05). No significant differences were observed among the IBS subtypes IBS‐C and IBS‐D (*p* > 0.05)
Cain et al. (2006)^a^, ^d^	Non‐RCT	To examine HRV in women with IBS to assess its link to gut pain and bowel patterne	215	IBS	Holter electrocardiography	1. IBS 2. HC	HF, LF, LF/HF ratio	Women with IBS‐C and severe gut pain had lower HF power and a higher LF/HF ratio compared to women with IBS‐D and HCs (*p* < 0.05). No significant differences were observed in women with mild or moderate gut pain
Chen et al. (2024)^d^	Non‐RCT	To examine the relationship between HRV and daily nausea and heartburn symptoms in younger and older women with IBS	89	IBS	Holter electrocardiography	1. IBS ≤ 46 years 2. IBS > 46 years	SDNN, SD‐5 min, RMSSD, pNN50	Older women with IBS exhibited reduced vagal activity (RMSSD and pNN50, both *p* < 0.001) compared to their younger counterparts. In older IBS patients (> 46 years), nausea inversely correlated with RMSSD (*p* = 0.041), but no associations were found in younger patients
Durakoğlugil, et al. (2014)^a^	Non‐RCT	To examine the effect of IBS on HRV parameters	60	IBS	Holter electrocardiography	1. IBS 2. HC	SDNN, SDANN, RMSSD, TP, LF, HF, VLF, LF/HF ratio, HR, SDNN index	SDNN index (*p* = 0.010), RMSSD (*p* = 0.002), LF (*p* = 0.002), and HF (*p* = 0.006) were significantly lower in IBS patients compared to controls. The LF/HF ratio was not significantly different between the two groups
Heitkemper et al. (1998)^a^	Non‐RCT	To assess ANS function in women with and without IBS	40	IBS	Holter electrocardiography	1. IBS 2. HC	LF, MF, HF, LF/HF ratio, SQLFHF	Women with IBS demonstrated significant lower values of the natural log of HF (*p* = 0.02) and the square root of the LF/HF ratio (*p* = 0.008) compared to HCs. The natural log of LF was not significantly different (*p* = 0.30)
Heitkemper, et al. (2001)^a^, ^d^	Non‐RCT	To examine autonomic function in patients with IBS	152	IBS	Holter electrocardiography	1. IBS 2. HC	LF, TP, SD‐5 min, SDANN, SD‐24 h, HF, RMSSD, pNN50, LF/HF ratio	24‐h measurements showed no significant HRV differences between IBS patients and HCs (*p* > 0.05). RMSSD (*p* = 0.017) and pNN50 (*p* = 0.005) were lower in IBS‐C patients with severe symptoms, while the square root of LF/HF ratio (*p* = 0.046) ratio was higher compared to IBS‐D
Jarrett et al. (2003)^a^	Non‐RCT	To examine autonomic function differences between women with IBS and a history of anxiety and depression and those without	209	IBS	Holter electrocardiography	1. IBS 2. HC	lnHF, lnLF, sqLF/HF ratio, RMSSD, pNN50, SD‐5 min	A history of anxiety and depressive disorders is associated with reduced parasympathetic (pNN50, RMSSD, lnHF) and general ANS activity (SD‐5 min, lnLF) in women with IBS compared to those without such history. No significant differences were observed in HRV metrics between controls with and without such history
Jarrett, Cain, et al. (2016)^d^	RCT	To identify biomarkers that predict which IBS subgroups will benefit most or least from a CSM intervention	85	IBS	Holter electrocardiography	1. CSM 2. Usual care	HF, LF/HF.	IBS patients with lower HF power (*p* = 0.015) and a higher LF/HF ratio (*p* < 0.001) showed a reduced response to a self‐management intervention aimed at reducing abdominal pain
Jarrett, Han, et al. (2016)^a^, ^d^	Non‐RCT	To assess differences in night‐time HRV between IBS subgroups and controls	91	IBS	Holter electrocardiography	1. IBS 2. HC	HF, LF, TP, LF/HF ratio	HRV indices (HF, LF, LF/HF, TP) did not significantly differ between IBS patients and controls, nor between IBS subgroups (all *p* > 0.05). A positive association was found between TP and LF with abdominal pain (both *p* < 0.05), while no significant relationship was observed with HF or the LF/HF ratio
Polster et al. (2018)^a^, ^d^	Non‐RCT	To identify IBS subgroups with distinct ANS characteristics compared to healthy controls	197	IBS	Holter electrocardiography	1. IBS 2. HC	SDNN, RMSSD, pNN50, LFn.u., HFn.u., LF/HFn.u. ratio, SDNN index	IBS patients showed significant differences from HCs in HRV parameters (SDNN, RMSSD, pNN50, SDDN index, HF, LF, LF/HF) during the daytime and in the standing position (*p* < 0.05). IBS‐D patients exhibited an abnormal HRV profile, with a significant correlation between HRV and diarrhea severity (*p* = 0.02)
Nakata et al. (2022)^a^, ^d^	Non‐RCT	To assess the relationship between ANS activity and IBS symptoms, using real‐time recording	20	IBS	T‐shirt wearable device	1. IBS 2. HC	LF/HF ratio, HF, TP, CVRR, IBI, SDNN	No significant differences were observed in the mean LF/HF, HF, and TP values between IBS patients and HCs (*p* > 0.05). The sum of the LF/HF ratio before defecation positively correlated with the intensity of abdominal pain (*p* = 0.01), diarrhea (*p* < 0.01), and constipation (*p* = 0.01) in IBS patients
Dal et al. (2014)^a^	Non‐RCT	To evaluate HRV differences between FD subgroups (i.e., PDS and EPS) and healthy controls	126	FD	Holter electrocardiography	1. FD 2. HC	RMSSD, pNN50, SDNN, LF/HF ratio	FD patients had significantly lower 24‐h RMSSD (*p* = 0.047) and pNN50 (*p* = 0.017) values compared to HCs. No significant differences were found in other 24‐h HRV parameters (SDNN, LF/HF) (*p* > 0.05)
Ochi et al. (2013)	Non‐RCT	To evaluate if classifying FD patients into two subgroups (i.e., PDS and EPS) based on clinical complaints accurately reflects their pathophysiology	45	FD	Holter electrocardiography	1. PDS 2. EPS	LF, HF, LF/HF ratio	73.5% of FD patients showed reduced HF power and 32.4% showed elevated LF/HF ratios compared to controls. No differences in ANS function were observed between the PDS and EPS subgroups
Lorena et al. (2002)^a^	Non‐RCT	To assess ANS function in patients with FD	35	FD	Holter electrocardiography	1. FD 2. HC	LF, HF, LF/HF ratio, RMSSD, SDNN, pNN50.	FD patients had significantly lower RMSSD (*p* = 0.02) and HF power (P0.04) compared to HCs. Other indices showed no significant differences (*p* > 0.05)
Tominaga et al. (2016)^d^	Non‐RCT	To elucidate the role of the ANS in FD patients	45	FD	Active Tracer	1. PDS 2. EPS	HF, LF, LF/HF ratio	Low HF power (86.7%, 97.8%, 66.7%) and higher LF/HF ratios (51.1%, 73.3%, 26.6%) were observed in 24‐h, daytime, and nighttime measures. GI Symptom Rating Scale (GSRS) scores did not differ between groups with abnormal and normal HRV
Shapiro et al. (2021)^d^	Non‐RCT	To understand day‐to‐day variations in symptoms and medication management in people with functional constipation	1540	Functional constipation	Fitbit	1. Daily population 2. Behavioral population 3. Monthly population	HR, HRD width	Active heart rate was lower on irregular and constipated bowel movement days compared to regular bowel movement days in patients with functional constipation (both *p* < 0.05)
Studies assessing sleep as an outcome measure								
Shapiro et al. (2021)^d^	Non‐RCT	To understand day‐to‐day variations in symptoms and medication management in people with functional constipation	1540	Functional constipation	Fitbit	1. Daily population 2. Behavioral population 3. Monthly population	TST, SE, TIB.	Fitbit data showed a small reduction in TST and SE on irregular bowel movement days (−2.4 min, 95% CI [−4.3, −0.4] and −0.10, 95% CI [−0.19, −0.01], resp.) and a larger reduction in TST on constipated days (−4.0, 95% CI [−6.5, −1.4]), compared to regular bowel movement days
Buchanan et al. (2014)^d^	Non‐RCT	To examine whether actigraphy and self‐reported sleep predict next‐day symptoms in women with IBS and vice versa	24	IBS	Wrist‐mounted actigraphy	1. IBS	SE, TST, SL, WASO	Sleep efficiency did not predict next‐day GI symptom severity or abdominal pain in IBS (across‐subjects: *p* = 0.742 and *p* = 0.488; within‐subjects: *p* = 0.373 and *p* = 0.121). However, GI symptoms were significantly associated with improved actigraphic sleep efficiency (*p* = 0.012). A significant but weak correlation was found between self‐rated sleep quality and actigraphic sleep efficiency (*r* = 0.227, *p* = 0.001)
Patel et al. (2016)^a^, ^d^	Non‐RCT	To assess the impact of sleep on next‐day IBS symptoms, IBS‐QOL, and non‐GI pain	50	IBS	Wrist‐mounted actigraphy	1. IBS 2. HC	TST, mean sleep duration, mean time to fall asleep, mean number of awakenings per night	IBS patients demonstrated more waking episodes during sleep than HCs (*p* < 0.001). Increased waking episodes predicted worse abdominal pain (*p* < 0.01) and GI distress (*p* < 0.001), but not bowel pattern or accessory IBS symptoms (*p* > 0.03)
Rotem, et al. (2003)^a^, ^d^	Non‐RCT	To assess sleep function and quality in IBS patients	38	IBS	Wrist‐mounted actigraphy	1. IBS 2. HC	Sleep period, TST, SL, SE, WASO, SFI, AwI	Actigraphy showed greater sleep fragmentation in IBS patients compared to HCs (*p* < 0.01). Other sleep metrics were not different (*p* > 0.05). Severe IBS was linked to lower sleep efficiency (*p* = 0.02) and more WASO episodes (*p* < 0.04)
Topan et al. (2024) %	Non‐RCT	To examine sleep quality and GI symptoms using actigraphy and ESM	80	IBS	Wrist‐mounted actigraphy	1. IBS	SE, SL, early morning awakening, WASO	Sleep efficiency was low (86%, 95% CI [85%–87%]), and sleep onset latency was high (29.9 min, IQR: 10.5–60.7), while WASO was within normal limits (22 min, IQR: 11.3–40.0) in IBS. No significant associations between objective sleep measures and GI symptoms in IBS were found (*p* > 0.05)
Du et al. (2023) %	RCT	To examine the effect of sleep improvement on epigastric pain in FD	107	FD	Sleepthing	1. FD	TST, SE, SL, SD, WASO, NREM	TST, SE, SL, WASO, and NREM showed significant differences post‐treatment in the sleep medication group compared to the vitamin B group (*p* < 0.05). Improving sleep in FD patients with sleep medication alleviated FD‐associated epigastric pain
Ono et al. (2008)^a^	Non‐RCT	To investigate the relationship between functional constipation and sleep health using objective measures	20	Functional constipation	Wrist‐mounted actigraphy	1. Functional constipation 2. HC	TST, WASO during the first half of the sleep period, WASO during the second half of the sleep period, average activity during the sleep period, average activity during the first half of the sleep period, average activity during the second half of the sleep period, sleep time during the awake period from sleep offset time to 12:00 and sleep time during the awake period from 12:00 to sleep onset time	WASO and average activity during sleep were significantly higher in patients with functional constipation compared to HCs (*p* < 0.01). No significant differences were observed for sleep onset time, sleep offset time, and TST (*p* > 0.05)
Studies assessing gastric myoelectric activity as outcome measure								
Orr et al. (1997)^b^	Non‐RCT	To confirm REM sleep alterations in IBS patients and assess the relationship between sleep and gastric function using EGG	20	IBS	Ambulatory electrogastrogram	1. IBS 2. HC	Dominant frequency.	No significant changes in the amplitude of the dominant EGG frequency from waking to non‐REM sleep, or from non‐REM to REM sleep, were observed in the IBS group compared to controls (*p* > 0.05)
Hocke et al. (2001)^a^	Non‐RCT	To identify parameters that differentiate between diseases using 24‐h cutaneous EGG	54	FD	Ambulatory electrogastrogram	1. FD 2. Systemic sclerosis 3. IBS 4. Delayed gastric emptying 5. HC	Percentages of dominant frequency in the normogastric range, in bradygastria, and in tachygastria, dominant power	Patients with FD had significantly more bradygastria both peri prandially and after 24 h compared to HCs (*p* < 0.001). IBS patients showed a reduction in tachygastria in the 24‐h measurements, as well as pre‐ (*p* < 0.01) and postprandially (*p* < 0.05). FD patients had higher dominant power than IBS patients (*p* < 0.05)
Riezzo et al. (2001)^a^, ^d^	Non‐RCT	To explore gastric electrical activity, gastric emptying, and GI hormones in dyspeptic patients and their relation to *H. pylori* status	51	FD	Ambulatory electrogastrogram	1. FD 2. HC	Emptying time, half emptying time, AUC of the emptying time, dominant frequency, instability coefficient of dominant frequency, dominant power, instability coefficient of dominant power, normal slow wave, bradygastria, tachygastria	The area under the curve (AUC) for the normal slow wave percentage was significantly lower in dyspeptic patients compared to controls (*p* = 0.003), while the percentage of tachygastria was significantly higher (*p* < 0.001). No significant differences were observed in the dominant frequency or percentage of bradygastria between groups (*p* > 0.05). No correlation was found between EGG metrics and dyspepsia scores in FD patients
Parkman et al. (1997)^d^	Non‐RCT	To determine the frequency of EGG and gastric emptying abnormalities in unexplained dyspepsia and correlate them with symptom severity	72	FD	Ambulatory electrogastrogram	1. FD	Dominant slow‐wave frequency in both the fasting and postprandial periods, percentage of time the dominant frequency was in bradygastria, normal rhythm, tachygastria, and duodenal‐respiratory range for both the fasting and postprandial periods, power ratio	EGG abnormalities were more frequent in dyspeptic patients with delayed gastric emptying and greater symptom severity, compared to those with normal gastric emptying (50% vs. 22%, *p* < 0.025). FD patients with abnormal EGG results exhibited greater symptom severity, with a mean symptom score of 9.4 ± 0.8, compared to 7.4 ± 0.5 in those with normal EGGs. Patients with abnormal EGGs had significantly higher scores for upper abdominal discomfort (*p* = 0.036) and anorexia (*p* = 0.048), while other symptoms did not show significant differences
Pfaffenbach et al. (1997)^c^, ^d^	Non‐RCT	To investigate antral myoelectric activity and gastric emptying in FD patients	45	FD	Ambulatory electrogastrogram	1. FD 2. HC	Dominant frequency, DF in the normal range, bradygastria, tachygastria, dominant frequency instability coefficient, and postprandial to fasting power ratio (PR)	Patients with FD demonstrated a pre‐prandial increase in tachygastria compared to controls (*p* < 0.001). Other EGG values did not significantly differ between groups. Patients with delayed gastric emptying showed significantly more pre‐ and postprandial tachygastrias than those with normal gastric emptying (*p* < 0.05)
Pfaffenbach et al. (1998)^a^, ^d^	Non‐RCT	To investigate the relationship between gastric electrical activity and motility in dyspeptic patients with gastrointestinal or extraintestinal disease	175	FD	Ambulatory electrogastrogram	1. FD 2. TIIDM 3. Hyperthyroidism 4. Progressive chronic scleroderma 5. Chronic alcoholism 6. Gastric ulcer or cancer 7. HC	Dominant electrical frequency, percentage of DF in the normal frequency range, bradygastria, tachygastria, dominant frequency instability coefficient, power ratio	Patients with delayed gastric emptying exhibited significantly more tachygastrias than those with normal gastric emptying (*p* < 0.05). The dyspepsia score did not correlate with EGG metrics
Miyaji et al. (1999)^a^	Non‐RCT	To evaluate the effects of *H. pylori* eradication on GI motility and symptoms in non‐ulcer dyspepsia patients	56	FD	Ambulatory electrogastrogram	1. FD 2. HC	Gastric emptying pattern, prevalence of normal EGGs pattern, pre‐prandial normal range EGGs, postprandial normal range EGGs	The prevalence of normal EGG patterns was significantly lower in both *H. pylori* positive (*p* < 0.05) and negative FD patients (*p* < 0.001). Abnormal antral myoelectric activity was observed in both *H. pylori* positive and negative FD patients
Zhang et al. (2015)	RCT	To evaluate the clinical short‐ and long‐term effects of combining Dalitong Granule and electroacupuncture in treating FD	635	FD	Ambulatory electrogastrogram	1. Combined DG and EA group 2. EA group 3. DG group 4. Control group	The percentage of normal gastric, bradygastria, and tachygastria waves, the dominant frequency, the dominant power, coefficient of dominant frequency instability, dominant coefficient power instability, fed: fasted amplitude ratio	The combined treatment group showed significant improvements in quality of life, plasma motilin levels, electrogastric frequencies, and gastric emptying at T2, with continued benefits at T3. The EA group showed better outcomes than the DG and control groups, although their effects were less sustained by T3. Both the DG and control groups showed a decline in these indices at T3
Zhao et al. (2010)^a^, ^d^	Non‐RCT	To explore clinical patterns, predisposing factors, psychosocial aspects, and potential pathogenesis in functional vomiting patients	29	Functional vomiting	Ambulatory electrogastrogram	1. Functional vomiting 2. HC	Dominant frequency, dominant power, % normal rhythm, % bradygastria, % tachygastria	Patients with functional vomiting showed a significant lower percentage of normal gastric slow waves and postprandial DF, along with increased DP, and abnormal PR compared to HCs. The severity of vomiting correlated with a reduced percentage of post‐prandial normal gastric slow waves (*p* = 0.020), and nausea correlated with the power ratio (*p* = 0.041). No significant associations were found for other upper GI symptoms
Gharibans et al. (2022)^a^, ^d^	Non‐RCT	To present a novel medical device and clinical procedure of non‐invasive BSGM and its first application in CNVS patients	86	CNVS	Ambulatory electrogastrogram (Gastric Alimetry)	1. CNVS 2. HC	Mean amplitude, dominant frequency, average amplitude, fed‐to‐fasting power ratio, spatial frequency stability, average spatial covariance	CNVS patients exhibited reduced amplitudes (*p* < 0.001), impaired fed‐fasting power‐ratios (*p* = 0.02), and disorganized slow waves (*p* < 0.001) compared to controls. Abnormal BSGM metrics were correlated with symptom severity, including nausea, pain, excessive fullness, early satiety, bloating, and heartburn (all *p* < 0.05)
Schamberg et al. (2023)^a^, ^d^	Non‐RCT	To compare GA BSGM and EGG tests in a standardized manner to quantify performance differences	178	CNVS	Ambulatory electrogastrogram (Gastric Alimetry)	1. CNVS 2. T1DM 3. HC	BMI‐adjusted amplitude, dominant frequency, GA rhythm index, fed: fasted amplitude ratio	Both BSGM and EGG detected rhythm instability in CNVS patients. While EGG detected group‐level differences, it showed poor accuracy for patient‐level classification and lacked symptom correlations. BSGM demonstrated significant improvements across all domains, with strong correlations between symptoms and BSGM metrics (all *p* < 0.05)
Lacy et al. (2024)^d^	Non‐RCT	To investigate the safety and utility of the WPS in patients with chronic gastroduodenal symptoms	22	Chronic gastroduodenal symptoms	Wireless motility patches	1. Normal GE 2. Delayed GE	Gastric, intestinal, and colonic activity within the 1–28 cpm ranges	FD patients with delayed gastric emptying exhibited unique gastrointestinal myoelectrical activity patterns using wireless motility patches. Patients with higher bloating scores (cut‐off: 600 on the Mayo Bloating Questionnaire) had significantly lower total gastric (*p* = 0.011) and colonic activity (*p* = 0.054) compared to those with lower bloating scores
Law et al. (2024)^b^	Non‐RCT	To investigate the short‐ and long‐term reproducibility of BSGM in controls and patients with chronic gastroduodenal symptoms	28	Chronic gastroduodenal symptoms	Ambulatory electrogastrogram (Gastric Alimetry)	1. Chronic gastroduodenal symptoms 2. HC	Dominant frequency, BMI‐adjusted amplitude, GA rhythm index, fed: fasted amplitude ratio	BSGM metrics demonstrated high reproducibility, with no significant differences between short‐term and long‐term tests in patients with chronic gastroduodenal symptoms (all *p* > 0.180)
Wang et al. (2023)^d^	Non‐RCT	To assess patient‐specific phenotyping using GA and gastric emptying tests	75	Chronic gastroduodenal symptoms	Ambulatory electrogastrogram (Gastric Alimetry)	1. Chronic gastroduodenal symptoms	GA rhythm index, dominant frequency, BMI‐adjusted amplitude, fed: fasted amplitude ratio	Delayed gastric emptying in patients with chronic gastroduodenal symptoms did not predict specific Gastric Alimetry phenotypes, and no significant differences in symptoms were observed based on gastric emptying status

*Note:* ANS, autonomic nervous system; AUC, area under the curve; AwI, awakening index; BMI, body mass index; Bradygastria (%), percentage of slow waves in the bradygastria range (< 2.5 cycles per minute) [50]; CNVS, Chronic Nausea and Vomiting Syndrome; CSM, Comprehensive Self‐Management intervention; CVRR, Coefficient of Variation of RR intervals; DF, dominant frequency; DFIC, Dominant frequency instability coefficient; DG, Dalitong Granule; DGBI, Disorder or Gut‐Brain Interaction; EA, Electroacupuncture; EGG, electrogastrography; EPS, Epigastric Pain Syndrome; FD, Functional Dyspepsia; GA‐R, Gastric Alimetry Rhythm; GE, Gastric Emptying; GSRS, GI symptom rating scale; HC, Healthy Controls; HF, High Frequency; H. pylori, *Helicobacter pylori* ; HR, Heart Rate; HRD, Heart Rate Distribution; HRV, heart rate variability; IBI, Inter‐Beat Interval; IBS, Irritable Bowel Syndrome; IBS‐C, IBS Constipation; IBS‐D, IBS Diarrhea; LF, Low Frequency; LF/HF Ratio, Low Frequency and High Frequency Ratio; ln, natural logarithm; MF, Middle Frequency; Normal rhythm (%), percentage of gastric slow waves within the normal range (2.5–3.7 cycles per minute) [50]; NREM, non‐rapid eye movement sleep; n.u., normalized units; PDS, Postprandial Distress Syndrome; pNN50, percentage of successive RR intervals differing by more than 50 ms; PR, power ratio; RCT, randomized controlled trial; REM, rapid eye movement sleep; RMSSD, Root Mean Square of Successive Differences between RR intervals; RRSD, Standard Deviations of the RR Interval; SD, sleep disruption; SDANN, Standard Deviation of the Averages of NN intervals; SD‐5 min, Standard Deviation over 5 min; SDNN, Standard Deviation of Normal‐to‐Normal Intervals; SE, sleep efficiency; SFI, sleep fragmentation index; SL, sleep latency; SD‐24 h, Standard Deviation over 24 h; SQLFHF, Square Root of LF/HF Ratio; SWS, slow‐wave sleep; Tachygastria (%), percentage of slow waves in the tachygastria range (> 3.7–10 cycles per minute) [50]; TIB, time in bed; TIDM, Type I Diabetes Mellitus; TIIDM, Type II Diabetes Mellitus; TP, Total Power; TST, total sleep time; VLF, Very Low Frequency; WASO, wake after sleep onset.

^a^ Included in the meta‐analyses.

^b^ Although these studies compared DGBI and HCs, they were excluded from meta‐analyses due to missing data or inconsistencies in the units of the outcomes of interest.

^c^ This study was excluded from the meta‐analyses due to duplication of data from another study.

^d^ Studies assessing autonomic function in relation to GI symptom severity.

**FIGURE 4:**
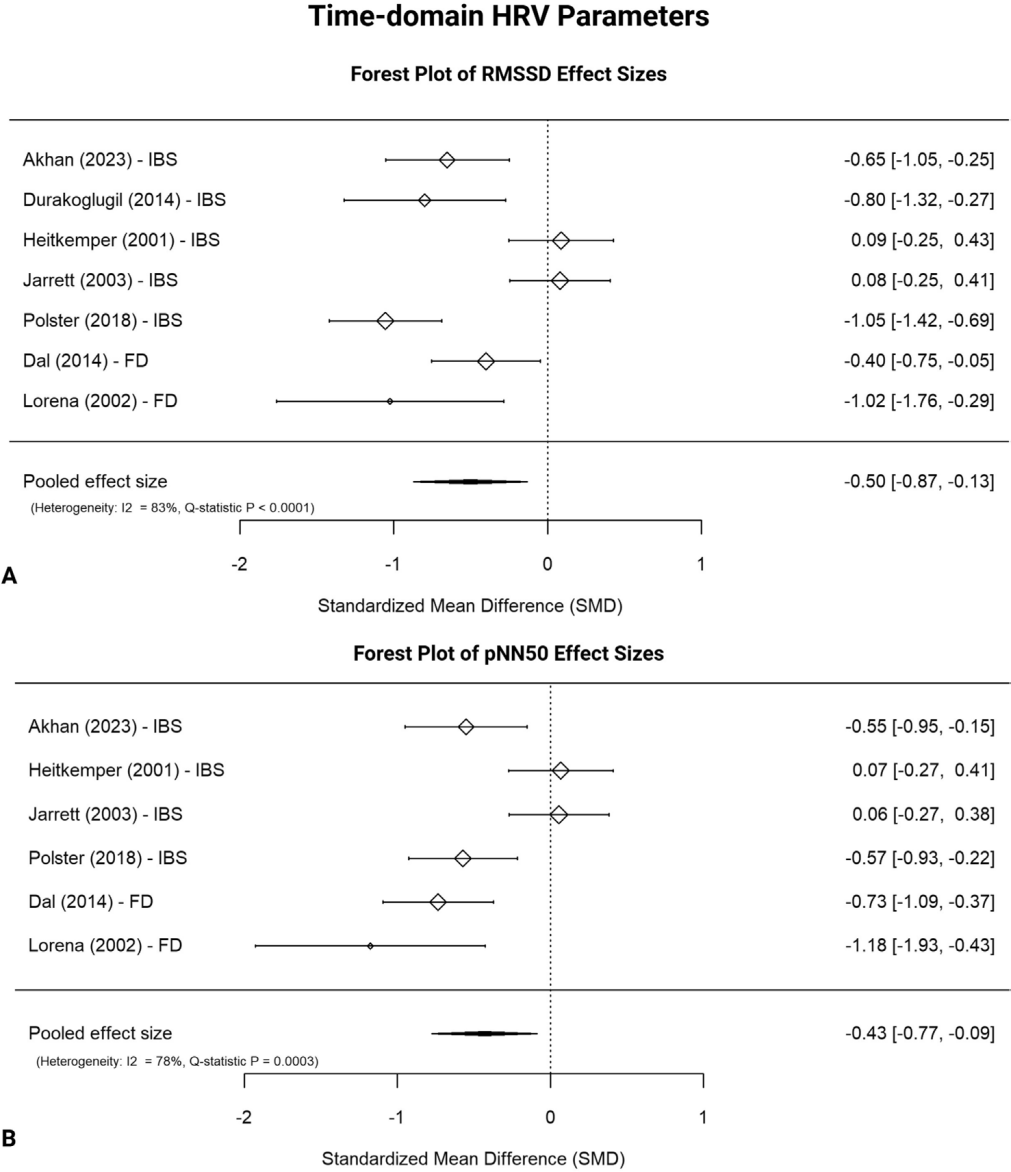
Forest plots of time‐domain HRV parameters. The forest plots display individual and pooled effect sizes for studies investigating time‐domain HRV parameters. A negative SMD indicates lower values in DGBI compared with healthy controls. Between‐study heterogeneity is indicated by *I* ^2^ and *Q*‐statistics. (A) RMSSD effect sizes. (B) pNN50 effect sizes. FD, functional dyspepsia; HRV, heart rate variability; IBS, irritable bowel syndrome; pNN50, percentage of successive RR intervals differing by more than 50 ms; RMSSD, Root Mean Square of Successive Differences between RR intervals; SMD, standardized mean difference.

**FIGURE 5:**
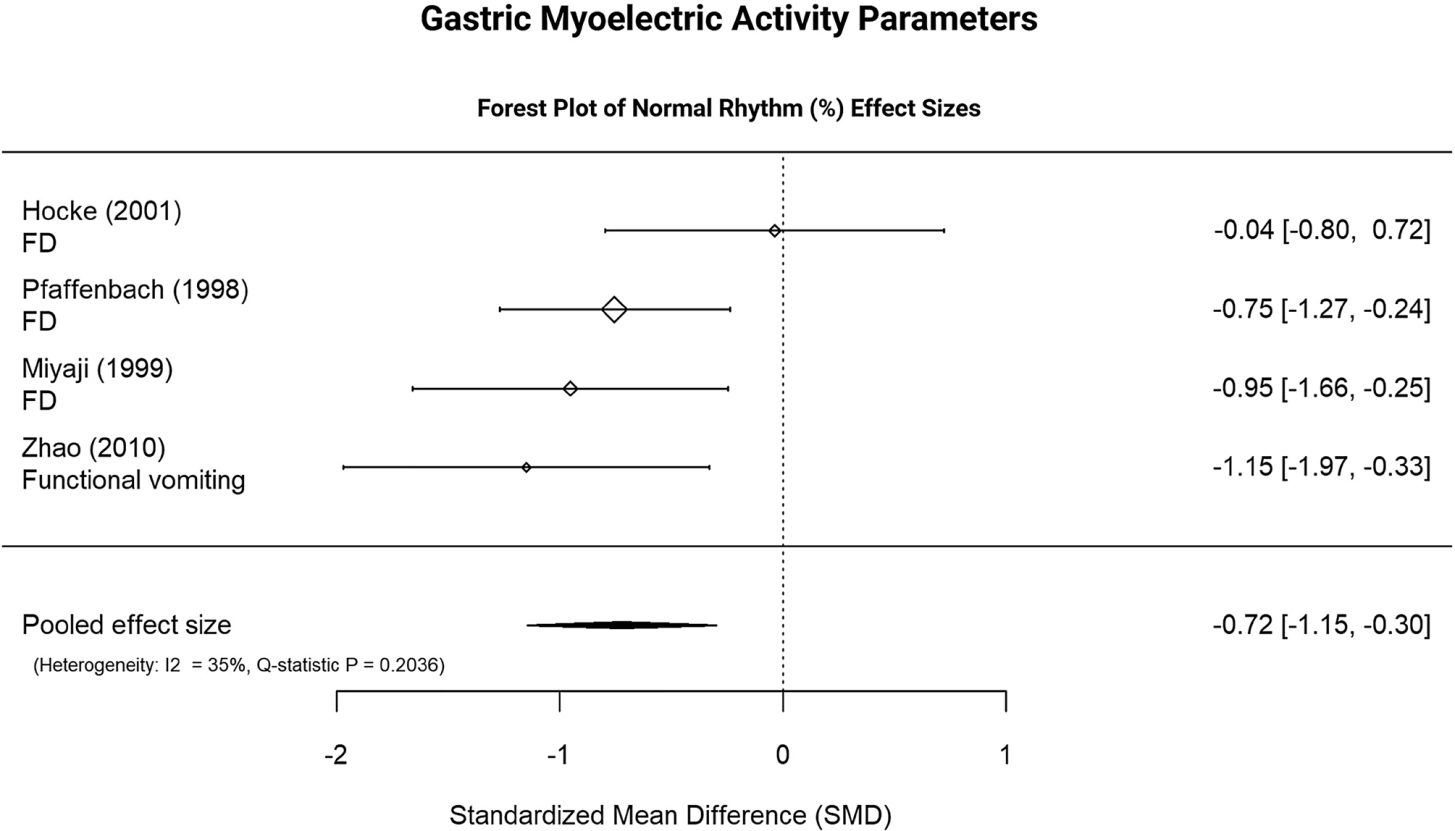
Forest Plot of Gastric Slow Wave Activity. The forest plot displays individual and pooled effect sizes for studies investigating the percentage of gastric slow wave activity. A negative SMD indicates lower values in DGBI compared with healthy controls. Between‐study heterogeneity is indicated by *I* ^2^ and *Q*‐statistics. FD, functional dyspepsia; Normal rhythm (%), percentage of gastric slow waves within the normal range (2.5–3.7 cycles per minute); SMD, standardized mean difference.

Meta‐analyses on the root mean square of the successive differences between RR intervals (RMSSD, 7 studies: IBS *n* = 5; FD *n* = 2) and percentage of successive RR intervals differing by more than 50 ms (pNN50, 6 studies: IBS *n* = 4, FD *n* = 2) yielded moderate negative pooled effect sizes of −0.503 (SE = 0.189, 95% CI [−0.873, −0.132], *I* ^2^ = 82.82%, *Q*‐statistic *p* < 0.001, Figure 4A) and −0.430 (SE = 0.176, 95% CI [−0.775, −0.085], *I* ^2^ = 78.47%, *Q*‐statistic *p* < 0.001, Figure 4B), respectively, indicating lower values in DGBI versus HCs. Other HRV metrics showed small effect sizes (range of SMDs −0.398 to 0.017), with no consistent differences between groups. See Table 3, Figures 3 and 5, and Supporting Information 2.4 for details.

**TABLE 3:** Pooled effect sizes for time‐domain and frequency‐domain HRV, sleep, and gastric myoelectric activity parameters in DGBI compared to healthy controls.

	Total included studies (*N*=)	SMD	SE	95% CI	*p*	*I* ^2^	*Q*‐statistic (*p*)
HRV: Time‐domain parameters							
RMSSD	7	−0.503	0.189	−0.873, −0.132	0.008	82.82%	< 0.001
pNN50	6	−0.430	0.176	−0,775, −0.085	0.015	78.47%	< 0.001
SDNN^a^	4	−0.398	0.308	−1.001, 0.206	0.197	85.85%	< 0.001
HRV: Frequency‐domain parameters							
HF power	8	−0.050	0.190	−0.422, 0.321	0.791	79.78%	< 0.001
LF power	6	−0.230	0.165	−0.553, 0.093	0.162	65.78%	0.012
LF/HF ratio	8	0.017	0.079	−0.137, 0.172	0.826	0.00%	0.692
Sleep							
TST^a^	3	1.295	1.175	−1.008, 3.597	0.271	95.50%	< 0.001
Gastric myoelectric activity							
DF^a^	4	−0.089	0.323	−0.722, 0.544	0.783	85.62%	< 0.001
Normal rhythm (%)^a^	4	−0.722	0.216	−1.146, −0.298	0.001	34.78%	0.204
Bradygastria (%)^a^	3	0.326	0.189	−0.043, 0.696	0.084	0.00%	0.436
Tachygastria (%)^a^	3	0.234	0.437	−0.623, 1.091	0.593	78.53%	0.010

*Note:* Negative SMD values indicate lower values of that parameter for individuals with DGBI compared to HCs. A *p*‐value < 0.05 indicates statistical significance. *I* ^2^ and *Q*‐statistics were used to assess the presence of heterogeneity. Bradygastria (%), percentage of slow waves in the bradygastria range (< 2.5 cycles per minute) [50]; 95% CI, 95% confidence intervals; DF, dominant frequency; HF, high frequency; LF, low frequency; LF/HF ratio, low frequency and high frequency ratio; Normal rhythm (%), percentage of gastric slow waves within the normal range (2.5–3.7 cycles per minute) [50]; pNN50, percentage of successive RR intervals differing by more than 50 ms; RMSSD, root mean square of successive differences between RR‐intervals; SDNN, standard deviation of normal‐to‐normal intervals; SE, standard error; SMD, standardized mean difference. Tachygastria (%), percentage of slow waves in the tachygastria range (> 3.7–10 cycles per minute) [50]; TST, total sleep time.

^a^ These analyses were descriptive in nature.

### 3.4 Sleep

Seven studies examined sleep in DGBI (*n* = 1803), including IBS (*n* = 146, 4 studies), FD (*n* = 107, 1 study), and functional constipation (*n* = 1550, 2 studies), using wrist‐mounted actigraphy (5 studies), Fitbit (1 study), or Sleepthing (1 study). Reported outcomes included total sleep time (TST, 6 studies), sleep efficiency (SE, 5), sleep latency (SL, 4), wake after sleep onset (WASO, 5), and number of awakenings (2) (Table 2). Exploratory meta‐analysis on TST showed no differences between DGBI and HCs (SMD = 1.295, SE = 1.175, 95% CI [−1.008, 3.597], *I* ^2^ = 95.90%, *Q*‐statistic *p* < 0.0001); Table 3 and Supporting Information 2.5.

### 3.5 Gastric Myoelectric Activity

Fourteen studies assessed GI function in DGBI (*n* = 1078), including IBS (*n* = 10, 1 study), FD (*n* = 845, 7 studies), functional vomiting (*n* = 19, 1 study), chronic nausea and vomiting syndrome ([CNVS], *n* = 93, 2 studies), and chronic gastroduodenal symptoms (*n* = 111, 3 studies), using ambulatory electrogastrography (*n* = 13, 4 BSGM, 1 wireless motility patches) (Table 2). Reported outcomes included dominant frequency, dominant power, percentage of normal gastric slow waves, bradygastria, tachygastria, and power ratio.

Exploratory meta‐analyses on the percentage of normal gastric slow waves (4 studies: FD *n* = 3, functional vomiting *n* = 1) showed lower values in DGBI versus HCs (SMD = −0.722, SE = 0.216, 95% CI [−1.146, −0.298], *I* ^2^ = 34.78%, *Q*‐statistic *p* = 0.204) (Figure 5). Other GI metrics showed no consistent differences (range of SMDs −0.089 to 0.326) Table 3 and Supporting Information 2.6.

### 3.6 Relationship Between Wearable‐Detected Autonomic and GI Function and GI Symptom Severity

Twenty‐three studies examined the relationship between autonomic function and GI symptoms: nine on HRV, six on sleep, and nine on gastric myoelectric activity, with one study assessing both HRV and sleep [53] (Supporting Information 2.7). HRV was studied in IBS (7 studies), FD (1), and functional constipation (1); sleep in IBS (4), FD (1), and functional constipation (1); and gastric myoelectric activity in FD (4), functional vomiting (1), CNVS (2), and chronic gastroduodenal symptoms (2).

**FIGURE 6:**
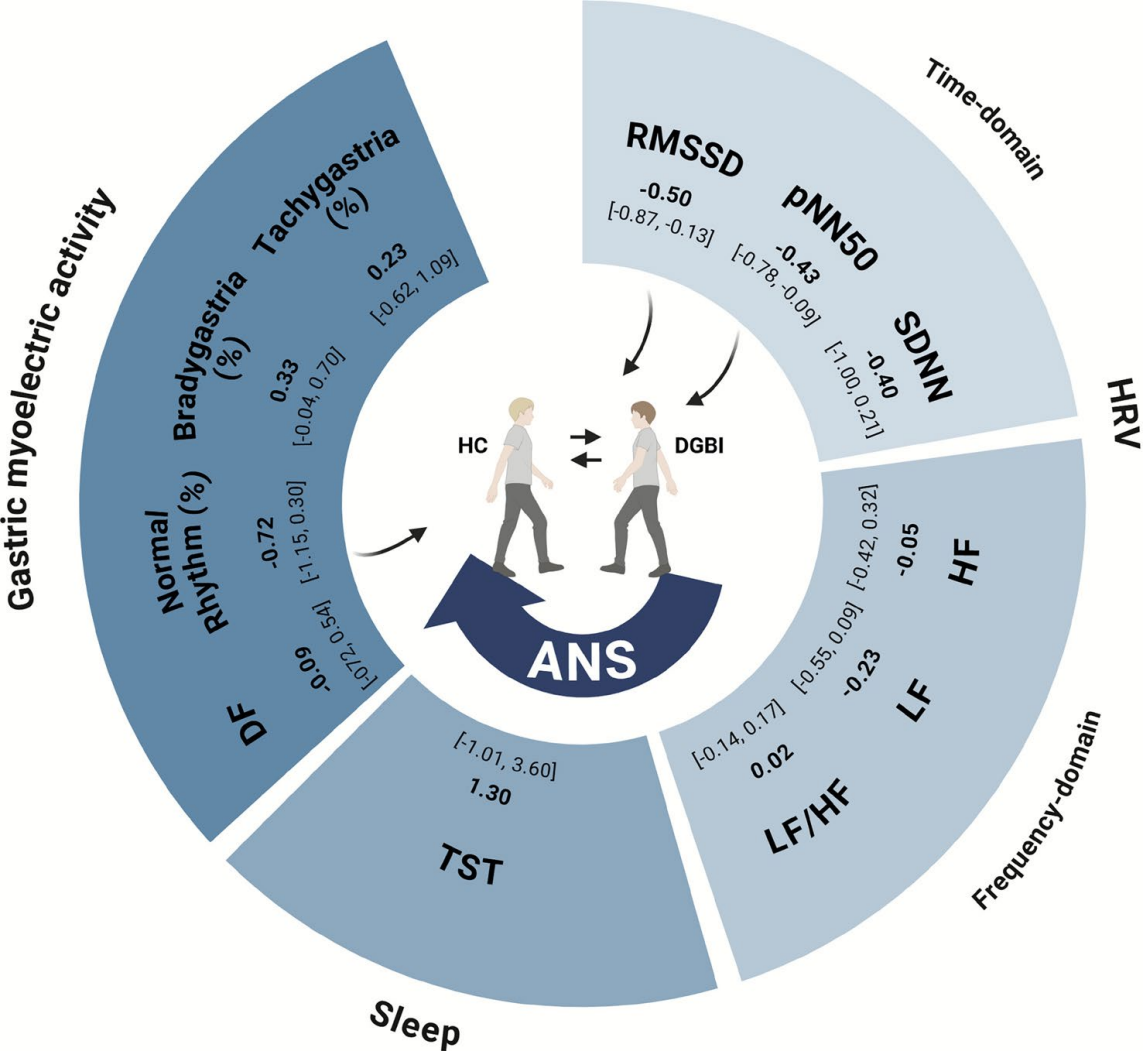
Overview of differences in autonomic and GI function between DGBI and healthy controls measured with wearable devices. Meta‐analyses results comparing autonomic and GI function between DGBI and HCs, using wearable devices. Parameters include time‐domain and frequency‐domain HRV, sleep, and gastric myoelectric activity. Pooled effect sizes with 95% confidence intervals are shown, with negative values indicating lower values in the DGBI group compared to HCs. Arrows pointing to the center indicate metrics with consistently observed differences between groups. This figure was made with BioRender. Bradygastria (%), percentage of slow waves in the bradygastria range (< 2.5 cycles per minute); DF, dominant frequency; DGBI, disorder of gut‐brain interaction; HC, healthy control; HF, high frequency; HRV, heart rate variability; LF, low frequency; LF/HF ratio, low frequency and high frequency ratio; normal rhythm (%), percentage of gastric slow waves within the normal range (2.5–3.7 cycles per minute); pNN50, percentage of successive RR intervals differing by more than 50 ms; RMSSD, Root Mean Square of Successive Differences between RR‐intervals; SDNN, Standard Deviation of Normal‐to‐Normal Intervals; Tachygastria (%), percentage of slow waves in the tachygastria range (> 3.7–10 cycles per minute); TST, total sleep time.

Due to substantial heterogeneity in symptom assessment across studies, no meta‐analyses were conducted. Across HRV studies, more severe IBS symptoms, particularly in constipation‐predominant IBS, were associated with reduced parasympathetic activity (e.g., lower RMSSD and pNN50) and increased sympathetic dominance (e.g., elevated LF/HF ratio) [54, 55, 56, 57, 58, 59]. In IBS and functional constipation, greater symptom severity was also linked to poorer sleep, although findings were inconsistent [53, 60, 61]. Abnormal EEG/BSGM patterns were associated with higher symptom severity in FD, functional vomiting, CNVS, and chronic gastroduodenal symptoms [62, 63, 64, 65, 66]. See Table 2, Figure 6, and Supporting Information 2.7 for an overview of the main results.

## 4 Discussion

The use of wearables in gastroenterology has increased with recent technological advances, yet their exact (diagnostic) value remains undetermined. This review provides the first comprehensive synthesis of wearables for assessing autonomic and GI functions in DGBI, highlighting important insights and research gaps. The meta‐analysis showed reduced time‐domain HRV parameters (RMSSD, pNN50) in DGBI, suggesting impaired vagal tone. Gastric slow wave activity has primarily been investigated in FD patients, where it was found to be reduced compared to HCs. Frequency‐domain HRV, sleep and other gastric myoelectric activity metrics showed no consistent differences versus HC. Although some studies point toward a link between altered HRV, impaired sleep, and abnormal gastric myoelectric activity with increased symptom severity, this could not be confirmed due to data heterogeneity.

Our findings underscore the complex and potentially multifaceted role of ANS and GI dysfunction in DGBI. The review summarized available wearable devices for assessing these functions, each using different technologies. However, the limited number of studies per metric reduced the power for thorough analysis. While HRV, sleep, and gastric myoelectric activity are intrinsically related to autonomic function, they exhibit inconsistent patterns across studies, complicating interpretation. This heterogeneity limits conclusions about whether ANS dysfunction constitutes a core feature of DGBI or is secondary to other underlying mechanisms.

The observed reduction in time‐domain HRV metrics (i.e., RMSSD, pNN50) in DGBI suggests vagal withdrawal, aligning with previous wearable‐based studies, particularly in IBS, pointing to the potential involvement of autonomic pathways in its pathophysiology [42]. Nonetheless, such alterations are not consistently reported across the broader DGBI population [59, 67, 68]. Instead, changes in both time‐ and frequency‐domain HRV metrics are more frequently observed in specific IBS subgroups, categorized by predominant bowel pattern [27, 58, 67, 69, 70], symptom severity [58, 71], or mental health comorbidities [22, 42]. Centrally mediated autonomic dysfunction has also been suggested in individuals with overlapping DGBI, who often present with more severe symptoms and psychological comorbidities, potentially contributing to inconsistent HRV findings across DGBI [72, 73]. Subgroup analyses were not feasible in our study due to limited power.

Notably, time‐domain HRV values showed moderate reductions in DGBI, whereas frequency‐domain metrics showed no consistent reductions. Time‐domain metrics reflect short‐term variability in heart rate and autonomic regulation, while frequency‐domain metrics assess the distribution of power across different frequency bands [74]. While both offer insights into ANS function, time‐domain metrics are often considered more clinically relevant, as they are less affected by variations in breathing [75, 76, 77]. Importantly, HRV alterations may also reflect changes in neuroendocrine regulation [42]. Elevated basal catecholamines levels and dysregulation of the hypothalamic–pituitary–adrenal (HPA) axis, characterized by heightened stress responsiveness, have been described in DGBI and strongly correlate with HRV changes [42, 78, 79, 80].

Sleep was assessed solely by total sleep time, as sufficient data on other objective metrics, such as sleep efficiency, were lacking. TST did not differ consistently in DGBI versus HCs. However, TST alone does not fully capture sleep quality, which may be relevant in DGBI [81, 82]. As a result, no firm conclusions can be drawn regarding sleep disturbances as a proxy for autonomic and GI dysfunction. Nevertheless, accumulating evidence suggests that sleep disruptions may heighten visceral hypersensitivity [60, 83, 84, 85, 86]. Given that the ANS plays a central role in modulating visceral hypersensitivity, sleep disruptions may represent an important mechanism underlying symptom generation [42, 83]. However, due to the multifactorial nature of sleep, disturbances may also be influenced by psychological, behavioral, and physiological contributors [60, 86]. Topan et al. [87] further showed that subjective, rather than objective, sleep quality was associated with greater next‐day GI symptoms in IBS, suggesting that the perception of sleep is of greater relevance.

Exploratory findings on gastric myoelectric activity, primarily driven by ENS activity, indicated reduced gastric slow wave activity in DGBI, particularly in FD. This aligns with previous studies using invasive and non‐invasive gastric mapping, which identified slow wave abnormalities in patients with FD and chronic unexplained nausea and vomiting [64, 88, 89, 90]. In contrast, dominant frequency and dysrhythmia markers (i.e., bradygastria and tachygastria) did not differ consistently between DGBI and HCs, implying that these parameters may be less sensitive or specific indicators of gastric dysfunction [65, 91]. Notably, included studies used different technical approaches (i.e., EGG, BSGM, wearable motility patches) each differing in accuracy, artifact susceptibility, and validation [64, 65, 66, 91]. While BSGM offers improved spatial resolution over traditional EGG, its clinical utility is still under evaluation [88, 92]. As such, it remains uncertain whether these technologies are truly comparable or interchangeable.

No eligible studies on skin conductivity met our criteria, likely reflecting the limited use of electrodermal activity (EDA) in DGBI research to date. This is potentially due to its limited specificity for GI autonomic processes, as EDA primarily reflects sympathetic arousal to emotional stimuli rather than visceral changes [49]. Methodological challenges, such as susceptibility to ambient temperature, movement artifacts, electrode placement, and skin hydration, may further limit its reliability in research settings [49, 93].

Findings from this review suggest a potential complex interplay between altered autonomic and GI function and symptom severity in DGBI, although the strength and consistency of this association vary across subtypes. In IBS, reduced parasympathetic activity and increased sympathetic dominance, as reflected in HRV, were associated with greater symptom severity and distinct bowel patterns [58, 59]. In contrast, no such associations were observed in FD [94]. Cain et al. [58] proposed that gut pain and HRV alterations may be linked to centrally mediated mechanisms, inherent to vagal withdrawal and sympathetic overactivity, which could contribute to downstream alterations in bowel habits. Supporting this, Kano et al. [95] and Wilder‐Smith et al. [96] showed, using functional magnetic resonance imaging, that IBS patients with different bowel patterns exhibited distinct brain activity responses to rectal distention. Weaver et al. [97] showed that these differences are observed in brain regions implicated in autonomic regulation and descending pain modulation, suggesting subtype‐specific central processing that may not necessarily generalize to other DGBI.

Our findings highlight both the potential and challenges of using wearables to assess autonomic and GI dysfunction in DGBI. While promising, their clinical implementation requires careful consideration of both advantages and limitations. Their potential lies in enabling continuous, non‐invasive monitoring of autonomic function, with real‐time data collection and communication between sensors and health care providers [9, 29, 41]. As such, these devices may aid in diagnosing, symptom tracking, and monitoring individual treatment responses in DGBI, assuming they adequately reflect pathological mechanisms contributing to symptom generation [41, 98]. In addition, wearables may reduce healthcare costs through fewer unnecessary hospitalizations and shorter inpatient stays [41, 99, 100], while also supporting behavioral change through ongoing feedback, ultimately improving health outcomes [41, 101].

However, the implementation of wearable devices presents several challenges. High device costs may limit accessibility [44, 99, 101]. Furthermore, their effective use relies on patient motivation, as consistent wearing and data transfer are required [101]. Accurate monitoring of autonomic and GI functions is essential, yet many of the available wearable technologies have not been adequately validated, and their accuracy may vary depending on the metric assessed [101, 102, 103]. For instance, time‐domain HRV metric RMSSD has demonstrated strong agreement with ECG‐derived data [104, 105], while spectral measures like LF/HF ratio show less consistency [103, 106, 107]. Furthermore, current wearables primarily detect systemic autonomic dysfunction, and may miss focal disturbances (e.g., vagal injury following fundoplication). Additionally, factors such as caffeine, physical activity, and posture influence ANS measurements, complicating interpretation [9] and require standardization [103, 108]. Privacy concerns arise from the collection and storage of large quantities of personal health data, often accessible to third parties, requiring robust data protection measures [41, 44, 99]. As such, clinical implementation awaits further investigation of cost‐effectiveness, usability, data accuracy, and privacy safeguards.

In light of these broader considerations, several limitations of this review must be acknowledged. First, only three RCTs were included, and the limited number of studies available per parameter likely limited the robustness of meta‐analytic findings. The included studies varied in DGBI subtype (using different Rome criteria), wearable devices, study designs, and measurement protocols, contributing to the high heterogeneity observed. The studies span 1997–2024, a period during which wearable technology has evolved substantially. Devices used in early research likely had lower signal quality and fewer features compared with more recent devices. Additionally, some studies used consumer‐grade devices (e.g., Fitbit) that have not been formally validated for the physiological outcomes measured. Together, these factors could have contributed to the heterogeneity and affected the interpretation of the results. Figure 3 reflects this uncertainty, illustrating that while autonomic and GI measurement using wearables is technically feasible and shows potential, its accuracy and clinical utility in DGBI remain insufficiently established. Data extraction was further complicated by inconsistent units, missing data, and variations in prandial state. Most studies had a moderate risk of bias, with some at serious risk due to the omission of relevant confounders and poor reporting of missing outcomes. Sensitivity analyses were not conducted due to the limited number of studies, which may have influenced results.

In conclusion, this review provides a comprehensive overview of wearable devices for assessing autonomic and GI function in DGBI, highlighting their potential in this field. Exploratory meta‐analyses suggest promise, particularly in the context of HRV, though methodological variability and limited data restrict the ability to draw firm conclusions. Further research with standardized protocols and larger samples is needed to confirm their accuracy and clinical utility.

## Author Contributions

M.B., S.M., and D.K. initiated the study concept and design. F.V. and M.B. conducted the search, selected the articles, and collected the data. F.V. analyzed and interpreted the data. F.V. drafted the manuscript and created the graphics and visualization. M.B., A.R., S.M., and D.K. provided constructive feedback on the manuscript. D.K. supervised the project. All authors approved the final manuscript.

## Funding

This work was supported by the Horizon Europe Grant ERC‐2022‐STG RESILIENCE (Grant Agreement No. 101075884), funded by the European Research Council (ERC).

## Disclosure

Contributors who do not meet the criteria for authorship: Valentina Secchi and Ioana Seteanu, who assisted in developing an effective search strategy.

## Conflicts of Interest

F.V., M.B., A.R., and S.M.: None to declare. D.K: Has received research funding from ZonMw, Dutch Foundation for Gastroenterology, United European Gastroenterology, Horizon 2020, Horizon Europe, Rome Foundation, and speaker's fee from Rome Foundation (paid to host institute).

## Supporting information


**Data S1:** Supporting Information.

## Data Availability

Additional data are available in the Supporting Information.
