# Supplementary material for: Wearable‐Based Monitoring of Autonomic and Gastrointestinal Function in Disorders of Gut‐Brain Interaction: A Systematic Review and Meta‐Analyses
Source: Neurogastroenterol Motil. 2026 Jan 19;38(1):e70232. doi: 10.1111/nmo.70232 (PMC12815003; doi:10.1111/nmo.70232)
Supplement: Supplementary file 1 — Data S1: Supporting Information. [file NMO-38-e70232-s001.docx]

**Supplementary Data**

**Table of contents**

1.Methods……………………………………………………………………………………………………………2-6

1.1 Search………………………………………………………………………………………………………..2-4

1.2 Data extraction…………………………………………………………………………………………….4-5

1.3 Risk of bias assessment…………………………………………………………………………………5

1.4 Outcome measures………………………………………………………………………………………5-6

1.5 Statistical Analysis………………………………………………………………………………………..6

2.Results……………………………………………………………………………………………………………..7-44

2.1 Study characteristics…………………………………………………………………………………….7-13

2.2 Patient characteristics…………………………………………………………………………………..14-17

2.3 Risk of bias………………………………………………………………………………………………….18-22

2.4 Heart Rate Variability…………………………………………………………………………………….23-32

2.5 Sleep………………………………………………………………………………………………………….33-35

2.6 Gastric Myoelectric Activity……………………………………………………………………………36-42

2.7 Relationship between autonomic function and GI symptom severity……………………43-44

3.References……………………………………………………………………………………………………….45-47

1. **Methods**
   1. **Search**

Databases: PubMed, EMBASE, and Cochrane Library.

Last searched on 12 November 2024.

| **HRV** |
| --- |
| *Search:*  "Colonic Diseases, Functional"[Mesh] OR "functional colonic disease*"[tiab] OR "functional gastrointestinal*"[tiab] OR "Gastrointestinal function disorder*"[tiab] OR "FGID*"[tiab] OR "brain-gut*"[tiab] OR "gut-brain*"[tiab] OR "DGBI*"[tiab] OR "Irritable Bowel Syndrome"[Mesh] OR "irritable bowel syndrome*"[tiab] OR "IBS"[tiab] OR "Irritable colon*"[tiab] OR "Spastic colon*"[tiab] OR "Dyspepsia"[Mesh] OR "dyspep*"[tiab] OR "functional dyspepsia*"[tiab] OR "FD"[tiab] OR "Constipation"[Mesh:NoExp] OR "Constipation"[tiab] OR "functional constipation*"[tiab] OR "functional bowel disorder*"[tiab] OR "Chronic nausea and vomiting syndrome*"[tiab]  AND  "Autonomic Nervous System*"[Mesh] OR "autonomic nervous system*"[tiab] OR "ANS"[tiab] OR "vegetative nervous system*"[tiab] OR "Visceral nervous system*"[tiab] OR "autonomic function*"[tiab] OR "autonomic dysfunction*"[tiab] OR "Dysautonomia"[tiab] OR "autonomic imbalance*"[tiab] OR "Heart Rate"[Mesh] OR "heart rate*"[tiab] OR "Cardiac rate*"[tiab] OR "heart rate variability*"[tiab] OR "HRV"[tiab] OR "pulse*"[tiab] OR "heartbeat*"[tiab] OR "cardiac chronotrop*"[tiab] OR "Heart Rate Determination"[Mesh] OR "Heart rate*"[tiab] OR "Pulse rate*"[tiab]  AND  "Wearable Electronic Devices"[Mesh] OR "wearable electronic device*"[tiab] OR "wearable*"[tiab] OR "wrist wearable*"[tiab] OR "wristband*"[tiab] OR "Fitbit*"[tiab] OR "Apple Watch*"[tiab] OR "Garmin*"[tiab] OR "Tracker*"[tiab] OR "smartwatch*"[tiab] OR "Smartphone"[Mesh] OR "smartphone*"[tiab] OR "Mobile Applications"[Mesh] OR "mobile application*"[tiab] OR "application*"[tiab] OR "Medical Informatics Applications"[Mesh] OR "medical informatics application*"[tiab] OR "Monitoring, Physiologic"[Mesh] OR "Monitoring, Ambulatory"[Mesh] OR "monitoring*"[tiab] OR "Wireless Technology"[Mesh] OR "wireless technolog*"[tiab] OR "Telemedicine"[Mesh] OR "virtual medicine*"[tiab] OR "telehealth"[tiab] OR "eHealth"[tiab] OR "Mobile health"[tiab] OR "mhealth"[tiab] OR "Electrocardiography"[Mesh] OR "Electrocardiogra*"[tiab] OR "ecg"[tiab] OR "ekg"[tiab] OR "holter*"[tiab] OR "Cardiomedive"[tiab] |
| **Sleep** |
| *Search:*  "Colonic Diseases, Functional"[Mesh] OR "functional colonic disease*"[tiab] OR "functional gastrointestinal*"[tiab] OR "Gastrointestinal function disorder*"[tiab] OR "FGID*"[tiab] OR "brain-gut*"[tiab] OR "gut-brain*"[tiab] OR "DGBI*"[tiab] OR "Irritable Bowel Syndrome"[Mesh] OR "irritable bowel syndrome*"[tiab] OR "IBS"[tiab] OR "Irritable colon*"[tiab] OR "Spastic colon*"[tiab] OR "Dyspepsia"[Mesh] OR "dyspep*"[tiab] OR "functional dyspepsia*"[tiab] OR "FD"[tiab] OR "Constipation"[Mesh:NoExp] OR "Constipation"[tiab] OR "functional constipation*"[tiab] OR "functional bowel disorder*"[tiab] OR "Chronic nausea and vomiting syndrome*"[tiab] OR "NVS"[tiab] OR "Gastric dysfunction*"[tiab] OR "Chronic gastroduodenal disorder*"[tiab]  AND  "Autonomic Nervous System*"[Mesh] OR "autonomic nervous system*"[tiab] OR "ANS"[tiab] OR "vegetative nervous system*"[tiab] OR "Visceral nervous system*"[tiab] OR "autonomic function*"[tiab] OR "autonomic dysfunction*"[tiab] OR "Dysautonomia"[tiab] OR "autonomic imbalance*"[tiab] OR "Sleep"[Mesh] OR "sleep*"[tiab] OR "REM"[tiab] OR "NREM"[tiab]  AND  "Wearable Electronic Devices"[Mesh] OR "wearable electronic device*"[tiab] OR "wearable*"[tiab] OR "wrist wearable*"[tiab] OR "wristband*"[tiab] OR "Fitbit*"[tiab] OR "Apple Watch*"[tiab] OR "Garmin*"[tiab] OR "Tracker*"[tiab] OR "smartwatch*"[tiab] OR "Smartphone"[Mesh] OR "smartphone*"[tiab] OR "Mobile Applications"[Mesh] OR "mobile application*"[tiab] OR "application*"[tiab] OR "Medical Informatics Applications"[Mesh] OR "medical informatics application*"[tiab] OR "Monitoring, Physiologic"[Mesh] OR "Monitoring, Ambulatory"[Mesh] OR "monitoring*"[tiab] OR "Wireless Technology"[Mesh] OR "wireless technolog*"[tiab] OR "Telemedicine"[Mesh] OR "virtual medicine*"[tiab] OR "telehealth"[tiab] OR "eHealth"[tiab] OR "Mobile health"[tiab] OR "mhealth"[tiab] OR "Polysomnography"[Mesh] OR "Polysomnogra*"[tiab] OR "pst"[tiab] OR "sleep monitor*"[tiab] OR "Somnogra*"[tiab] OR "electroencephalography"[Mesh] OR "Electroencephalogra*"[tiab] OR "eeg"[tiab] OR "Electrooculography"[Mesh] OR "Electrooculogra*"[tiab] OR "eog"[tiab] OR "ACCEL Star*"[tiab] |
| **GI function** |
| *Search:*  "Colonic Diseases, Functional"[Mesh] OR "functional colonic disease*"[tiab] OR "functional gastrointestinal*"[tiab] OR "Gastrointestinal function disorder*"[tiab] OR "FGID*"[tiab] OR "brain-gut*"[tiab] OR "gut-brain*"[tiab] OR "DGBI*"[tiab] OR "Irritable Bowel Syndrome"[Mesh] OR "irritable bowel syndrome*"[tiab] OR "IBS"[tiab] OR "Irritable colon*"[tiab] OR "Spastic colon*"[tiab] OR "Dyspepsia"[Mesh] OR "dyspep*"[tiab] OR "functional dyspepsia*"[tiab] OR "FD"[tiab] OR "Constipation"[Mesh:NoExp] OR "Constipation"[tiab] OR "functional constipation*"[tiab] OR "functional bowel disorder*"[tiab] OR "Chronic nausea and vomiting syndrome*"[tiab] OR "NVS"[tiab] OR "Gastric dysfunction*"[tiab] OR "Chronic gastroduodenal disorder*"[tiab]  AND  "Autonomic Nervous System*"[Mesh] OR "autonomic nervous system*"[tiab] OR "ANS"[tiab] OR "vegetative nervous system*"[tiab] OR "Visceral nervous system*"[tiab] OR "autonomic function*"[tiab] OR "autonomic dysfunction*"[tiab] OR "Dysautonomia"[tiab] OR "autonomic imbalance*"[tiab] OR "Gastrointestinal sound*"[tiab] OR "Bowel sound*"[tiab] OR "gastrointestinal activity"[tiab] OR "Gastric Myoelectric Activity"[tiab] OR "Gastric electrical activity"[tiab] OR "gastric motilit*"[tiab] OR "Gastric emptying"[tiab] OR "gastric function*"[tiab] OR "Meal response*"[tiab]  AND  "Wearable Electronic Devices"[Mesh] OR "wearable electronic device*"[tiab] OR "wearable*"[tiab] OR "wrist wearable*"[tiab] OR "wristband*"[tiab] OR "Fitbit*"[tiab] OR "Apple Watch*"[tiab] OR "Garmin*"[tiab] OR "Tracker*"[tiab] OR "smartwatch*"[tiab] OR "Smartphone"[Mesh] OR "smartphone*"[tiab] OR "Mobile Applications"[Mesh] OR "mobile application*"[tiab] OR "application*"[tiab] OR "Medical Informatics Applications"[Mesh] OR "medical informatics application*"[tiab] OR "Monitoring, Physiologic"[Mesh] OR "Monitoring, Ambulatory"[Mesh] OR "monitoring*"[tiab] OR "Wireless Technology"[Mesh] OR "wireless technolog*"[tiab] OR "Telemedicine"[Mesh] OR "virtual medicine*"[tiab] OR "telehealth"[tiab] OR "eHealth"[tiab] OR "Mobile health"[tiab] OR "mhealth"[tiab] OR "Electromyography"[Mesh] OR "Electromyogra*"[tiab] OR "emg"[tiab] OR "Electrogastrogra*"[tiab] OR "Body Surface Potential Mapping"[Mesh] OR "Body Surface Mapping*"[tiab] OR "body surface gastric mapping*"[tiab] OR "Noninvasive mapping*"[tiab] OR "BSGM"[tiab] OR "Noninvasive gastric mapping*"[tiab] OR "gastric mapping device*"[tiab] OR "Gastric electrophysiology mapping*"[tiab] OR "Body Surface Colonic Mapping*"[tiab] OR "BSCM"[tiab] OR "Motility patch*"[tiab] OR "Wireless patch*"[tiab] OR "Patch*"[tiab] OR "Smart clothing*"[tiab] OR "Cutaneous electrogastrograph*"[tiab] OR "Gastric Alimetr*"[tiab] OR "Alimetr*"[tiab] |
| **Sweat / Skin conductivity** |
| *Search:*  "Colonic Diseases, Functional"[Mesh] OR "functional colonic disease*"[tiab] OR "functional gastrointestinal*"[tiab] OR "Gastrointestinal function disorder*"[tiab] OR "FGID*"[tiab] OR "brain-gut*"[tiab] OR "gut-brain*"[tiab] OR "DGBI*"[tiab] OR "Irritable Bowel Syndrome"[Mesh] OR "irritable bowel syndrome*"[tiab] OR "IBS"[tiab] OR "Irritable colon*"[tiab] OR "Spastic colon*"[tiab] OR "Dyspepsia"[Mesh] OR "dyspep*"[tiab] OR "functional dyspepsia*"[tiab] OR "FD"[tiab] OR "Constipation"[Mesh:NoExp] OR "Constipation"[tiab] OR "functional constipation*"[tiab] OR "functional bowel disorder*"[tiab] OR "Chronic nausea and vomiting syndrome*"[tiab] OR "NVS"[tiab] OR "Gastric dysfunction*"[tiab] OR "Chronic gastroduodenal disorder*"[tiab]  AND  "Autonomic Nervous System*"[Mesh] OR "autonomic nervous system*"[tiab] OR "ANS"[tiab] OR "vegetative nervous system*"[tiab] OR "Visceral nervous system*"[tiab] OR "autonomic function*"[tiab] OR "autonomic dysfunction*"[tiab] OR "Dysautonomia"[tiab] OR "autonomic imbalance*"[tiab] OR "Sweat"[Mesh] OR "Sweat*"[tiab] OR "sudomotor*"[tiab]  AND  "Wearable Electronic Devices"[Mesh] OR "wearable electronic device*"[tiab] OR "wearable*"[tiab] OR "wrist wearable*"[tiab] OR "wristband*"[tiab] OR "Fitbit*"[tiab] OR "Apple Watch*"[tiab] OR "Garmin*"[tiab] OR "Tracker*"[tiab] OR "smartwatch*"[tiab] OR "Smartphone"[Mesh] OR "smartphone*"[tiab] OR "Mobile Applications"[Mesh] OR "mobile application*"[tiab] OR "application*"[tiab] OR "Medical Informatics Applications"[Mesh] OR "medical informatics application*"[tiab] OR "Monitoring, Physiologic"[Mesh] OR "Monitoring, Ambulatory"[Mesh] OR "monitoring*"[tiab] OR "Wireless Technology"[Mesh] OR "wireless technolog*"[tiab] OR "Telemedicine"[Mesh] OR "virtual medicine*"[tiab] OR "telehealth"[tiab] OR "eHealth"[tiab] OR "Mobile health"[tiab] OR "mhealth"[tiab] OR "sweatsensor*"[tiab] OR "sweat test*"[tiab] OR "sudomotor test*"[tiab] OR "electrochemical skin conductance*"[tiab] OR "esc"[tiab] OR "quantitative sudomotor axon reflex test*"[tiab] OR "sart*"[tiab] OR "sweat sensor*"[tiab] |
| **Total search** |
| "Colonic Diseases, Functional"[Mesh] OR "functional colonic disease*"[tiab] OR "functional gastrointestinal*"[tiab] OR "Gastrointestinal function disorder*"[tiab] OR "FGID*"[tiab] OR "brain-gut*"[tiab] OR "gut-brain*"[tiab] OR "DGBI*"[tiab] OR "Irritable Bowel Syndrome"[Mesh] OR "irritable bowel syndrome*"[tiab] OR "IBS"[tiab] OR "Irritable colon*"[tiab] OR "Spastic colon*"[tiab] OR "Dyspepsia"[Mesh] OR "dyspep*"[tiab] OR "functional dyspepsia*"[tiab] OR "FD"[tiab] OR "Constipation"[Mesh:NoExp] OR "Constipation"[tiab] OR "functional constipation*"[tiab] OR "functional bowel disorder*"[tiab] OR "Chronic nausea and vomiting syndrome*"[tiab] OR "NVS"[tiab] OR "Gastric dysfunction*"[tiab] OR "Chronic gastroduodenal disorder*"[tiab]  AND  "Autonomic Nervous System*"[Mesh] OR "autonomic nervous system*"[tiab] OR "ANS"[tiab] OR "vegetative nervous system*"[tiab] OR "Visceral nervous system*"[tiab] OR "autonomic function*"[tiab] OR "autonomic dysfunction*"[tiab] OR "Dysautonomia"[tiab] OR "autonomic imbalance*"[tiab] OR "Gastrointestinal sound*"[tiab] OR "Bowel sound*"[tiab] OR "gastrointestinal activity"[tiab] OR "Gastric Myoelectric Activity"[tiab] OR "Gastric electrical activity"[tiab] OR "gastric motilit*"[tiab] OR "Gastric emptying"[tiab] OR "gastric function*"[tiab] OR "Meal response*"[tiab] OR "Sweat"[Mesh] OR "Sweat*"[tiab] OR "sudomotor*"[tiab] OR "Sleep"[Mesh] OR "sleep*"[tiab] OR "REM"[tiab] OR "NREM"[tiab] OR "Heart Rate"[Mesh] OR "heart rate*"[tiab] OR "Cardiac rate*"[tiab] OR "heart rate variability*"[tiab] OR "HRV"[tiab] OR "pulse*"[tiab] OR "heartbeat*"[tiab] OR "cardiac chronotrop*"[tiab]  AND  "Wearable Electronic Devices"[Mesh] OR "wearable electronic device*"[tiab] OR "wearable*"[tiab] OR "wrist wearable*"[tiab] OR "wristband*"[tiab] OR "Fitbit*"[tiab] OR "Apple Watch*"[tiab] OR "Garmin*"[tiab] OR "Tracker*"[tiab] OR "smartwatch*"[tiab] OR "Smartphone"[Mesh] OR "smartphone*"[tiab] OR "Mobile Applications"[Mesh] OR "mobile application*"[tiab] OR "application*"[tiab] OR "Medical Informatics Applications"[Mesh] OR "medical informatics application*"[tiab] OR "Monitoring, Physiologic"[Mesh] OR "Monitoring, Ambulatory"[Mesh] OR "monitoring*"[tiab] OR "Wireless Technology"[Mesh] OR "wireless technolog*"[tiab] OR "Telemedicine"[Mesh] OR "virtual medicine*"[tiab] OR "telehealth"[tiab] OR "eHealth"[tiab] OR "Mobile health"[tiab] OR "mhealth"[tiab] OR "Electromyography"[Mesh] OR "Electromyogra*"[tiab] OR "emg"[tiab] OR "Electrogastrogra*"[tiab] OR "Body Surface Potential Mapping"[Mesh] OR "Body Surface Mapping*"[tiab] OR "body surface gastric mapping*"[tiab] OR "Noninvasive mapping*"[tiab] OR "BSGM"[tiab] OR "Noninvasive gastric mapping*"[tiab] OR "gastric mapping device*"[tiab] OR "Gastric electrophysiology mapping*"[tiab] OR "Body Surface Colonic Mapping*"[tiab] OR "BSCM"[tiab] OR "Motility patch*"[tiab] OR "Wireless patch*"[tiab] OR "Patch*"[tiab] OR "Smart clothing*"[tiab] OR "Cutaneous electrogastrograph*"[tiab] OR "Gastric Alimetr*"[tiab] OR "Alimetr*"[tiab] OR "sweatsensor*"[tiab] OR "sweat test*"[tiab] OR "sudomotor test*"[tiab] OR "electrochemical skin conductance*"[tiab] OR "esc"[tiab] OR "quantitative sudomotor axon reflex test*"[tiab] OR "sart*"[tiab] OR "sweat sensor*"[tiab] OR "Polysomnography"[Mesh] OR "Polysomnogra*"[tiab] OR "pst"[tiab] OR "sleep monitor*"[tiab] OR "Somnogra*"[tiab] OR "ACCEL Star*"[tiab] OR "electroencephalography"[Mesh] OR "Electroencephalogra*"[tiab] OR "eeg"[tiab] OR "Electrooculography"[Mesh] OR "Electrooculogra*"[tiab] OR "eog"[tiab] OR "Heart Rate Determination"[Mesh] OR "Heart rate*"[tiab] OR "Pulse rate*"[tiab] OR "Electrocardiography"[Mesh] OR "Electrocardiogra*"[tiab] OR "ecg"[tiab] OR "ekg"[tiab] OR "holter*"[tiab] OR "Cardiomedive"[tiab] |

- 1. **Data Extraction**

Data extraction was independently performed by both researchers (FV, MB). Relevant study characteristics and outcome measures were systematically collected, including author, publication year, country, study design, recruitment method, total population, number of DGBI patients and HCs (if applicable), Rome criteria, autonomic parameters, wearable devices used, measurement duration, patient characteristics (age, gender, BMI), and outcome measures. Autonomic and GI parameters were compared between the studies, with overlapping variables within each parameter (e.g., Root Mean Square of Successive Differences [RMSSD] for HRV) extracted. Data were recorded as means with standard deviations (SD), and missing information was noted as ‘not reported’. In case of missing SDs, the mean SD from included studies was imputed. For gastric myoelectric activity, postprandial values were prioritized, as DGBI symptoms are frequently reported to be food-related (1).

- 1. **Risk of Bias Assessment**

The risk of bias of the eligible studies was reviewed by two researchers individually using the Cochrane risk of bias tool (RoB 2) (2) for randomized controlled trials (RCTs), and the ROBINS-I tool (3) for observational studies. Within the ROBINS-I tool, categories related to the measurement of interventions and deviations from intended interventions were not assessed, as the included studies focused on diagnostic measurements rather than interventions. Each domain was rated as having a low risk of bias, some concerns, or a high risk of bias for RoB2, and as having a low, moderate, or serious risk of bias for ROBINS-I. Conflicts in assessments were resolved through discussion and mutual agreement.

- 1. **Outcome measures**

HRV metrics were assessed using both time-domain and frequency-domain parameters, which have been developed to evaluate sympathetic and parasympathetic nervous system activity by analyzing RR interval signals (4). Time-domain indices capture variability in the length of interbeat intervals (IBI) and include root mean square of successive differences (RMSSD), the standard deviation of NN intervals (SDNN), The average of the standard deviations of all NN intervals for each 5-minute segment of a 24-hour recording (SDNN index), the standard deviation of the average NN intervals for each 5 min segment of a 24 h HRV recording (SDANN), the standard deviation of NN intervals within one 5-minute segment (SD-5min), and the percentage of successive RR intervals that differ by more than 50ms (pNN50) (5). Frequency-domain metrics quantify the distribution of absolute or relative power across four distinct frequency bands, including high-frequency power (HF), low-frequency power (LF), the LF/HF ratio, and total power (TP) (5, 6).

Objective sleep measures included total sleep time (TST), sleep efficiency (SE), sleep latency (SL), wake after sleep onset (WASO), and the number of awakenings, which are considered factors associated with a good night’s sleep (7, 8).

Gastric myoelectric activity was quantified using several parameters, including dominant frequency (DF) - the gold standard for evaluating gastric slow wave frequency - dominant power (DP), the percentage of normal gastric slow waves, bradygastria and tachygastria, and power ratio (9).

Objective measures of sweating included skin conductivity, which refers to the measurement of the electrical conductance of the skin. This physiological activation is induced by sympathetic nervous system activity, which influences moisture levels, and consequently skin conductance (10).

- 1. **Statistical Analysis**

To assess differences in HRV between DGBIs and HCs, meta-analyses were performed for time-domain metrics RMSSD, SDNN, and pNN50, and frequency-domain indices HF, LF, and LF/HF ratio. SDNN index and TP were excluded from the meta-analyses to avoid redundancy, as SDNN reflects overall HRV over longer periods, while SDNN index captures HRV over shorter segments. Additionally, LF and HF power are subsets of the total power spectrum (5).

Exploratory meta-analyses were conducted only for the sleep parameter TST, given the limited number of studies comparing DGBIs and HCs and the minimal overlap between key variables.

For gastric myoelectric activity, the variables included were DF, the percentage of normal gastric slow waves, and the percentages of bradygastria and tachygastria.

No studies were identified for sweating, and therefore, no analyses were performed.

For all metrics, data were analyzed using the same statistical approach, employing a random-effects model with standardized mean differences as the effect size.

1. **Results**
   1. **Study Characteristics**

**Table 1: Study characteristics**

| **Study** | **Country** | **Study design** | **Recruitment method*** | **Total study population (n =)** | **DGBI**  **(n =)** | **HC**  **(n =)** | **Rome criteria** | **Autonomic parameter** | **Wearable Device** | **Duration of measurement** | **Other interventions / measurements** |
| --- | --- | --- | --- | --- | --- | --- | --- | --- | --- | --- | --- |
| Akhan, et al. (2023) | Turkey | Non-RCT | Secondary | 100 | IBS  (50) | Yes  (50) | IV | HRV | Holter electrocardiography | 24 hours | None. |
| Cain, et al. (2006) | USA | Non-RCT | Primary | 215 | IBS  (165) | Yes  (50) | I | HRV | Holter electrocardiography | 24 hours | Daily diary on smoking, alcohol and caffeine consumption, medication use and stress. Questionnaires: BDQ, SCL-90, Hassles scale. |
| Chen, et al. (2024) | USA | Non-RCT | Primary and Secondary | 89 | IBS  (89) | No  ( - ) | III | HRV | Holter electrocardiography | 6 hours | 4-week daily symptom diary. |
| Durakoğlugil, et al. (2014) | Turkey | Non-RCT | Unknown | 60 | IBS  (30) | Yes  (30) | III | HRV | Holter electrocardiography | 24 hours | cf-PWV assessment, CIMT measurement, and biochemical measurements. |
| Heitkemper, et al. (1998) | USA | Non-RCT | Primary | 40 | IBS  (25) | Yes  (15) | Unknown | HRV | Holter electrocardiography | 24 hours | Daily symptom diaries for one menstrual cycle, urine samples, LH measurement. |
| Heitkemper, et al. (2001) | USA | Non-RCT | Primary | 152 | IBS  (103) | Yes  (49) | I | HRV | Holter electrocardiography | 24 hours | Laboratory tests of function (i.e., expiratory/inspiratory ratio, Valsalva, posture changes, and cold pressor).  Measurement of LH. Daily symptom diaries. Questionnaires: BDQ. |
| Jarrett, et al. (2003) | USA | Non-RCT | Primary | 209 | IBS  (163) | Yes  (46) | I | HRV | Holter electrocardiography | 24 hours | Daily symptom diaries. Questionnaires: DIS & SCL-90. |
| Jarrett, Cain, et al. (2016) | USA | RCT | Primary and Secondary | 85 | IBS  (85) | No  ( - ) | III | HRV | Holter electrocardiography | 8 hours | 8-week CSM intervention vs. usual care, with follow-up at 3- and 6-months post randomization. Biomarkers: salivary cortisol, interleukin-10 production, and a urine lactulose/mannitol ratio. Questionnaires: IBSQOL, CSFBD, and symptom diary for GI symptoms. |
| Jarrett, Han, et al. (2016) | USA | Non-RCT | Primary | 91 | IBS  (54) | Yes  (37) | III | HRV | Holter electrocardiography | 12 hours | Daily GI symptom diary for 4 weeks.  Thermal pain sensitivity test.  Water load symptom provocation (WLSP) test. |
| Polster, et al. (2018) | Sweden | Non-RCT | Secondary | 197 | IBS  (158) | Yes  (39) | III | HRV | Holter electrocardiography | 24 hours | Questionnaires: IBS-SSS, GSRS-IBS, PHQ-12, HADS. 2-week stool form diary. |
| Nakata, et al. (2022) | Japan | Non-RCT | Secondary (multicenter) | 20 | IBS  (6) | Yes  (14) | IV | HRV | T-shirt wearable device | 24 hours | Smartphone application software for recording real-time life events (e.g., abdominal symptoms, defecation, eating, sleeping)  Questionnaires: IBS-SSS, GSRS, HADS, SF-8. |
| Dal, et al. (2014) | Turkey | Non-RCT | Unknown | 126 | FD  (64) | Yes  (62) | III | HRV | Holter electrocardiography | 24 hours | Transthoracic echocardiography, abdominal ultrasonography, ECG, biochemical analysis, upper gastrointestinal endoscopy. |
| Ochi, et al. (2013) | Japan | Non-RCT | Secondary | 45 | FD  (45) | No  ( - ) | III | HRV | Holter electrocardiography | 24 hours | Radionuclide examinations, endoscopy, H. pylori infection assessment, and histological examinations of biopsies. |
| Lorena, et al. (2002) | Brazil | Non-RCT | Secondary | 35 | FD  (23) | Yes  (12) | - | HRV | Holter electrocardiography | 24 hours | Five cardiovascular reflex tests, including heart rate responses to Valsalva maneuver, standing up, and deep breathing, and blood pressure responses to standing up and sustained handgrip. |
| Tominaga et al. (2016) | Japan | Non-RCT | Tertiary | 45 | FD  (45) | No  ( - ) | II | HRV | Active Tracer™ | 24 hours | Examined HRV at baseline, and after lunch, cold pressor and mental arithmetic tests, and after using tofisopam. Questionnaires: GSRS, SDS, Zung Self-rating Depression Scale, STAI. |
| Shapiro, et al. (2021) | USA | Non-RCT | Primary | 1540 | Functional constipation (1540) | No  ( - ) | - | HRV, Sleep | Fitbit® | 16 weeks | Daily and monthly surveys to assess constipation symptom severity and medication usage. |
| Buchanan, et al. (2014) | USA | Non-RCT | Primary | 24 | IBS  (24) | No  ( - ) | II | Sleep | Wrist-mounted actigraphy | 7 days | Daily sleep and IBS symptom diaries for one menstrual cycle. Questionnaires: PSQI questionnaire, PSG. |
| Patel, et al. (2016) | USA | Non-RCT | Tertiary | 50 | IBS  (24) | Yes  (26) | III | Sleep | Wrist-mounted actigraphy | 7 days | Daily bowel pain logs. Questionnaires: GSRS-IBS, PHQ-15, BDI, BAI, VSI, IBS-QOL, WPAI-IBS, SF-36, PSQI. |
| Rotem, et al. (2003) | Israel | Non-RCT | Secondary | 38 | IBS  (18) | Yes  (20) | I | Sleep | Wrist-mounted actigraphy | 4 nights | PSG. Questionnaires: IBS severity, IBS-QOL, SCL-90R, FBDSI, 1-month sleep history questionnaire, Epworth Sleepiness Scale. |
| Topan, et al. (2024) | UK | Non-RCT | Primary and Tertiary | 80 | IBS  (80) | No  ( - ) | IV | Sleep | Wrist-mounted actigraphy | 7 days | 7 days ESM measurements; 7 days end-of-day diary; end-of-week questionnaires on day 7. |
| Du, et al. (2023) | China | RCT | Secondary | 107 | FD  (107) | No  ( - ) | - | Sleep | Sleepthing™ | Unknown | Experimental group: eszopiclone 3mg, eszopiclone 3mg + estazolam 1 mg, eszopiclone 3mg + estazolam 2 mg for patients with mild, moderate, and severe insomnia, resp. Control group: 1, 2, or 3 tablets of vitamin B complex for patients with mild, moderate, and severe insomnia, resp. Pain scores, sleep parameters, and serum levels of inflammatory mediators were compared before and after treatment. |
| Ono, et al. (2008) | Japan | Non-RCT | Primary | 20 | Functional constipation (10) | Yes  (10) | II | Sleep | Wrist-mounted actigraphy | 7 days | Sleep-Health Risk Index, self-reported questionnaires on bowel habits and sleep health, and fecal collection for 14 days. |
| Orr, et al. (1997) | USA | Non-RCT | Primary | 20 | IBS  (10) | Yes  (10) | Unknown | Gastric myoelectric activity | Ambulatory electrogastrogram | One night | EOG and EMG. |
| Hocke, et al. (2001) | Germany | Non-RCT | Unknown | 54 | FD  (20) | Yes  (10) | - | Gastric myoelectric activity | Ambulatory electrogastrogram | 24 hours | None. |
| Riezzo, et al. (2001) | Italy | Non-RCT | Secondary | 51 | FD  (22) | Yes  (29) | - | Gastric myoelectric activity | Ambulatory electrogastrogram | 7 hours | Symptom questionnaire, endoscopy with biopsy, ultrasound, H. Pylori antibodies, biochemic measurements of gastrointestinal hormones. |
| Parkman, et al. (1997) | USA | Non-RCT | Secondary | 72 | FD  (72) | No  ( - ) | - | Gastric myoelectric activity | Ambulatory electrogastrogram | 2 hours | Gastric scintigraphy. Symptom questionnaire. |
| Pfaffenbach, et al. (1997) | Germany | Non-RCT | Unknown | 45 | FD  (25) | Yes  (20) | - | Gastric myoelectric activity | Ambulatory electrogastrogram | 2 hours | Endoscopy with biopsy for H. Pylori infection assessment. |
| Pfaffenbach, et al. (1998) | Germany | Non-RCT | Unknown | 175 | FD  (25) | Yes  (40) | - | Gastric myoelectric activity | Ambulatory electrogastrogram | 1 hour | Symptom questionnaire, endoscopy with biopsy, H. pylori assessment. |
| Miyaji, et al. (1999) | Japan | Non-RCT | Primary | 56 | FD  (46) | Yes  (10) | - | Gastric myoelectric activity | Ambulatory electrogastrogram | 3 hours | H. Pylori assessment, Gastric emptying test. Questionnaires: Glasgow Dyspepsia Severity Score. |
| Zhang, et al. (2015) | China | RCT | Secondary | 635 | FD  (635) | No  ( - ) | III | Gastric myoelectric activity | Ambulatory electrogastrogram | 1 hour | Symptom scores, SF-36, plasma motilin by radioimmunoassay, gastric emptying by B-sonography. |
| Zhao, et al. (2010) | China | Non-RCT | Secondary | 29 | Functional vomiting  (19) | Yes  (10) | III | Gastric myoelectric activity | Ambulatory electrogastrogram | 1 hour and 30 minutes | Perfusion nutrition load test. Intragastric pressure. Questionnaires: SAS/SDS, EPQ. |
| Gharibans, et al. (2022) | New Zealand | Non-RCT | Secondary | 86 | CNVS  (43) | Yes  (43) | IV | Gastric myoelectric activity | Ambulatory electrogastrogram (Gastric Alimetry) | 4 hours and 40 minutes | Questionnaires: PAGI-SYM, GCSI, PAGI-QoL, VAS scales, STAI-SF, PHQ-2. |
| Schamberg, et al. (2023) | New Zealand | Non-RCT | Secondary (multicenter) | 178 | CNVS  (50) | Yes  (110) | - | Gastric myoelectric activity | Ambulatory electrogastrogram (Gastric Alimetry) | 4 hours and 40 minutes | EGG, Symptom scores logged in the Gastric Alimetry app. |
| Lacy, et al. (2024) | USA | Non-RCT | Tertiary | 22 | Chronic gastroduodenal symptoms  (22) | No  ( - ) | IV | Gastric myoelectric activity | Wireless motility patches | 6 days | Gastric scintigraphy. Questionnaires: PAGI-SYM, Nepean Dyspepsia Index, Mayo Bloating Questionnaire, NRS for abdominal pain, VAS for nausea. |
| Law, et al. (2024) | New Zealand | Non-RCT | Secondary (multicenter) | 28 | Chronic gastroduodenal symptoms  (14) | Yes  (14) | IV | Gastric myoelectric activity | Ambulatory electrogastrogram (Gastric Alimetry) | 4 hours and 40 minutes | Questionnaires: PAGI-SYM, GCSI, PAGI-QoL, PHQ-2. |
| Wang, et al. (2023) | Australia | Non-RCT | Secondary | 75 | Chronic gastroduodenal symptoms  (75) | No  ( - ) | IV | Gastric myoelectric activity | Ambulatory electrogastrogram (Gastric Alimetry) | 4 hours and 40 minutes | Scintigraphic GET. Questionnaires: PAGI-SYM, GCSI, PAGI-QoL, PHQ-2, STAI. |

*Recruitment method: primary, secondary or tertiary care.

BAI, Beck Anxiety Inventory; BDI, Beck Depression Inventory; BDQ, Bowel Disease Questionnaire; cf-PWV, Carotid-femoral Pulse Wave Velocity; CIMT, Carotid Intima-Media Thickness measurement; CNVS, Chronic Nausea and Vomiting Syndrome; CSFBD, Cognitive Scale for Functional Bowel Disorders; CSM, Comprehensive Self-Management intervention; DGBI, Disorder of Gut-Brain Interaction; DIS, Diagnostic Interview Schedule; ECG, electrocardiography; EGG, Electrogastrogram; EMG, electromyogram; EOG, electro-oculogram; EPQ, Eysenck personality questionnaire; ESM, Experience Sampling Method; FBDSI, Functional Bowel Disorder Severity Index; FD, Functional Dyspepsia; GCSI, Gastroparesis Cardinal Symptom Index; GET, Gastric emptying time; GSRS, Gastrointestinal Symptom Rating Scale; HADS, Hospital Anxiety and Depression Scale; HC, Healthy controls; H. Pylori, Helicobacter Pylori; HRV, Heart Rate Variability; IBS, Irritable Bowel Syndrome; IBS-SSS, IBS Symptom Severity Scale; IBSQOL, IBS Quality Of Life; LH, Luteinizing hormone; NRS, Numeric Rating Scale; PAGI-SYM, Patient Assessment of Upper Gastrointestinal Symptom Severity Index; PAGI-QoL, Patient-Assessment of Upper Gastrointestinal Disorders Quality of Life; PHQ-2, Patient Health Questionnaire-2; PHQ-12, Patient Health Questionnaire-12; PHQ-15, Patient Health Questionnaire-15; PSG, Polysomnography; PSQI, Pittsburgh Sleep Quality Index; RCT, Randomized Controlled Trial; SAS/SDS, Zung self-rating anxiety and depression scale; SCL-90, Symptom Checklist-90R; SDS, Zung Self-rating Depression Scale; SF-8, Short-Form health survey-8; SF-36, Short-Form health survey-36; STAI, State-Trait Anxiety Inventory; STAI-SF, State Trait Anxiety Inventory Short Form; USA, United States of America; VAS, Visual Analogue Scales; VSI, Visceral Sensitivity Index; WLSP, Water Load Symptom Provocation test; WPAI-IBS, Work Productivity and Activity Impairment Questionnaire IBS.

- 1. **Patient characteristics**

**Table 2: Patient Characteristics of Included Studies (n = 36)**

| **Characteristic** | **Total** | **DGBI** | **HC** | **P-value** |
| --- | --- | --- | --- | --- |
| Age, years | 40.77 ± 9.36 | 42.03 ± 9.62 | 38.73 ± 8.78 | 0.199 |
| Gender (female), n (%) | 3487 (73.5%) | 2929 (73.5%) | 558 (73.8%) | 0.887 |
| BMI, kg/m^2^ | 25.02 ± 2.34 | 25.19 ± 2.42 | 24.76 ± 2.32 | 0.663 |

A P-value < 0.05 is considered statistically significant. BMI, body mass index; DGBI, disorder of gut-brain interaction; HC, healthy controls.

**Table 3: Detailed overview of Patient Characteristics per Study.**

| **Study** | **Disease** | **Groups** | **Population analyzed ([n])** | **Gender**  **(M/F)** | **Age**  **(Years,**  **(mean ± SD)** | **BMI**  **(kg/m^2^,**  **mean ± SD)** |
| --- | --- | --- | --- | --- | --- | --- |
| Akhan, et al. (2023) | IBS | IBS-D | 25 | 8 / 17 | 38.7 ± 10.5 | 27.2 ±3.5 |
|  |  | IBS-C | 25 | 7 / 18 | 43.0 ± 10.6 | 26.3 ± 4.0 |
|  |  | HC | 50 | 18 / 32 | 39.8 ± 14.2 | 27.5 ± 3.2 |
| Cain, et al. (2006) | IBS | IBS | 165 | 0 / 165 | 32.9 ± 7.7 | 24.8 ± 5.7 |
|  |  | HC | 50 | 0 / 50 | 32.2 ± 7.6 | 24.5 ± 5.2 |
| Chen, et al. (2024) | IBS | IBS (age <46) | 57 | 0 / 57 | 29.7 ± 7.3 | 25.5 ± 6.1 |
|  |  | IBS (age >46) | 32 | 0 / 32 | 57.1 ± 6.7 | 26.4 ± 6.2 |
| Durakoğlugil, et al. (2014) | IBS | IBS | 30 | 0 / 30 | 45.0 ± 12.0 | 29.4 ± 5.9 |
|  |  | HC | 30 | 0 / 30 | 47.0 ± 10.0 | 29.7 ± 5.9 |
| Heitkemper, et al. (1998) | IBS | IBS | 25 | 0 / 25 | 32.6 ± 8.0 | Unknown |
|  |  | HC | 15 | 0 / 15 | 32.5 ± 8.6 |  |
| Heitkemper, et al. (2001) | IBS | IBS | 103 | 0 / 103 | 32.6 ± 8.1 | 23.9 ± 4.8 |
|  |  | HC | 49 | 0 / 49 | 32.2 ± 7.7 | 24.6 ± 5.2 |
| Jarrett, et al. (2003) | IBS | IBS | 163 | 0 / 209 | 33.0 ± 8.0 | Unknown |
|  |  | HC | 46 |  |  |  |
| Jarrett, Cain, et al. (2016) | IBS | CSM | 41 | 4 / 37 | 40.5 ± 14.6 | Unknown |
|  |  | Usual care | 44 | 6 / 38 | 37.9 ± 15.9 |  |
| Jarrett, Han, et al. (2016) | IBS | IBS | 54 | 0 / 54 | 28.4 ± 6.7 | Unknown |
|  |  | HC | 37 | 0 / 37 | 28.6 ± 6.8 |  |
| Polster, et al. (2018) | IBS | IBS | 158 | 45 / 113 | 35.0 | 23.4 ± 3.9 |
|  |  | HC | 39 | 17 / 22 | 29.0 | 22.8 ± 3.3 |
| Nakata, et al. (2022) | IBS | IBS | 6 | 3 / 3 | 50.5 | 21.4 |
|  |  | HC | 14 | 7 / 7 | 51.0 | 22.3 |
| Dal, et al. (2014) | FD | FD | 64 | 15 / 49 | 40.2 ± 7.4 | Unknown |
|  |  | HC | 62 | 17 / 45 | 38.1 ± 10.9 |  |
| Ochi, et al. (2013) | FD | PDS | 24 | 23 / 22 | 54.2 | Unknown |
|  |  | EPS | 21 |  |  |  |
| Lorena, et al. (2002) | FD | FD | 23 | 6 / 17 | 38.0 ± 7.0 | Unknown |
|  |  | HC | 12 | 5 / 7 | 34.0 ± 4.0 |  |
| Tominaga, et al. (2016) | FD | EPS | 24 | 10 / 14 | 53.1 ± 2.1 | Unknown |
|  |  | PDS | 21 | 10 / 11 | 52.1 ± 3.3 |  |
| Shapiro, et al. (2021) | Functional constipation | Functional constipation | 1540 | 419 / 1121 | 36.6 ± 10.0 | 29.7 ± 6.8 |
| Buchanan, et al. (2014) | IBS | IBS | 24 | 0 / 24 | 31.9 ± 8.1 | Unknown |
| Patel, et al. (2016) | IBS | IBS | 24 | 6 / 18 | 44.0 | 27.7 ± 1.1 |
|  |  | HC | 26 | 7 / 19 | 45.0 | 25.8 ± 1.4 |
| Rotem, et al. (2003) | IBS | IBS | 18 | 5 / 13 | 43.1 ± 12.6 | Unknown |
|  |  | HC | 20 | 9 / 11 | 43.4 ± 10.0 |  |
| Topan, et al. (2024) | IBS | IBS | 80 | 9 / 71 | 37.0 | 23 |
| Du, et al. (2023) | FD | Experimental group | 56 | 27/ 29 | 61.6 ± 10.2 | Unknown |
|  |  | Control group | 51 | 20 / 31 | 59.1 ± 9.1 |  |
| Ono, et al. (2008) | Functional constipation | Functional constipation | 10 | 0 / 10 | 49.2 ± 2.9 | 22.7 ± 3.9 |
|  |  | HC | 10 | 0 / 10 | 50.5 ± 4.1 | 22.9 ± 2.4 |
| Orr, et al. (1997) | IBS | IBS | 10 | 0 / 10 | 38.6 | Unknown |
|  |  | HC | 10 | 0 / 10 | Unknown |  |
| Hocke, et al. (2001) | FD | Dyspepsia | 20 | 6 / 14 | 41.0 ± 16.0 | Unknown |
|  |  | Systemic sclerosis | 10 | 0 / 10 | 46.0 ± 13.0 |  |
|  |  | IBS | 7 | 2 / 5 | 42.0 ± 12 |  |
|  |  | Delayed gastric emptying | 7 | 4 / 3 | 57.0 ± 26.0 |  |
|  |  | HC | 10 | 5 / 5 | 33.0 ± 9.0 |  |
| Riezzo, et al. (2001) | FD | FD | 22 | 7 / 15 | 37.7 ± 9.4 | Unknown |
|  |  | HC | 29 | 11 / 18 | 35.2 ± 8.6 |  |
| Parkman, et al. (1997) | FD | FD | 72 | 12 / 60 | 40.4 ± 15.5 | Unknown |
| Pfaffenbach, et al. (1997) | FD | FD | 25 | 8 / 17 | Unknown | Unknown |
|  |  | HC | 20 | 10 / 10 | Unknown |  |
| Pfaffenbach, et al. (1998) | FD | FD | 25 | 8 / 17 | 58.0 | Unknown |
|  |  | Dyspepsia in DM type II | 27 | 14 / 13 | 67.0 |  |
|  |  | Dyspepsia in hyperthyroidism | 23 | 2 / 21 | 69.0 |  |
|  |  | Dyspepsia in progressive systemic scleroderma | 20 | 2 / 18 | 57.0 |  |
|  |  | Dyspepsia in chronic alcoholism | 20 | 16 / 4 | 44.0 |  |
|  |  | Dyspepsia in gastric ulcer | 10 | 4 / 6 | 62.5 |  |
|  |  | Dyspepsia in gastric cancer | 10 | 5 / 5 | 65.0 |  |
|  |  | HC | 40 | 20 / 20 | 50.0 |  |
| Miyaji, et al. (1999) | FD | FD | 46 | 20 / 26 | 65.3 | Unknown |
|  |  | HC | 10 | 5 / 5 | 60.0 |  |
| Zhang, et al. (2015) | FD | Combined DG & EA | 158 | 82 / 76 | 41.9 ± 12.6 | Unknown |
|  |  | EA | 159 | 81 / 78 | 42.6 ± 11.9 |  |
|  |  | DG | 158 | 80 / 78 | 42.5 ± 10.2 |  |
|  |  | Control | 160 | 81 / 79 | 41.8 ± 12.2 |  |
| Zhao, et al. (2010) | Functional vomiting | Functional vomiting | 19 | 4 / 15 | 25.8 ± 8.5 | Unknown |
|  |  | HC | 10 | 2 / 8 | 30.2 ± 7.7 |  |
| Gharibans, et al. (2022) | CNVS | CNVS | 43 | 8 / 35 | 36.1 ± 13.2 | 24.9 ± 4.6 |
|  |  | HC | 43 | 12 / 31 | 36.1 ± 14.5 | 23.7 ± 4.0 |
| Schamberg, et al. (2023) | CNVS | CNVS | 50 | 11 / 39 | 36.0 | 24.0 |
|  |  | T1DM | 39 | 15 / 24 | 49.0 | 25.1 |
|  |  | HC | 110 | 49 / 61 | 32.0 | 23.8 |
| Lacy, et al. (2024) | Chronic gastroduodenal symptoms | Normal gastric emptying | 19 | 3 / 16 | 45.0 | 25.9 |
|  |  | Delayed gastric emptying | 3 | 0 / 3 | 32.3 | 28.6 |
| Law, et al. (2024) | Chronic gastroduodenal symptoms | Chronic gastroduodenal symptoms | 14 | 6 / 8 | 48.0 | Unknown |
|  |  | HC | 14 | 4 / 10 | 33.5 |  |
| Wang, et al. (2023) | Chronic gastroduodenal symptoms | Chronic gastroduodenal symptoms | 75 | 17 / 58 | 43.0 | 24.0 |

BMI, Body Mass Index; CNVS, Chronic Nausea and Vomiting Syndrome; CSM, Comprehensive Self-Management intervention; DG, Dalitong Granule; EA, Electroacupuncture; EPS, Epigastric Pain Syndrome; F, Female; FD, Functional Dyspepsia; HC, Healthy controls; IBS, Irritable Bowel Syndrome; IBS-C, IBS constipation; IBS-D, IBS diarrhea; M, Male; PDS, Postprandial Distress Syndrome; T1DM, type 1 diabetes mellitus.

- 1. **Risk of bias**

Three included articles (11-13) were RCTs and were assessed as having “some concerns” regarding the risk of bias. This classification primarily stemmed from the lack of detailed descriptions of blinding methods (12, 13), absence of intention-to-treat analysis (11-13), use of multiple analytical approaches (11), and unavailable trial protocols (11-13), increasing the risk of selective reporting. Among these, only Jarrett et al. (11) specified that data were analyzed by a blinded research nurse. Despite these limitations, all RCT’s implemented appropriate randomization methods with no baseline differences between groups. Autonomic parameters were measured consistently using appropriate techniques, with outcome data available for most participants and reasons for missing data clearly documented.

All other articles were observational studies and classified as having a “low” (n = 3), “moderate” (n = 25), or “serious” (n = 5) risk of bias. Many studies were small, limiting their power to control for important confounders. While most studies appropriately accounted for some relevant confounders (14-33), such as age, gender, BMI, medication use, and menstrual cycle, others lacked information or adjustment for these factors (34-43). Participant selection was generally based on well-established criteria, such as the Rome criteria, though some studies failed to apply these criteria (23, 35, 42, 44). Outcome data were available for most participants, with missing data well-documented and comparable across groups in many studies. Nonetheless, a subset of studies provided no information on missing data or their handling (14, 17, 20, 22, 23, 25, 29, 35-38, 41, 43), raising concerns about bias. Blinding was implemented in only a few studies (17, 19, 22, 24, 28, 32, 34), typically at the assessor or analyst level. Nevertheless, the autonomic parameters measured were objective, reducing the potential impact of non-blinding. Most studies employed adequate statistical plans and corrected for multiple comparisons (20, 32, 33, 45). However, selective reporting was evident in some cases, where not all data or comparisons were presented (14, 18, 23, 25). The five studies (35, 38, 41, 43) classified as having a “serious” risk of bias were notable for their lack of mentioning and control for potential confounders (35, 38, 41-43), poorly defined selection criteria (35, 43), missing information on outcome data (35, 38, 41, 43), absence of blinding methods (35, 38, 43), and inadequate reporting of results (35, 38, 42, 43). Of these studies, only 2 (42, 43) were included in the meta-analyses on gastric myoelectric activity, allowing for a broader understanding of the available evidence despite their limitations.

**Table 4: Overall Risk of Bias per Article**

| **Study** | **Study Design** | **Risk of Bias** |
| --- | --- | --- |
| Akhan, et al. (2023) | Non-RCT | Moderate |
| Cain, et al. (2006) | Non-RCT | Moderate |
| Chen, et al. (2024) | Non-RCT | Moderate |
| Durakoğlugil, et al. (2014) | Non-RCT | Moderate |
| Heitkemper, et al. (1998) | Non-RCT | Moderate |
| Heitkemper, et al. (2001) | Non-RCT | Moderate |
| Jarrett, et al. (2003) | Non-RCT | Moderate |
| Jarrett, Cain, et al. (2016) | RCT | Some concerns |
| Jarrett, Han, et al. (2016) | Non-RCT | Moderate |
| Polster, et al. (2018) | Non-RCT | Moderate |
| Nakata et al. (2022) | Non-RCT | Moderate |
| Dal, et al. (2014) | Non-RCT | Moderate |
| Ochi, et al. (2013) | Non-RCT | Serious |
| Lorena, et al. (2002) | Non-RCT | Moderate |
| Tominaga, et al. (2016) | Non-RCT | Moderate |
| Shapiro, et al. (2021) | Non-RCT | Moderate |
| Buchanan, et al. (2014) | Non-RCT | Low |
| Patel, et al. (2016) | Non-RCT | Low |
| Rotem, et al. (2003) | Non-RCT | Moderate |
| Topan, et al. (2024) | Non-RCT | Moderate |
| Du, et al. (2023) | RCT | Some concerns |
| Ono, et al. (2008) | Non-RCT | Moderate |
| Orr, et al. (1997) | Non-RCT | Moderate |
| Hocke, et al. (2001) | Non-RCT | Serious |
| Riezzo, et al. (2001) | Non-RCT | Serious |
| Parkman, et al. (1997) | Non-RCT | Serious |
| Pfaffenbach, et al. (1997) | Non-RCT | Moderate |
| Pfaffenbach, et al. (1998) | Non-RCT | Moderate |
| Miyaji, et al. (1999) | Non-RCT | Serious |
| Zhang, et al. (2015) | RCT | Some concerns |
| Zhao, et al. (2010) | Non-RCT | Moderate |
| Gharibans, et al. (2022) | Non-RCT | Moderate |
| Schamberg, et al. (2023) | Non-RCT | Moderate |
| Lacy, et al. (2024) | Non-RCT | Moderate |
| Law, et al. (2024) | Non-RCT | Moderate |
| Wang, et al. (2023) | Non-RCT | Low |

RCT, randomized controlled trial.

**
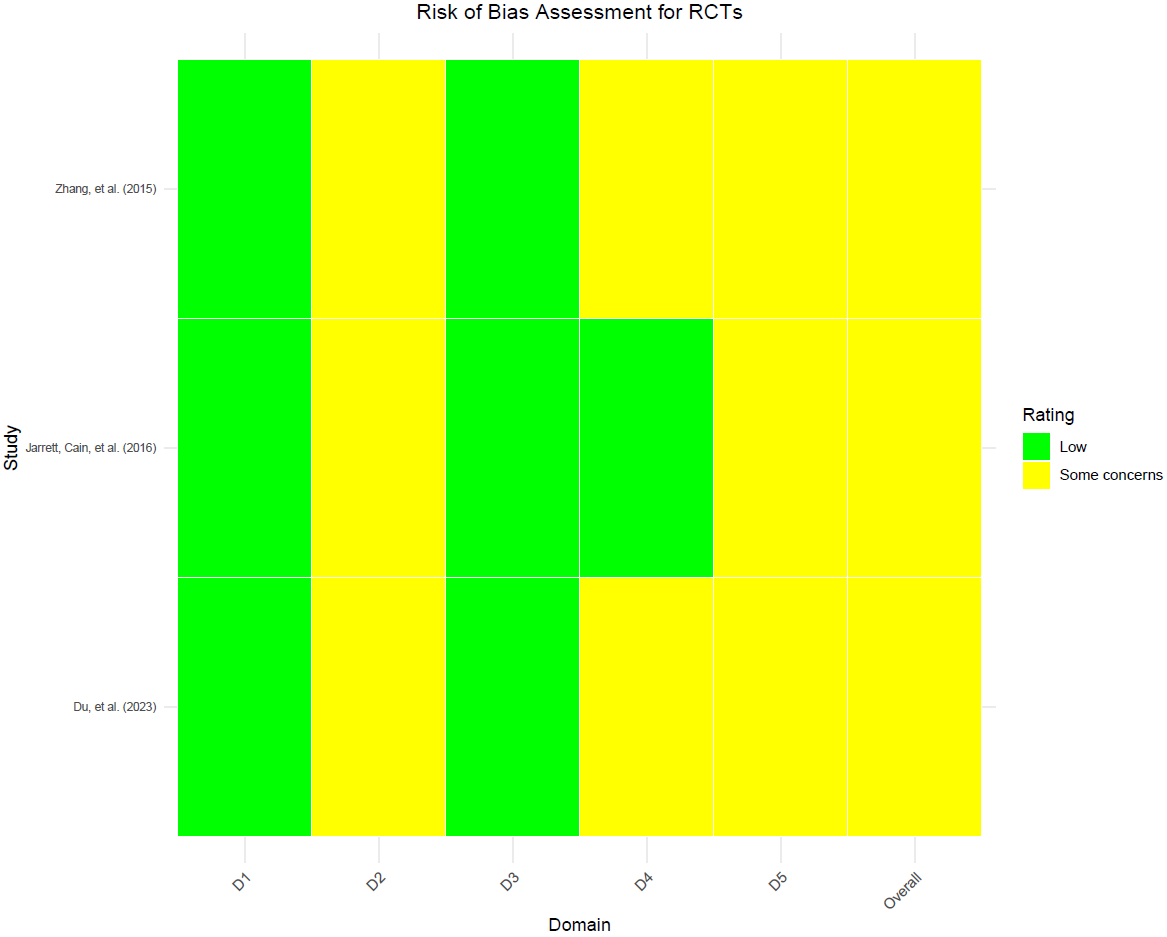
**

**Figure 1. Risk of Bias Assessment for RCTs**

**
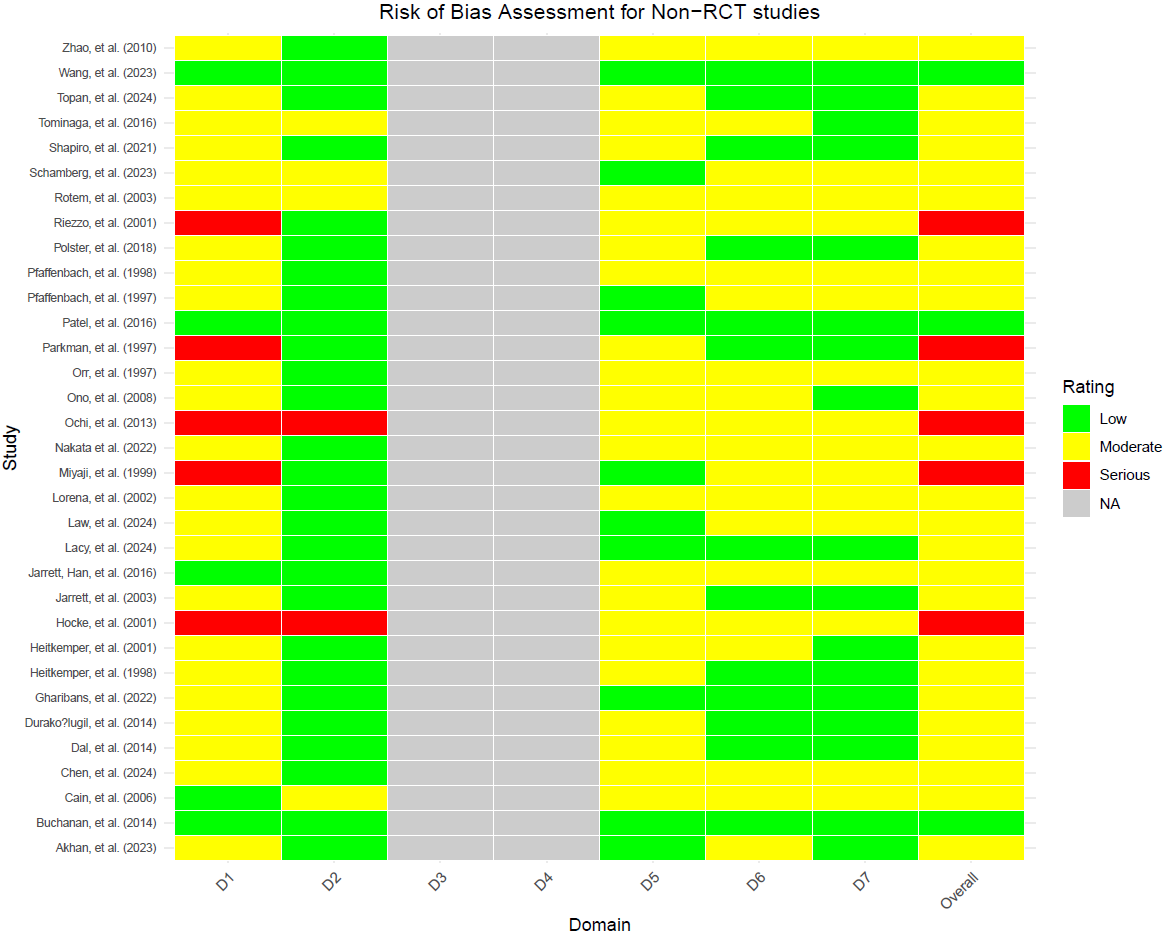
**

**Figure 2. Risk of Bias Assessment for Non-RCT Studies**

- 1. **Heart rate variability**

A total of 16 studies investigated HRV in individuals with DGBI (n=2645), including IBS (n=928, 11 studies), FD (n=177, 4 studies), and functional constipation (n=1540, 1 study). HRV was assessed using Holter electrocardiography (13 studies), a T-shirt wearable device (1 study), Active Tracer™ (1 study), or Fitbit® (1 study). Both time-domain (9 studies) and frequency-domain (13 studies) HRV parameters were reported, including key parameters like the root mean square of the successive differences between RR intervals (RMSSD), percentage of successive RR intervals differing by more than 50 ms (pNN50), standard deviation of NN intervals (SDNN), high frequency (HF) power, low frequency (LF) power, and LF/HF ratio (Table 1). Five studies did not include a healthy control group and employed heterogeneous protocols for HRV assessment in DGBI (Table 2 main document).

Eleven studies were included in the meta-analyses: Seven utilized time-domain and nine used frequency-domain HRV parameters, with five employing both for autonomic assessment (Table 5).

*Time-domain parameters.* The meta-analysis on RMSSD, including seven studies (IBS: n=5, FD: n=2), yielded a moderate negative pooled effect size of –0.503 (SE=0.189, 95%CI [-0.873, -0.132]), indicating lower RMSSD values in the DGBI group compared to HCs (Figure 6 (A)). High heterogeneity was observed (I^2^=82.82%, Q-statistic P<0.001). pNN50 (6 studies: IBS n=4, FD n=2) was also lower in DGBI than in HCs (SMD= -0.430, SE=0.176, 95%CI [-0.775, -0.085], I^2^=78.47%, Q-statistic P<0.001) (Figure 6 (B)). Exploratory analysis on SDNN (4 studies: IBS n=2, FD n=2) showed a negative moderate pooled effect size of –0.398 (SE=0.308, 95%CI [-1.001, 0.206], I^2^=85.85%, Q-statistic P<0.001) (Figure 6 (C)).

*Frequency-domain parameters.* The meta-analysis on HF power (8 studies: IBS n=7, FD n=1) and LF power (6 studies: IBS n=5, FD n=1) showed no differences between DGBI and HCs, with negative pooled effect sizes of –0.050 (SE=0.190, 95%CI [-0.422, 0.321], I^2^=79.78%, Q-statistic P<0.001) (Figure 10 (A)) and –0.230 (SE = 0.165, 95% CI [-0.553, -0.093], I^2^=65,78%, Q-statistic P=0.012) (Figure 10 (B)), respectively. The LF/HF ratio (8 studies: IBS n=6, FD n=2) was also not different (SMD=0.017, SE=0.079, 95%CI [-0.137, 0.172], I^2^=0.00%, Q-statistic P=0.692) (Figure 10 (C)). See Table 3 (main document) for an overview of the results.

**Table 5: Studies Assessing HRV as an Outcome Measure with Comparison between DGBIs and Healthy Controls.**

| **Outcome parameter** | **Studies reporting this parameter in IBS** | **Studies reporting this parameter in FD** |
| --- | --- | --- |
| **Time-domain parameters** | | |
| RMSSD | Akhan (2023), Durakoğlugil (2014), Heitkemper (2001), Jarrett (2003), Polster (2018). | Dal (2014), Lorena (2002). |
| SDNN | Durakoğlugil (2014), Polster (2018). | Dal (2014), Lorena (2002). |
| SDNN Index | Akhan (2023), Durakoğlugil (2014), Polster (2018). | - |
| pNN50 | Akhan (2023), Heitkemper (2001), Jarrett (2003), Polster (2018). | Dal (2014), Lorena (2002). |
| SDANN | Durakoğlugil (2014), Heitkemper (2001). | - |
| SD-5 min | Heitkemper (2001), Jarrett (2003). | - |
| **Frequency-domain parameters** | | |
| HF | Cain (2006), Durakoğlugil (2014), Heitkemper (1998), Heitkemper (2001), Jarrett (2003), Jarrett & Han (2016), Nakata (2022). | Lorena (2002). |
| LF | Durakoğlugil (2014), Heitkemper (1998), Heitkemper (2001), Jarrett (2003), Jarrett & Han (2016). | Lorena (2002). |
| LF/HF ratio | Cain (2006), Durakoğlugil (2014), Heitkemper (1998), Heitkemper (2001), Jarrett & Han (2016), Nakata (2022). | Dal (2014), Lorena (2002). |
| TP | Durakoğlugil (2014), Heitkemper (2001), Jarrett & Han, (2016), Nakata (2022). | - |

DGBI, Disorder of Gut-Brain Interaction; FD, Functional Dyspepsia; HF, High Frequency; HR, Heart Rate; IBS, Irritable Bowel Syndrome; LF, Low Frequency; LF/HF Ratio, Low Frequency and High Frequency Ratio; pNN50, Percentage of successive RR intervals differing by more than 50 ms; RMSSD, Root Mean Square of the Successive Differences between RR intervals; SDANN, Standard Deviation of the Averages of Normal-to-Normal Intervals; SDNN, Standard Deviation of Normal-to-Normal Intervals; SD-5min, Standard Deviation over 5 minutes; TP, Total Power.

**RMSSD**

**Table 6: Sensitivity Analysis for RMSSD**

| **Study** | **estimate** | **se** | **zval** | **pval** | **ci.lb** | **ci.ub** | **Q** | **Qp** | **Tau2** | **I2** | **H2** |
| --- | --- | --- | --- | --- | --- | --- | --- | --- | --- | --- | --- |
| **Akhan (2023)** | -0.4814 | 0.2188 | -2.2004 | 0.0278 | -0.9101 | -0.0526 | 33.2930 | 0.0000 | 0.2355 | 84.9818 | 6.6586 |
| **Durakoğlugil (2014)** | -0.4594 | 0.2081 | -2.2073 | 0.0273 | -0.8673 | -0.0515 | 32.6185 | 0.0000 | 0.2133 | 84.6713 | 6.5237 |
| **Heitkemper (2001)** | -0.6083 | 0.1955 | -3.1110 | 0.0019 | -0.9915 | -0.2251 | 24.8591 | 0.0001 | 0.1765 | 79.8866 | 4.9718 |
| **Jarrett (2003)** | -0.6080 | 0.1942 | -3.1306 | 0.0017 | -0.9886 | -0.2273 | 24.1439 | 0.0002 | 0.1730 | 79.2909 | 4.8288 |
| **Polster (2018)** | -0.3898 | 0.1755 | -2.2208 | 0.0264 | -0.7338 | -0.0458 | 20.5183 | 0.0010 | 0.1339 | 75.6315 | 4.1037 |
| **Dal (2014)** | -0.5279 | 0.2294 | -2.3014 | 0.0214 | -0.9775 | -0.0783 | 34.9251 | 0.0000 | 0.2619 | 85.6836 | 6.9850 |
| **Lorena (2002)** | -0.4417 | 0.1990 | -2.2202 | 0.0264 | -0.8317 | -0.0518 | 32.1334 | 0.0000 | 0.1985 | 84.4399 | 6.4267 |

This table presents the results of the leave-one-out sensitivity analysis for the root mean square of successive differences between RR intervals (RMSSD). The columns show the study name, effect estimate, standard error (se), z-value (zval), p-value (pval), confidence interval (CI) lower bound (ci.lb) and upper bound (ci.ub), Q-statistic, p-value for Q-statistic (Qp), Tau^2^, I^2^ (percentage of variation across studies), and H^2^ (heterogeneity index).

**
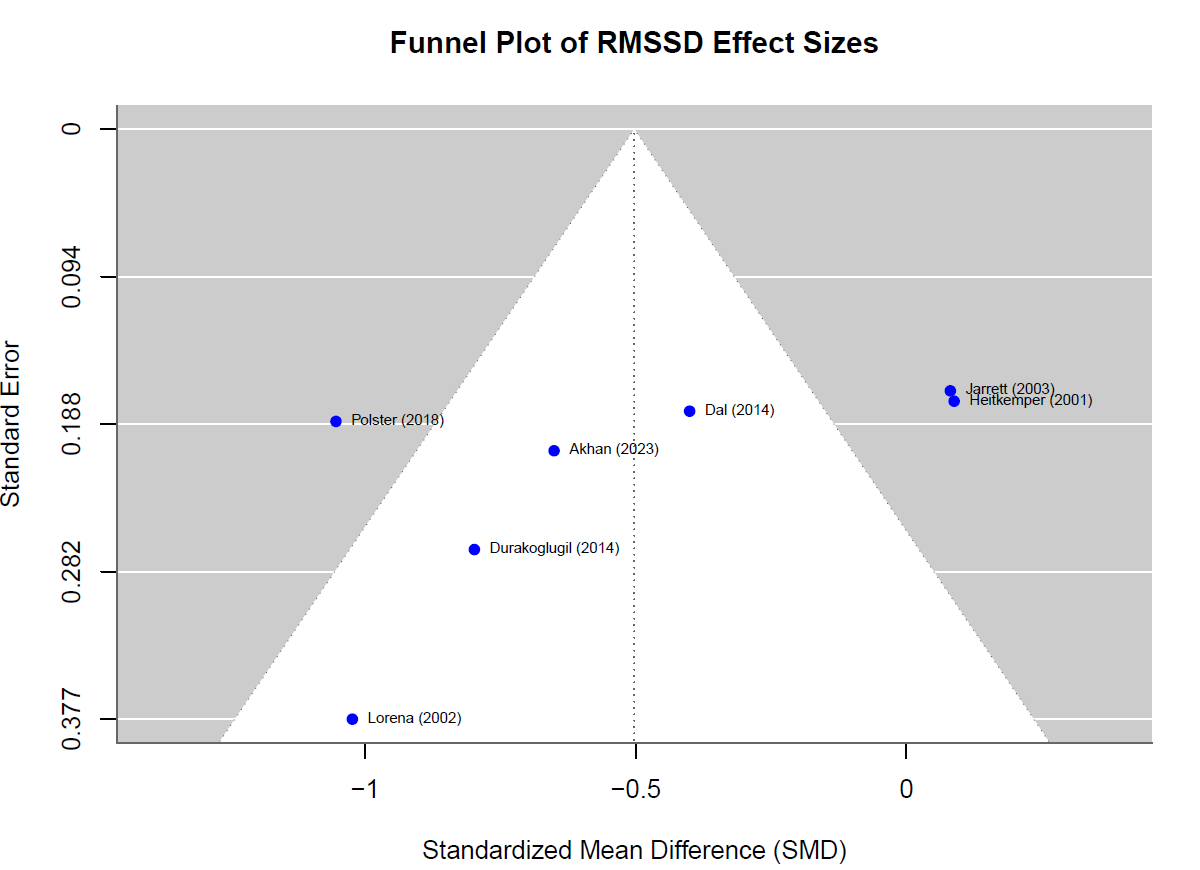
**

**Figure 3. Funnel Plot of RMSSD Effect Sizes**

**pNN50**

**Table 7: Sensitivity Analysis for pNN50**

| **Study** | **estimate** | **se** | **zval** | **pval** | **ci.lb** | **ci.ub** | **Q** | **Qp** | **Tau2** | **I2** | **H2** |
| --- | --- | --- | --- | --- | --- | --- | --- | --- | --- | --- | --- |
| **Akhan (2023)** | -0.4127 | 0.2097 | -1.9687 | 0.0490 | -0.8236 | -0.0018 | 22.0858 | 0.0002 | 0.1727 | 81.8888 | 5.5215 |
| **Heitkemper (2001)** | -0.5363 | 0.1843 | -2.9100 | 0.0036 | -0.8975 | -0.1751 | 15.8621 | 0.0032 | 0.1217 | 74.7827 | 3.9655 |
| **Jarrett (2003)** | -0.5354 | 0.1841 | -2.9076 | 0.0036 | -0.8963 | -0.1745 | 15.5593 | 0.0037 | 0.1208 | 74.2919 | 3.8898 |
| **Polster (2018)** | -0.4074 | 0.2115 | -1.9258 | 0.0541 | -0.8219 | 0.0072 | 21.3754 | 0.0003 | 0.1747 | 81.2869 | 5.3439 |
| **Dal (2014)** | -0.3640 | 0.1931 | -1.8844 | 0.0595 | -0.7425 | 0.0146 | 17.9028 | 0.0013 | 0.1387 | 77.6571 | 4.4757 |
| **Lorena (2002)** | -0.3389 | 0.1737 | -1.9512 | 0.0510 | -0.6793 | 0.0015 | 18.3623 | 0.0010 | 0.1177 | 78.2162 | 4.5906 |

This table presents the results of the leave-one-out sensitivity analysis for the percentage of successive RR intervals differing by more than 50 ms (pNN50). The columns show the study name, effect estimate, standard error (se), z-value (zval), p-value (pval), confidence interval (CI) lower bound (ci.lb) and upper bound (ci.ub), Q-statistic, p-value for Q-statistic (Qp), Tau^2^, I^2^ (percentage of variation across studies), and H^2^ (heterogeneity index).

**
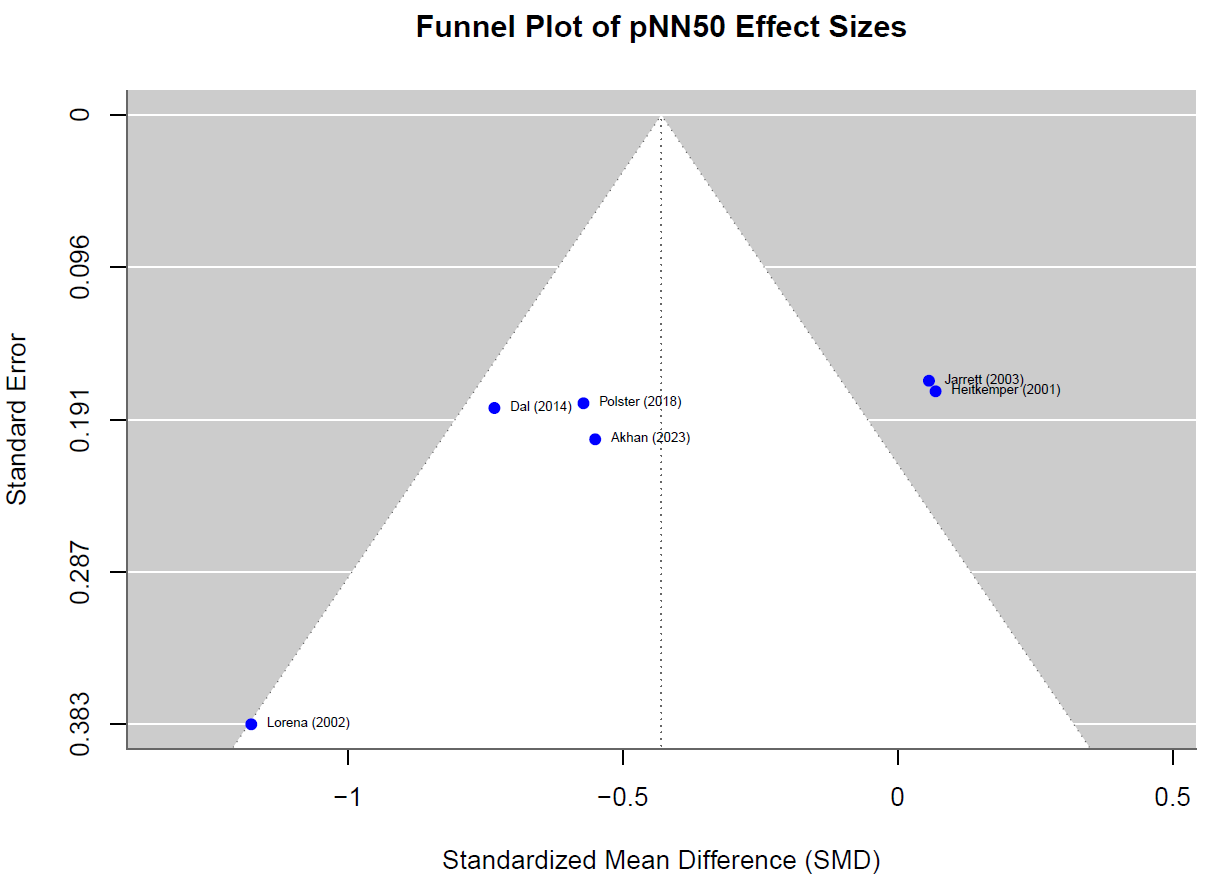
**

**Figure 4. Funnel Plot of pNN50 Effect Sizes**

**SDNN**

**Table 8: Sensitivity Analysis for SDNN**

| **Study** | **estimate** | **se** | **zval** | **pval** | **ci.lb** | **ci.ub** | **Q** | **Qp** | **Tau2** | **I2** | **H2** |
| --- | --- | --- | --- | --- | --- | --- | --- | --- | --- | --- | --- |
| **Durakoğlugil (2014)** | -0.3918 | 0.4227 | -0.9270 | 0.3539 | -1.2202 | 0.4366 | 21.1798 | 0.0000 | 0.4747 | 90.5571 | 10.5899 |
| **Polster (2018)** | -0.0934 | 0.1450 | -0.6445 | 0.5193 | -0.3776 | 0.1907 | 2.1814 | 0.3360 | 0.0059 | 8.3149 | 1.0907 |
| **Dal (2014)** | -0.5832 | 0.3102 | -1.8803 | 0.0601 | -1.1911 | 0.0247 | 8.4478 | 0.0146 | 0.2167 | 76.3252 | 4.2239 |
| **Lorena (2002)** | -0.4763 | 0.3775 | -1.2617 | 0.2070 | -1.2161 | 0.2636 | 20.2684 | 0.0000 | 0.3831 | 90.1324 | 10.1342 |

This table presents the results of the leave-one-out sensitivity analysis for standard deviation of NN intervals (SDNN). The columns show the study name, effect estimate, standard error (se), z-value (zval), p-value (pval), confidence interval (CI) lower bound (ci.lb) and upper bound (ci.ub), Q-statistic, p-value for Q-statistic (Qp), Tau^2^, I^2^ (percentage of variation across studies), and H^2^ (heterogeneity index).

**
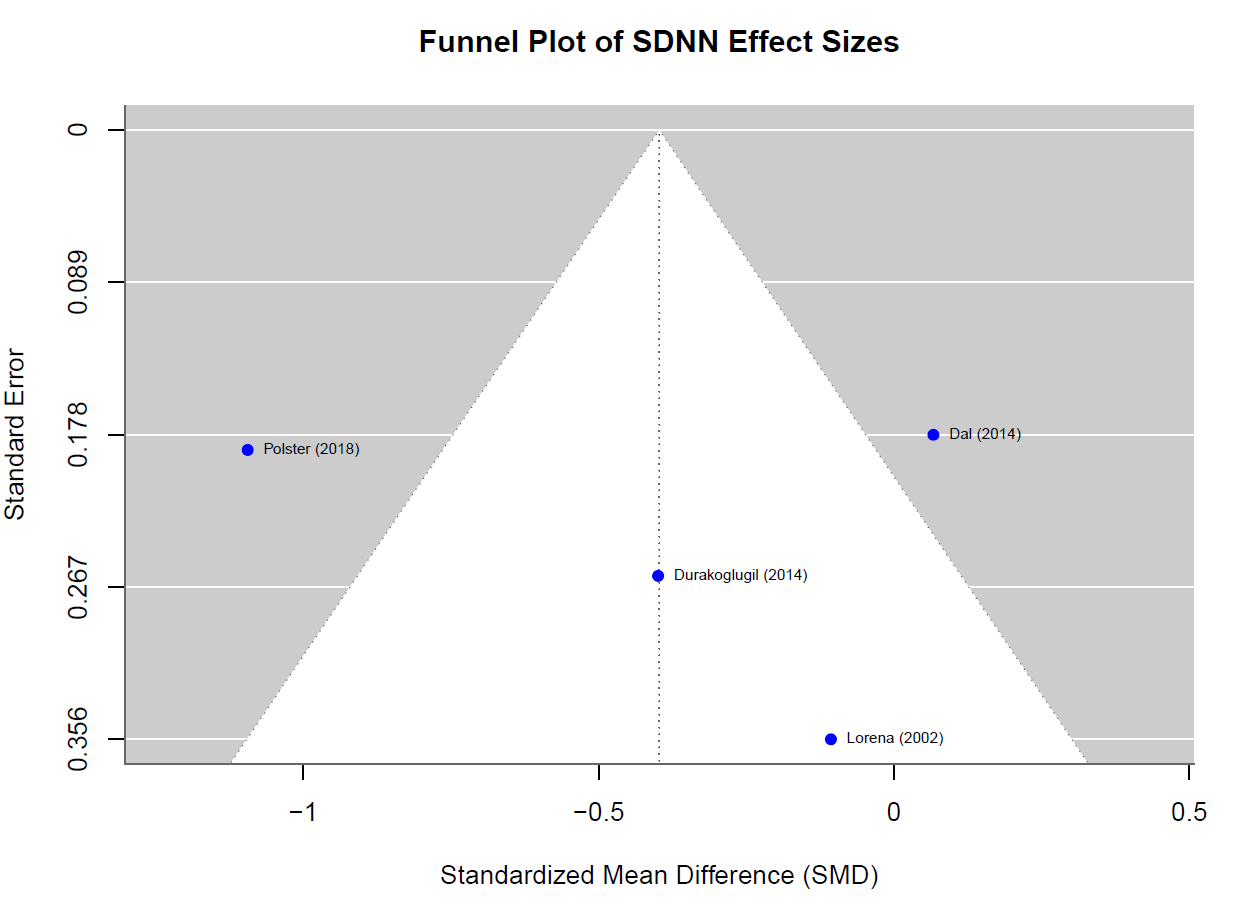
**

**Figure 5. Funnel Plot of SDNN Effect Sizes**

**
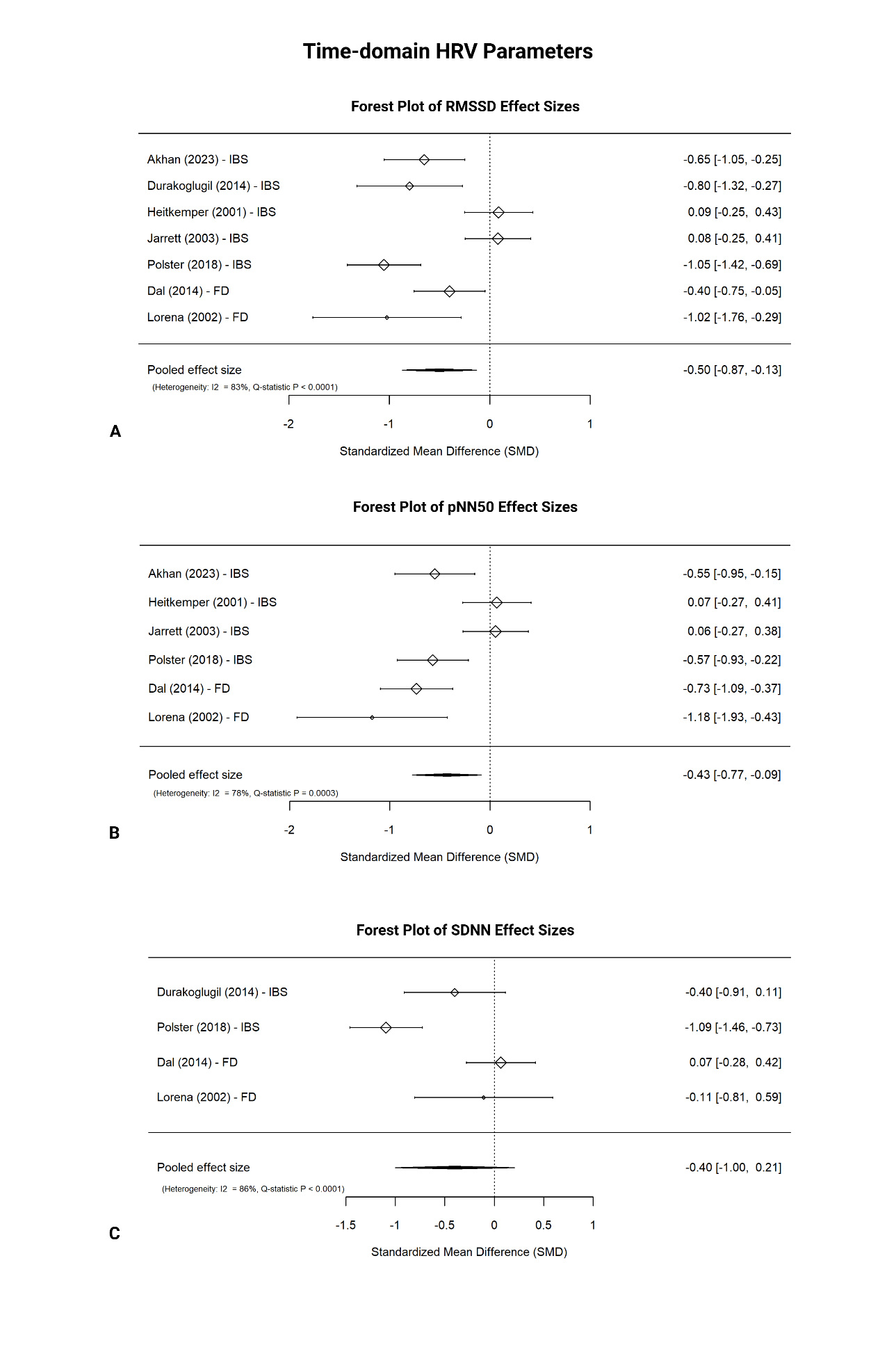
**

**Figure 6. Forest Plots of Time-Domain HRV Parameters**

**HF power**

**Table 9: Sensitivity Analysis for HF**

| **Study** | **estimate** | **se** | **zval** | **pval** | **ci.lb** | **ci.ub** | **Q** | **Qp** | **Tau2** | **I2** | **H2** |
| --- | --- | --- | --- | --- | --- | --- | --- | --- | --- | --- | --- |
| **Cain (2006)** | -0.0237 | 0.2352 | -0.1006 | 0.9199 | -0.4846 | 0.4373 | 34.6093 | 0.0000 | 0.2930 | 82.6636 | 5.7682 |
| **Durakoğlugil (2014)** | 0.0462 | 0.1938 | 0.2382 | 0.8118 | -0.3338 | 0.4261 | 27.6562 | 0.0001 | 0.1841 | 78.3051 | 4.6094 |
| **Heitkemper (1998)** | -0.0674 | 0.2096 | -0.3215 | 0.7479 | -0.4782 | 0.3435 | 34.1763 | 0.0000 | 0.2300 | 82.4440 | 5.6961 |
| **Heitkemper (2001)** | -0.0481 | 0.2295 | -0.2098 | 0.8338 | -0.4980 | 0.4017 | 33.8894 | 0.0000 | 0.2762 | 82.2954 | 5.6482 |
| **Jarrett (2003)** | -0.0600 | 0.2281 | -0.2630 | 0.7926 | -0.5070 | 0.3870 | 32.9209 | 0.0000 | 0.2714 | 81.7745 | 5.4868 |
| **Jarrett, Han (2016)** | 0.0037 | 0.2190 | 0.0168 | 0.9866 | -0.4255 | 0.4328 | 33.3204 | 0.0000 | 0.2478 | 81.9930 | 5.5534 |
| **Nakata (2022)** | -0.1887 | 0.1312 | -1.4384 | 0.1503 | -0.4459 | 0.0684 | 14.7783 | 0.0221 | 0.0673 | 59.3998 | 2.4630 |
| **Lorena (2002)** | 0.0429 | 0.1914 | 0.2242 | 0.8226 | -0.3323 | 0.4182 | 29.1488 | 0.0001 | 0.1854 | 79.4160 | 4.8581 |

This table presents the results of the leave-one-out sensitivity analysis for high frequency (HF). The columns show the study name, effect estimate, standard error (se), z-value (zval), p-value (pval), confidence interval (CI) lower bound (ci.lb) and upper bound (ci.ub), Q-statistic, p-value for Q-statistic (Qp), Tau^2^, I^2^ (percentage of variation across studies), and H^2^ (heterogeneity index).

**
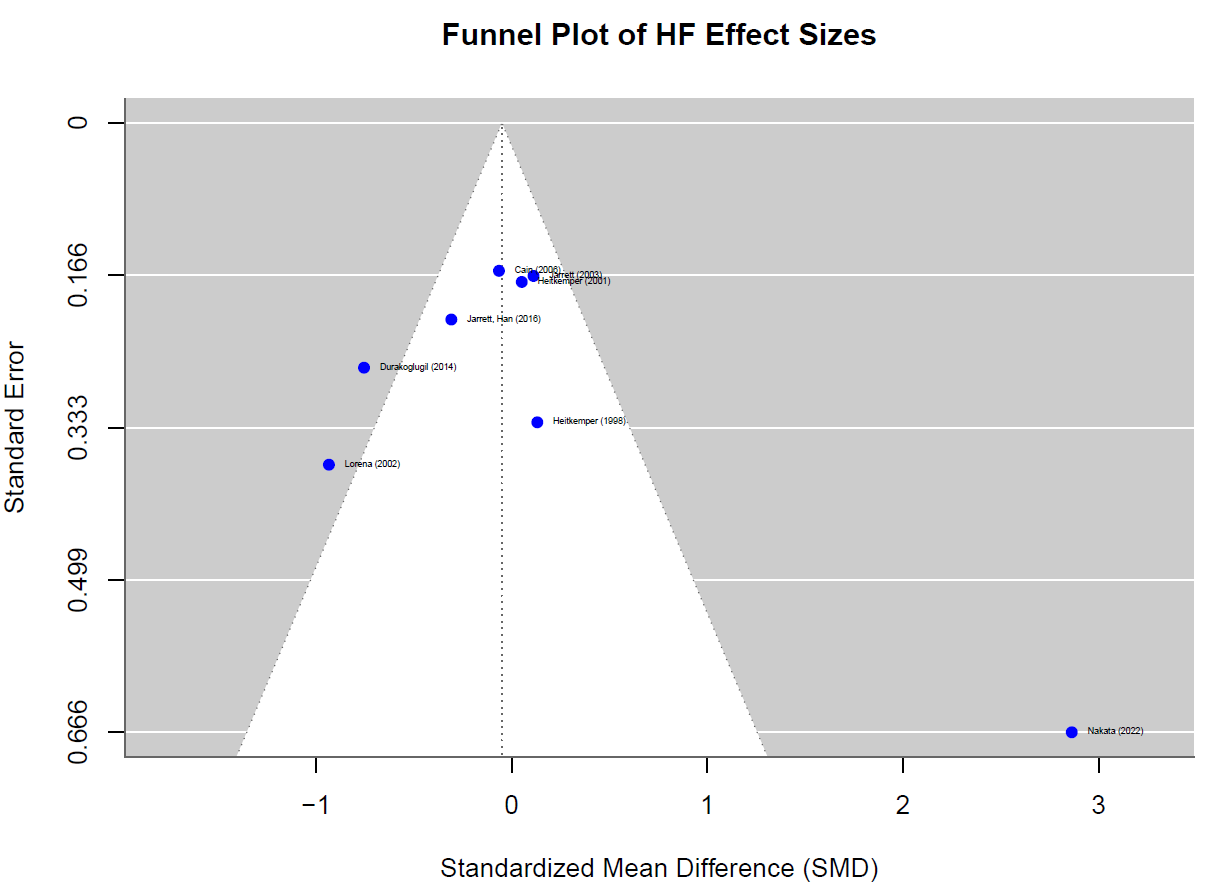
**

**Figure 7. Funnel Plot of HF Effect Sizes**

**LF Power**

**Table 10: Sensitivity Analysis for LF**

| **Study** | **estimate** | **se** | **zval** | **pval** | **ci.lb** | **ci.ub** | **Q** | **Qp** | **Tau2** | **I2** | **H2** |
| --- | --- | --- | --- | --- | --- | --- | --- | --- | --- | --- | --- |
| **Durakoğlugil (2014)** | -0.1156 | 0.1515 | -0.7631 | 0.4454 | -0.4127 | 0.1814 | 8.6378 | 0.0708 | 0.0585 | 53.6920 | 2.1595 |
| **Heitkemper (1998)** | -0.2907 | 0.1857 | -1.5657 | 0.1174 | -0.6547 | 0.0732 | 13.8653 | 0.0077 | 0.1172 | 71.1510 | 3.4663 |
| **Heitkemper (2001)** | -0.3118 | 0.2048 | -1.5226 | 0.1279 | -0.7132 | 0.0896 | 12.9249 | 0.0116 | 0.1387 | 69.0520 | 3.2312 |
| **Jarrett (2003)** | -0.3241 | 0.1939 | -1.6718 | 0.0946 | -0.7041 | 0.0559 | 11.3829 | 0.0226 | 0.1170 | 64.8596 | 2.8457 |
| **Jarrett, Han (2016)** | -0.2236 | 0.2015 | -1.1093 | 0.2673 | -0.6186 | 0.1714 | 13.8657 | 0.0077 | 0.1366 | 71.1518 | 3.4664 |
| **Lorena (2002)** | -0.1348 | 0.1532 | -0.8798 | 0.3789 | -0.4349 | 0.1654 | 9.8045 | 0.0439 | 0.0671 | 59.2024 | 2.4511 |

This table presents the results of the leave-one-out sensitivity analysis for low frequency (LF). The columns show the study name, effect estimate, standard error (se), z-value (zval), p-value (pval), confidence interval (CI) lower bound (ci.lb) and upper bound (ci.ub), Q-statistic, p-value for Q-statistic (Qp), Tau^2^, I^2^ (percentage of variation across studies), and H^2^ (heterogeneity index).

**
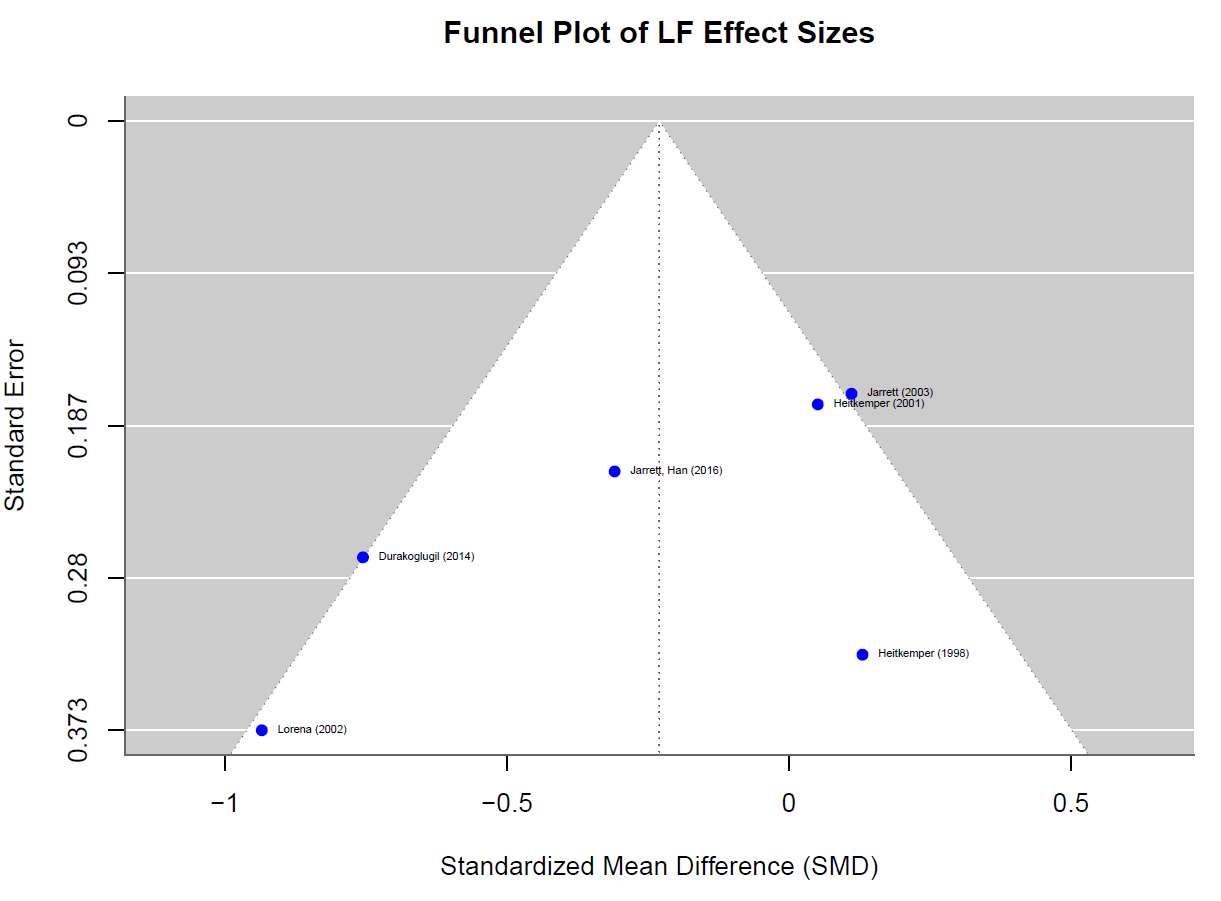
**

**Figure 8. Funnel Plot of LF Effect Sizes**

**LF/HF Ratio**

**Table 11: Sensitivity Analysis for LF/HF**

| **Study** | **estimate** | **se** | **zval** | **pval** | **ci.lb** | **ci.ub** | **Q** | **Qp** | **Tau2** | **I2** | **H2** |
| --- | --- | --- | --- | --- | --- | --- | --- | --- | --- | --- | --- |
| **Cain (2006)** | 0.0171 | 0.0903 | 0.1899 | 0.8494 | -0.1599 | 0.1942 | 4.7399 | 0.5776 | 0.0000 | 0.0000 | 1.0000 |
| **Durakoğlugil (2014)** | -0.0004 | 0.0828 | -0.0053 | 0.9958 | -0.1626 | 0.1618 | 4.2438 | 0.6437 | 0.0000 | 0.0000 | 1.0000 |
| **Heitkemper (1998)** | 0.0325 | 0.0812 | 0.4003 | 0.6890 | -0.1267 | 0.1917 | 4.1357 | 0.6583 | 0.0000 | 0.0000 | 1.0000 |
| **Heitkemper (2001)** | 0.0303 | 0.0885 | 0.3429 | 0.7317 | -0.1431 | 0.2037 | 4.6350 | 0.5914 | 0.0000 | 0.0000 | 1.0000 |
| **Jarrett (2016)** | -0.0002 | 0.0848 | -0.0024 | 0.9981 | -0.1664 | 0.1660 | 4.4265 | 0.6192 | 0.0000 | 0.0000 | 1.0000 |
| **Nakata (2022)** | 0.0397 | 0.0798 | 0.4979 | 0.6185 | -0.1167 | 0.1961 | 1.4651 | 0.9618 | 0.0000 | 0.0000 | 1.0000 |
| **Dal (2014)** | 0.0050 | 0.0879 | 0.0567 | 0.9548 | -0.1673 | 0.1772 | 4.6392 | 0.5909 | 0.0000 | 0.0000 | 1.0000 |
| **Lorena (2002)** | 0.0118 | 0.0808 | 0.1464 | 0.8836 | -0.1466 | 0.1702 | 4.6458 | 0.5900 | 0.0000 | 0.0000 | 1.0000 |

This table presents the results of the leave-one-out sensitivity analysis for low frequency and high frequency ratio (LF/HF ratio). The columns show the study name, effect estimate, standard error (se), z-value (zval), p-value (pval), confidence interval (CI) lower bound (ci.lb) and upper bound (ci.ub), Q-statistic, p-value for Q-statistic (Qp), Tau^2^, I^2^ (percentage of variation across studies), and H^2^ (heterogeneity index).

**
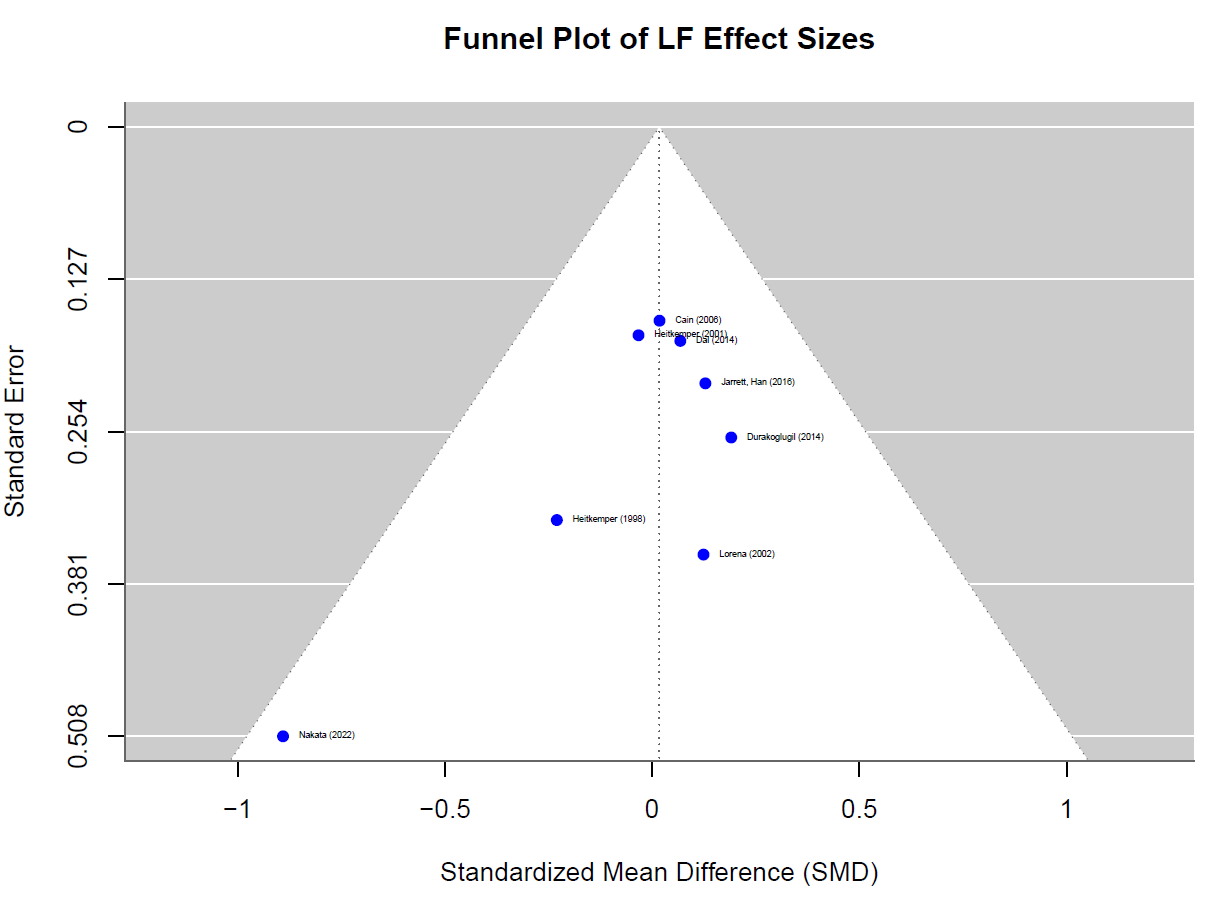
**

**Figure 9. Funnel Plot of LF/HF Ratio Effect Sizes**

**
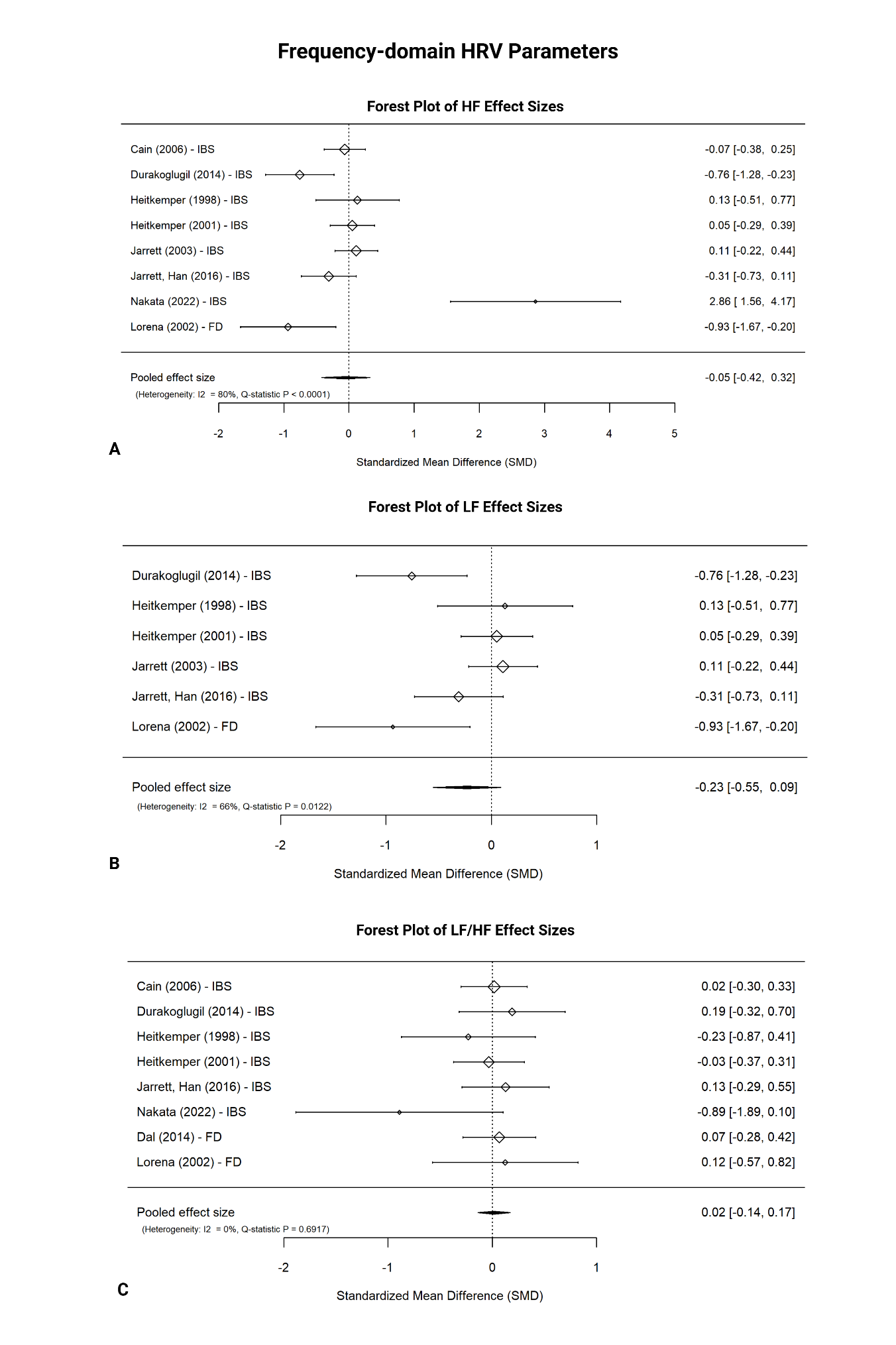
**

**Figure 10. Forest Plots of Frequency-Domain HRV Parameters.**

- 1. **Sleep**

Seven studies examined sleep in DGBI (n=1803), including IBS (n=146, 4 studies), FD (n=107, 1 study), and functional constipation (n= 1550, 2 studies), using wrist-mounted actigraphy (5 studies), Fitbit® (1 study), or Sleepthing™ (1 study). Reported outcomes included total sleep time ([TST], 6 studies), sleep efficiency ([SE], 5 studies), sleep latency ([SL], 4 studies), wake after sleep onset ([WASO], 5 studies), and the number of awakenings (2 studies). Five studies did not include healthy controls, limiting between-group comparisons.

Due to substantial heterogeneity among studies, only one exploratory meta-analysis could be conducted, limited to TST (3 studies: IBS n=2, functional constipation n =1), which showed no difference between DGBI and HCs (SMD=1.295, SE 1.175, 95% CI [- 1.008, 3.597]), with high heterogeneity (I^2^=95.90%, Q-statistic P<0.001) (Figure 12).

**Table 12: Studies Assessing Sleep as an Outcome Measure with Comparison between DGBIs and Healthy Controls.**

| **Outcome parameter** | **Studies reporting this parameter in IBS** | **Studies reporting this parameter in functional constipation** |
| --- | --- | --- |
| **TST** | Patel (2016), Rotem (2003). | Ono (2008). |
| **SE** | Rotem (2003). | - |
| **SL** | Rotem (2003). | - |
| **WASO** | Rotem (2003). | Ono (2008). |
| **AwI** | Rotem (2003). | - |

AwI, awakening index; DGBI, Disorder or Gut-Brain Interaction; HC, Healthy Controls; IBS, Irritable Bowel Syndrome; SE, sleep efficiency; SFI, sleep fragmentation index; SL, sleep latency; TST, total sleep time; WASO, wake after sleep onset.

**TST**

**Table 13: Sensitivity Analyses for TST**

| **Study** | **estimate** | **se** | **zval** | **pval** | **ci.lb** | **ci.ub** | **Q** | **Qp** | **Tau2** | **I2** | **H2** |
| --- | --- | --- | --- | --- | --- | --- | --- | --- | --- | --- | --- |
| **Patel (2016)** | 0.0196 | 0.2633 | 0.0745 | 0.9406 | -0.4964 | 0.5357 | 0.3876 | 0.5335 | 0.0000 | 0.0000 | 1.0000 |
| **Rotem (2003)** | 2.0113 | 1.7683 | 1.1374 | 0.2554 | -1.4546 | 5.4771 | 29.4497 | 0.0000 | 6.0417 | 96.6044 | 29.4497 |
| **Ono (2008)** | 1.8266 | 1.9408 | 0.9412 | 0.3466 | -1.9773 | 5.6306 | 45.8136 | 0.0000 | 7.3696 | 97.8172 | 45.8136 |

This table presents the results of the leave-one-out sensitivity analysis for Total Sleep Time (TST). The columns show the study name, effect estimate, standard error (se), z-value (zval), p-value (pval), confidence interval (CI) lower bound (ci.lb) and upper bound (ci.ub), Q-statistic, p-value for Q-statistic (Qp), Tau^2^, I^2^ (percentage of variation across studies), and H^2^ (heterogeneity index).

**
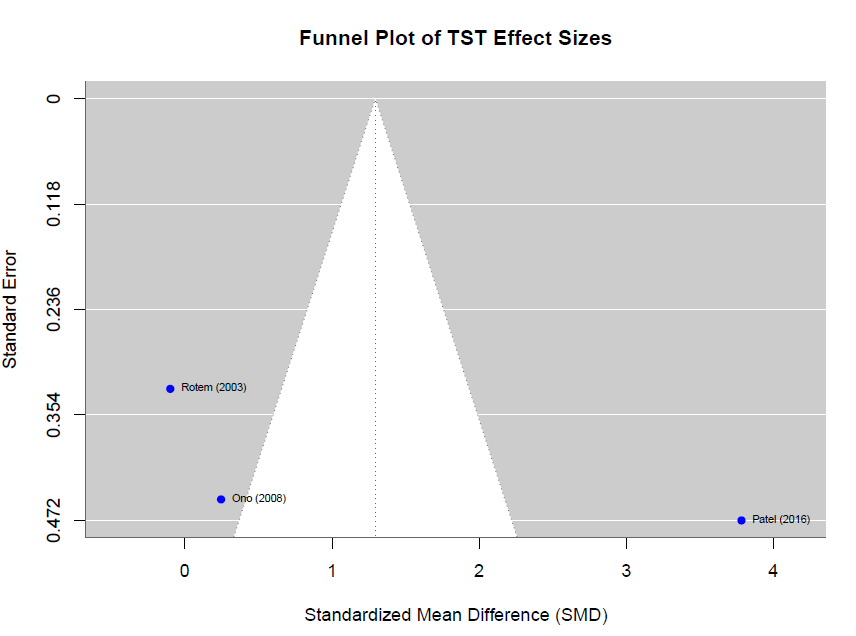
**

**Figure 11. Funnel Plot of TST Effect Sizes**

**
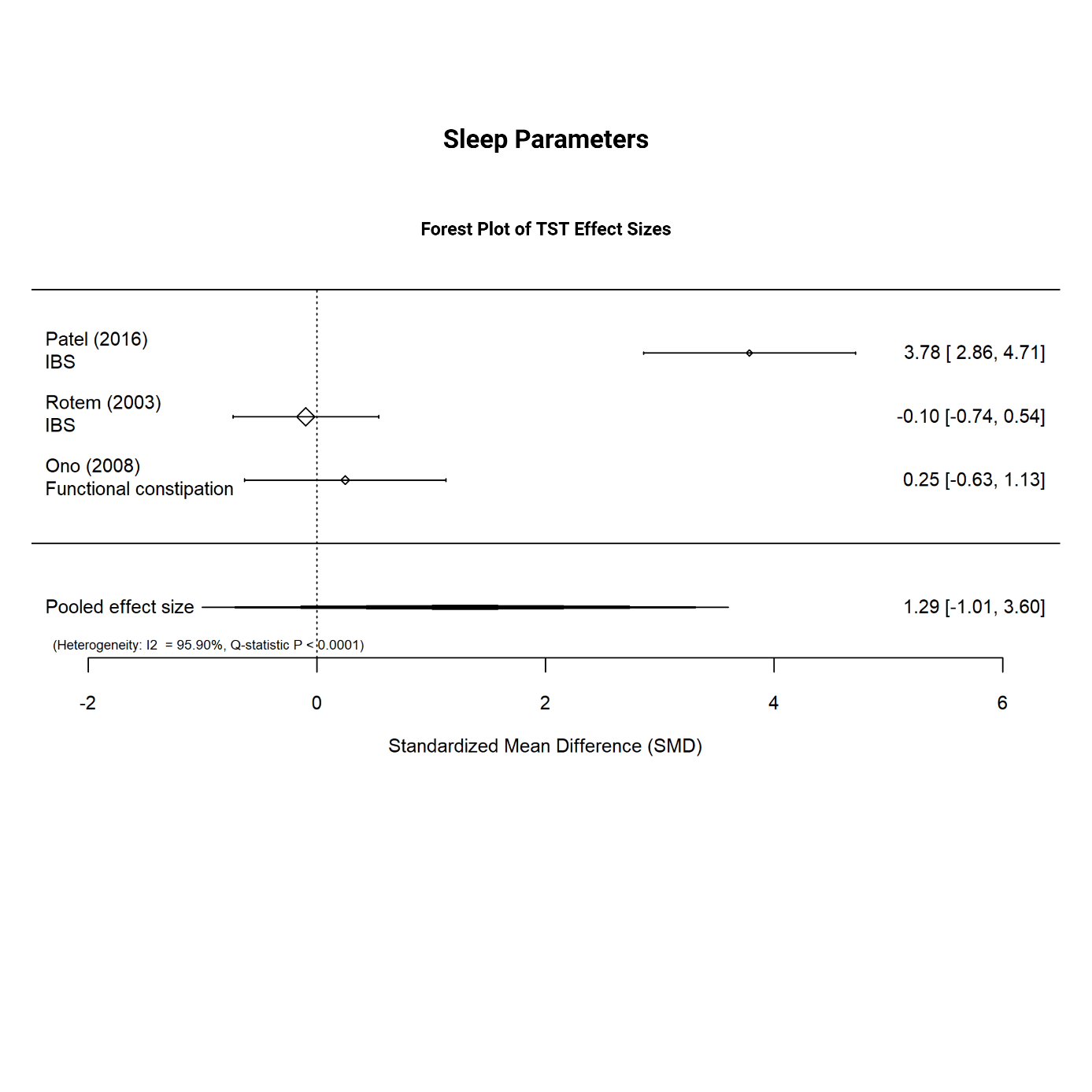
Figure 12. Forest Plots of Sleep Parameters.**

- 1. **Gastric Myoelectric Activity**

Fourteen studies assessed GI function using ambulatory electrogastrography (n=13, including 4 with high-resolution BSGM) or wireless motility patches (n=1) (Table 2). The DGBIs studied (n=1078) included IBS (n=10, 1 study), FD (n= 845, 7 studies), functional vomiting (n=19, 1 study), chronic nausea and vomiting syndrome ([CNVS], n= 93, 2 studies), and chronic gastroduodenal symptoms (n=111, 3 studies). Reported outcomes included dominant frequency, dominant power, percentage of normal gastric slow waves, bradygastria, tachygastria, and power ratio. Four studies lacked comparisons with HCs. The wearable devices employed distinct technical approaches, contributing to heterogeneity and limiting direct comparisons.

Six studies were included in the meta-analyses on gastric myoelectric activity. Two studies (42, 43) had a serious risk of bias but were retained for broader perspective. No sensitivity analyses were performed due to insufficient remaining studies, and all analyses were exploratory.

Dominant frequency (DF, 4 studies: FD n=1, functional vomiting n=1, CNVS n=2) did not show consistent differences between DGBIs and HCs (SMD= –0.089, SE=0.323, 95%CI [-0.722, 0.544], I^2^=85.62%, Q-statistic P<0.001) (Figure 17A)). The percentage of normal gastric slow waves (4 studies: FD n = 3, functional vomiting n = 1) was lower in DGBIs (SMD=-0.722 (SE=0.216, 95%CI [-1.146, -0.298], I^2^=34.78%, Q-statistic P=0.204) (Figure 17B). No differences were observed in bradygastria or tachygastria (3 studies: FD n = 2, functional vomiting n = 1) between DGBIs and HCs (bradygastria: SMD=0.326, SE=0.189, 95%CI [-0.043, 0.696], I^2^=0.00%, Q-statistic P=0.436; tachygastria: SMD=0.234. SE=0.437, 95%CI [-0.623, 1.091], I^2^=78.53%, Q-statistic P=0.010) (Figure 17C, 17D).

**Table 14: Studies Assessing Gastric Myoelectric Activity as an Outcome Measure with Comparison between DGBIs and Healthy Controls.**

| **Outcome parameter** | **Studies reporting this parameter in IBS** | **Studies reporting this parameter in FD** | **Studies reporting this parameter in functional vomiting** | **Studies reporting this parameter in CNVS** | **Studies reporting this parameter in Chronic gastroduodenal symptoms** |
| --- | --- | --- | --- | --- | --- |
| Dominant Frequency | - | Pfaffenbach (1997), Pfaffenbach (1998). | Zhao (2010). | Gharibans (2022), Schamberg (2023). | - |
| Dominant Power | - | Hocke (2001). | Zhao (2010). | - | - |
| Normal rhythm (%) | - | Hocke (2001), Pfaffenbach (1997), Pfaffenbach (1998), Miyaji (1999). | Zhao (2010). | - | - |
| Bradygastria (%) | - | Hocke (2001), Pfaffenbach (1997), Pfaffenbach (1998). | Zhao (2010). | - | - |
| Tachygastria (%) | - | Hocke (2001), Pfaffenbach (1997), Pfaffenbach (1998). | Zhao (2010). | - | - |
| Power Ratio | - | Pfaffenbach (1997), Pfaffenbach (1998). | - | Gharibans (2022). | - |

AUC, Area under the curve; BMI, body mass index; CNVS, Chronic Nausea and Vomiting Syndrome; DF, dominant frequency; DG, Dalitong Granule; DGBI, Disorder of Gut-Brain Interaction; EA, Electroacupuncture; FD, Functional Dyspepsia; GA-R, Gastric Alimetry Rhythm; DFIC, Dominant frequency instability coefficient; GE, Gastric Emptying; HC, Healthy Controls; IBS, Irritable Bowel Syndrome; PR, power ratio; TIDM, Type I Diabetes Mellitus; TIIDM, Type II Diabetes Mellitus.

**DF**

**Table 15: Sensitivity Analysis for DF**

| **Study** | **estimate** | **se** | **zval** | **pval** | **ci.lb** | **ci.ub** | **Q** | **Qp** | **Tau2** | **I2** | **H2** |
| --- | --- | --- | --- | --- | --- | --- | --- | --- | --- | --- | --- |
| **Pfaffenbach (1998)** | -0.1361 | 0.4559 | -0.2986 | 0.7653 | -1.0298 | 0.7575 | 20.6975 | 0.0000 | 0.5490 | 90.3370 | 10.3487 |
| **Zhao (2010)** | 0.0680 | 0.3568 | 0.1906 | 0.8488 | -0.6313 | 0.7673 | 16.6610 | 0.0002 | 0.3347 | 87.9959 | 8.3305 |
| **Gharibans (2022)** | 0.0537 | 0.3651 | 0.1472 | 0.8830 | -0.6618 | 0.7693 | 11.5089 | 0.0032 | 0.3214 | 82.6221 | 5.7544 |
| **Schamberg (2023)** | -0.3416 | 0.1932 | -1.7685 | 0.0770 | -0.7202 | 0.0370 | 2.9377 | 0.2302 | 0.0364 | 31.9194 | 1.4688 |

This table presents the results of the leave-one-out sensitivity analysis for dominant frequency (DF). The columns show the study name, effect estimate, standard error (se), z-value (zval), p-value (pval), confidence interval (CI) lower bound (ci.lb) and upper bound (ci.ub), Q-statistic, p-value for Q-statistic (Qp), Tau^2^, I^2^ (percentage of variation across studies), and H^2^ (heterogeneity index).

**
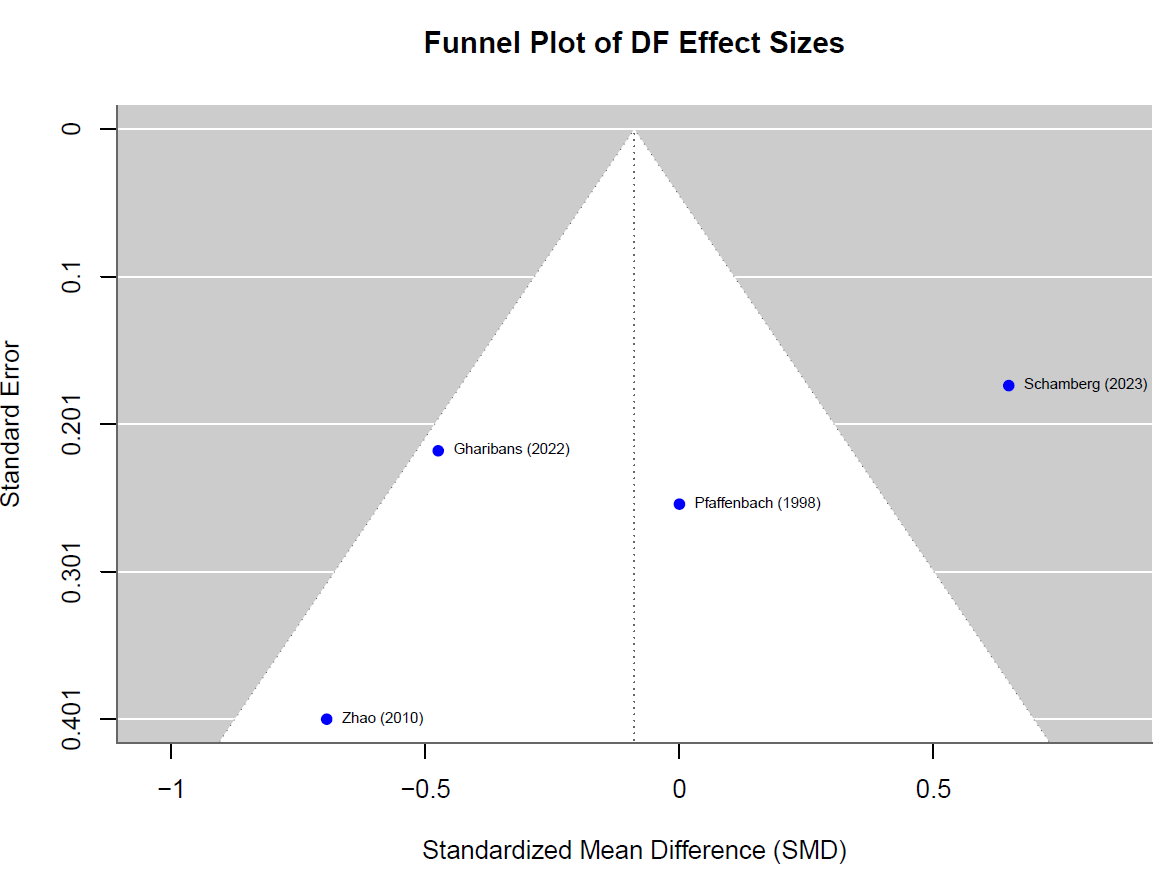
**

**Figure 13. Funnel Plot of DF Effect Sizes**

**Normal Rhythm (%)**

**Table 16: Sensitivity Analysis for Normal Rhythm (%)**

| **Study** | **estimate** | **se** | **zval** | **pval** | **ci.lb** | **ci.ub** | **Q** | **Qp** | **Tau2** | **I2** | **H2** |
| --- | --- | --- | --- | --- | --- | --- | --- | --- | --- | --- | --- |
| **Hocke (2001)** | -0.8898 | 0.1896 | -4.6929 | 0.0000 | -1.2614 | -0.5182 | 0.6811 | 0.7114 | 0.0000 | 0.0000 | 1.0000 |
| **Pfaffenbach (1998)** | -0.7076 | 0.3391 | -2.0865 | 0.0369 | -1.3722 | -0.0429 | 4.5791 | 0.1013 | 0.1942 | 56.3235 | 2.2896 |
| **Miyaji (1999)** | -0.6469 | 0.2899 | -2.2316 | 0.0256 | -1.2150 | -0.0787 | 4.0859 | 0.1296 | 0.1292 | 51.0517 | 2.0430 |
| **Zhao (2010)** | -0.6169 | 0.2489 | -2.4786 | 0.0132 | -1.1048 | -0.1291 | 3.3689 | 0.1855 | 0.0762 | 40.6341 | 1.6845 |

This table presents the results of the leave-one-out sensitivity analysis for the percentage of normal slow waves. The columns show the study name, effect estimate, standard error (se), z-value (zval), p-value (pval), confidence interval (CI) lower bound (ci.lb) and upper bound (ci.ub), Q-statistic, p-value for Q-statistic (Qp), Tau^2^, I^2^ (percentage of variation across studies), and H^2^ (heterogeneity index).

**
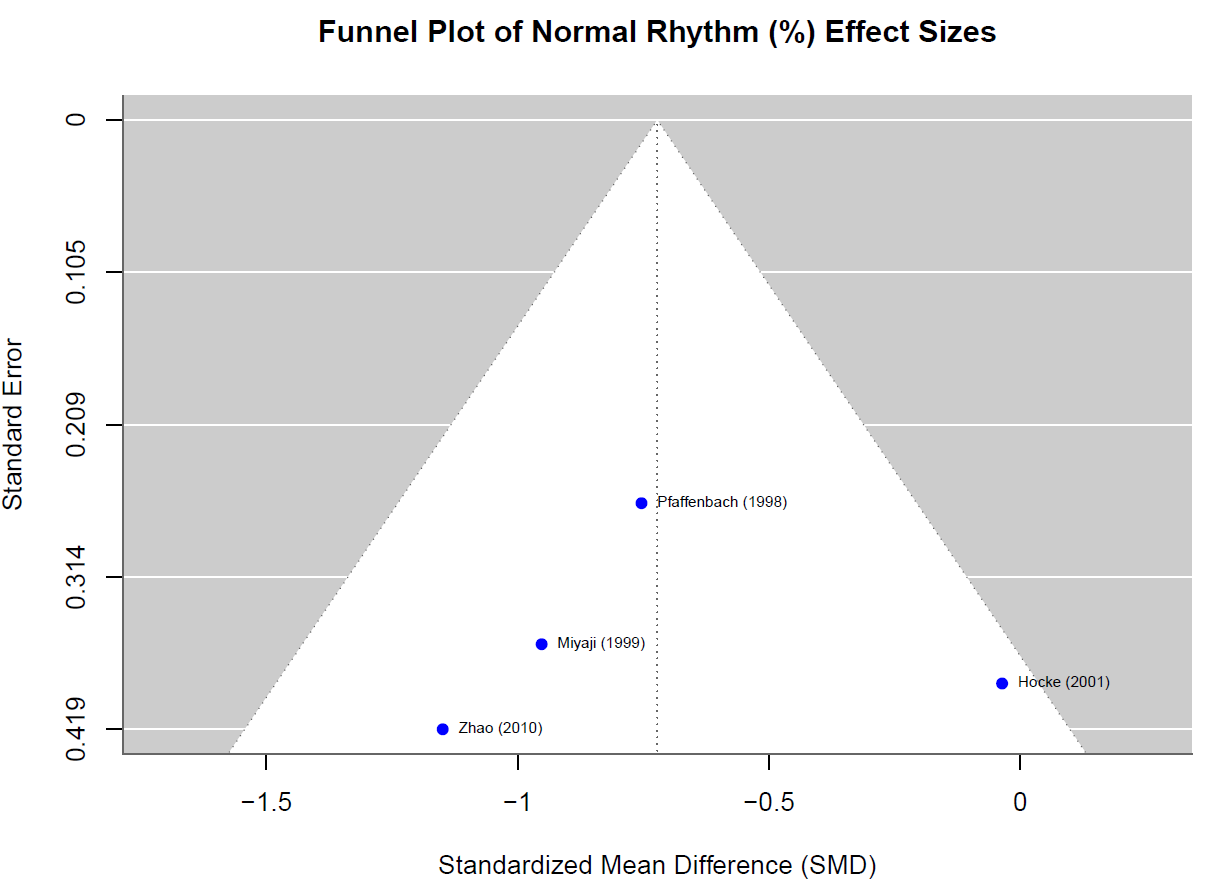
**

**Figure 14. Funnel Plot of Normal Rhythm (%) Effect Sizes**

**Bradygastria (%)**

**Table 17: Sensitivity Analysis for Bradygastria (%)**

| **Study** | **estimate** | **se** | **zval** | **pval** | **ci.lb** | **ci.ub** | **Q** | **Qp** | **Tau2** | **I2** | **H2** |
| --- | --- | --- | --- | --- | --- | --- | --- | --- | --- | --- | --- |
| **Hocke (2001)** | 0.2075 | 0.2142 | 0.9686 | 0.3328 | -0.2123 | 0.6273 | 0.2818 | 0.5955 | 0.0000 | 0.0000 | 1.0000 |
| **Pfaffenbach (1998)** | 0.5588 | 0.2803 | 1.9937 | 0.0462 | 0.0095 | 1.1081 | 0.4036 | 0.5252 | 0.0000 | 0.0000 | 1.0000 |
| **Zhao (2010)** | 0.3588 | 0.2925 | 1.2266 | 0.2200 | -0.2145 | 0.9322 | 1.6339 | 0.2012 | 0.0711 | 38.7976 | 1.6339 |

This table presents the results of the leave-one-out sensitivity analysis for bradygastria. The columns show the study name, effect estimate, standard error (se), z-value (zval), p-value (pval), confidence interval (CI) lower bound (ci.lb) and upper bound (ci.ub), Q-statistic, p-value for Q-statistic (Qp), Tau^2^, I^2^ (percentage of variation across studies), and H^2^ (heterogeneity index).

**
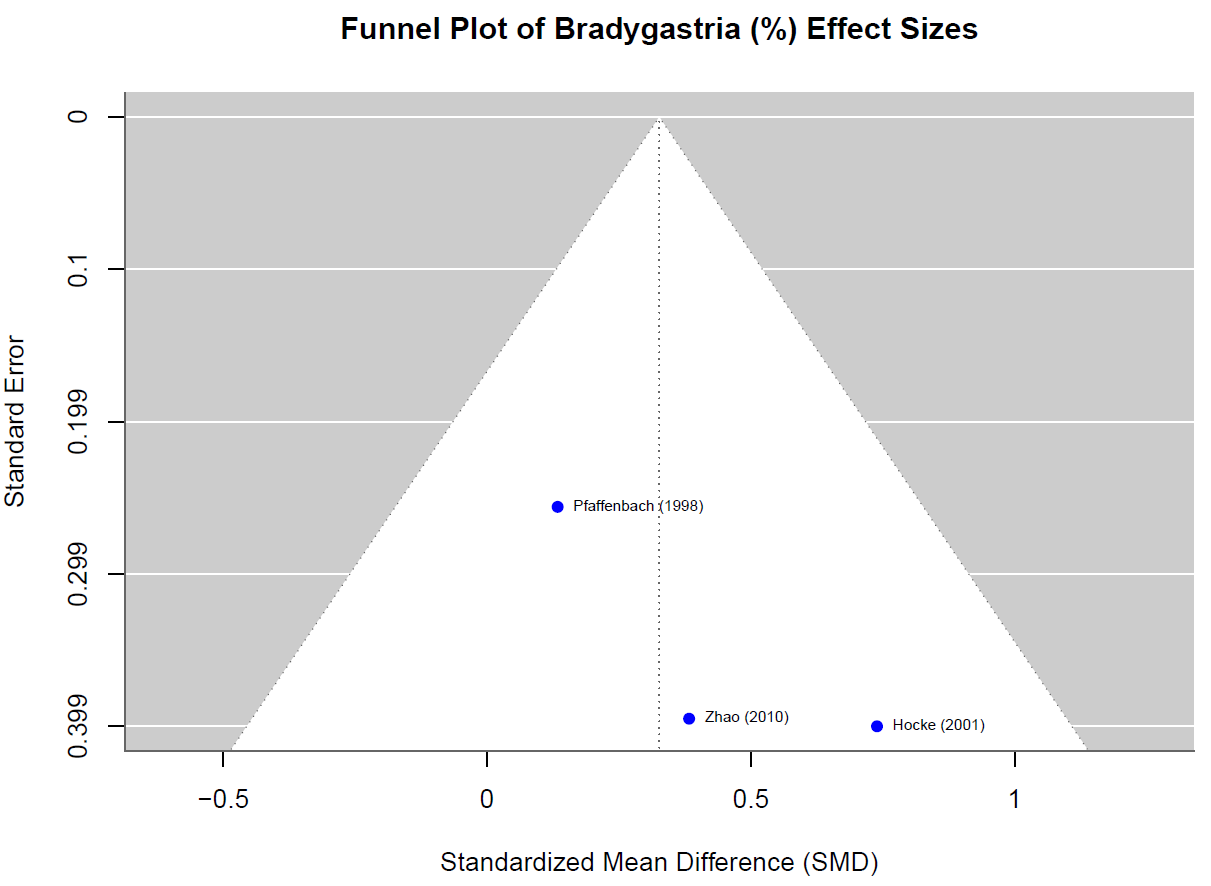
**

**Figure 15. Funnel Plot of Bradygastria (%) Effect Sizes**

**Tachygastria (%)**

**Table 18: Sensitivity Analysis for Tachygastria (%)**

| **Study** | **estimate** | **se** | **zval** | **pval** | **ci.lb** | **ci.ub** | **Q** | **Qp** | **Tau2** | **I2** | **H2** |
| --- | --- | --- | --- | --- | --- | --- | --- | --- | --- | --- | --- |
| **Hocke (2001)** | 0.6564 | 0.2188 | 2.9998 | 0.0027 | 0.2275 | 1.0854 | 0.0653 | 0.7983 | 0.0000 | 0.0000 | 1.0000 |
| **Pfaffenbach (1998)** | 0.0071 | 0.7346 | 0.0097 | 0.9923 | -1.4326 | 1.4469 | 6.7255 | 0.0095 | 0.9188 | 85.1312 | 6.7255 |
| **Zhao (2010)** | -0.0193 | 0.6724 | -0.0287 | 0.9771 | -1.3373 | 1.2986 | 7.9965 | 0.0047 | 0.7932 | 87.4945 | 7.9965 |

This table presents the results of the leave-one-out sensitivity analysis for tachygastria. The columns show the study name, effect estimate, standard error (se), z-value (zval), p-value (pval), confidence interval (CI) lower bound (ci.lb) and upper bound (ci.ub), Q-statistic, p-value for Q-statistic (Qp), Tau^2^, I^2^ (percentage of variation across studies), and H^2^ (heterogeneity index).

**
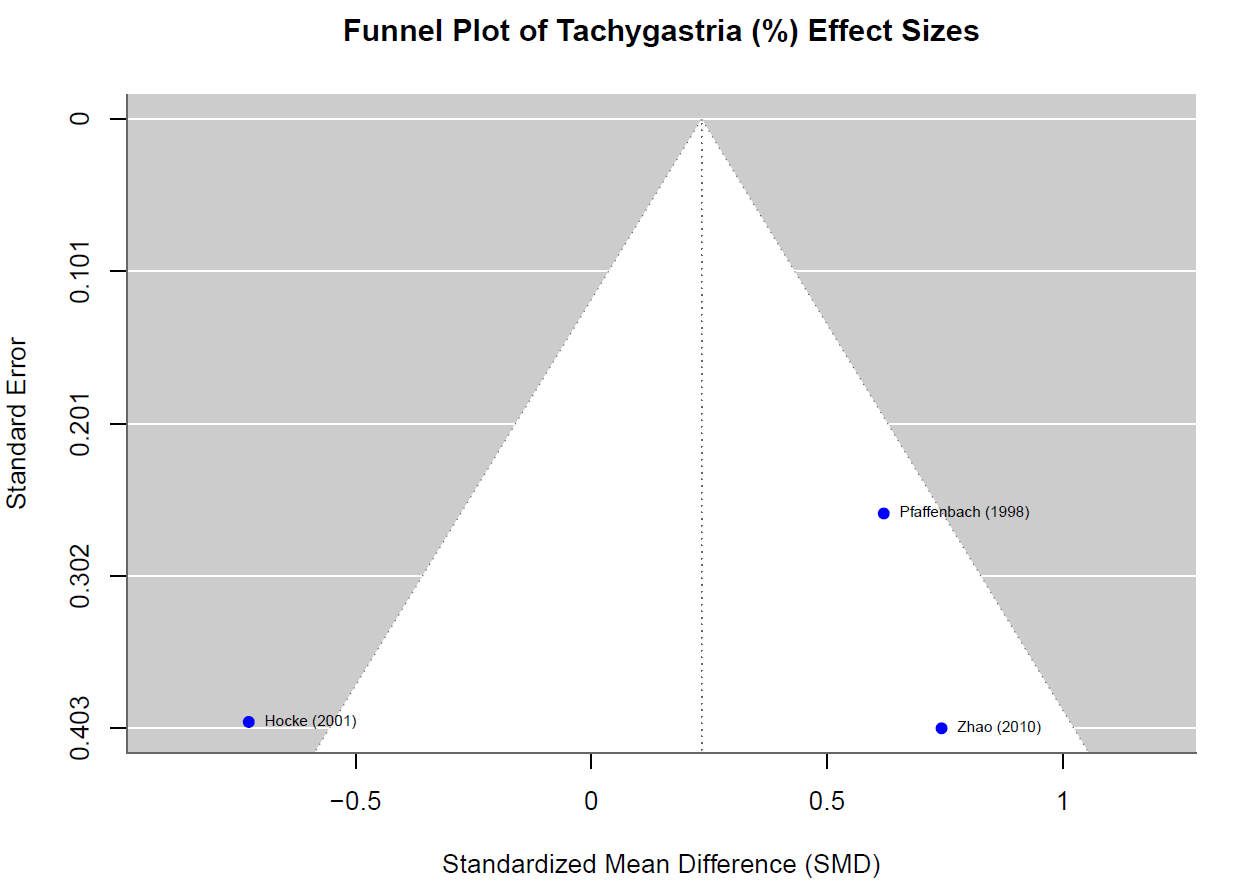
**

**Figure 16. Funnel Plot of Tachygastria (%) Effect Sizes**

**
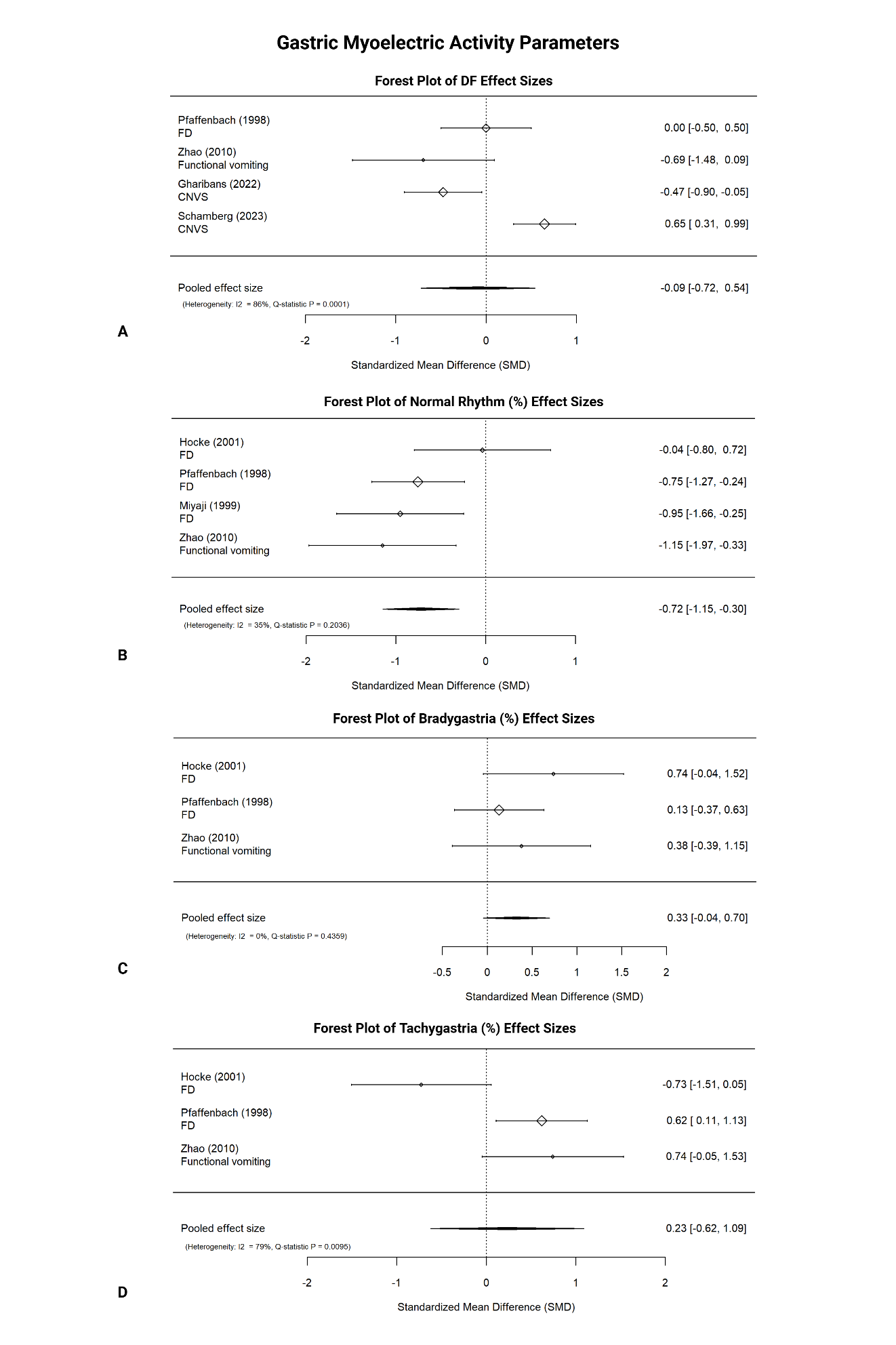
**

**Figure 17. Forest Plots of Gastric Myoelectric Activity Parameters**

- 1. **Relationship between Autonomic Function and GI Symptom Severity**

Twenty-three studies examined the relationship between autonomic function and GI symptoms: nine on HRV, six on sleep, and nine on gastric myoelectric activity, with one study assessing both HRV and sleep (45). HRV and symptom severity were studied in IBS (7 studies), FD (1 study) and functional constipation (1 study). Sleep was evaluated in IBS (4 studies), FD (1 study), and functional constipation (1 study). Gastric myoelectric activity and symptom severity were investigated in FD (4 studies), functional vomiting (1 study), CNVS (2 studies), and chronic gastroduodenal symptoms (2 studies).

No meta-analyses were performed due to heterogeneity with regards to symptom assessment applied in the different studies. In IBS, altered HRV profiles were associated with symptom severity and varied between subtypes, with reduced parasympathetic activity (e.g., lower RMSSD and pNN50) and increased sympathetic dominance (e.g., elevated LF/HF ratio) in more severe cases, particularly in constipation-predominant IBS (IBS-C) (11, 14, 16, 17, 25, 27). In FD (20) , no associations were found (based on one study), while in functional constipation (45), lower active heart rate correlated with irregular and constipated bowel movements (similarly only in a single study). Severe symptoms, particularly in IBS (19, 26) and functional constipation (45), were found to be linked to impaired sleep metrics (e.g., reduced TST and SE, and increased WASO) in a number of studies, though findings were inconsistent (15, 21). In FD (41), functional vomiting (39), CNVS (32, 44), and chronic gastroduodenal symptoms (40), associations between gastric myoelectric activity and symptom severity were noted, particularly linking abnormal EGG/BSGM patterns to more severe symptoms. However, findings varied across studies and symptom domains. See Table 2 (main document) for a summary of the main results.

**Table 19: Studies Assessing Autonomic Function in Relation to GI Symptom Severity**

| **Outcome parameter** | **Studies in IBS** | **Studies in FD** | **Studies in functional constipation** | **Studies in functional vomiting** | **Studies in CNVS** | **Studies in chronic gastroduodenal symptoms** |
| --- | --- | --- | --- | --- | --- | --- |
| **HRV** | Cain (2006), Chen (2024), Heitkemper (2001), Jarrett, Cain (2016), Jarrett, Han (2016), Polster (2018), Nakata (2022) | Tominaga (2016) | Shapiro (2021) | - | - | - |
| **Sleep** | Buchanan (2014), Patel (2016), Rotem (2003), Topan (2024) | Du (2023) | Shapiro (2021) | - | - | - |
| **Gastric myoelectric activity** | - | Riezzo (2001), Parkman (1997), Pfaffenbach (1997), Pfaffenbach (1998) | - | Zhao (2010) | Gharibans (2022), Schamberg (2023) | Lacy (2024), Wang (2023) |

CNVS, chronic nausea and vomiting syndrome; FD, functional dyspepsia; HRV, heart rate variability; IBS, irritable bowel syndrome.

**3.References**

1. Colomier E, Algera JP, Van den Houte K, Simrén M, Tack J. Mechanisms underlying food-related symptoms in disorders of gut-brain interaction: Course ahead in research and clinical practice. Best Pract Res Clin Gastroenterol. 2023;62-63:101824.

2. Sterne JAC, Savović J, Page MJ, Elbers RG, Blencowe NS, Boutron I, et al. RoB 2: a revised tool for assessing risk of bias in randomised trials. Bmj. 2019;366:l4898.

3. Sterne JA, Hernán MA, Reeves BC, Savović J, Berkman ND, Viswanathan M, et al. ROBINS-I: a tool for assessing risk of bias in non-randomised studies of interventions. Bmj. 2016;355:i4919.

4. Ali MK, Chen JDZ. Roles of Heart Rate Variability in Assessing Autonomic Nervous System in Functional Gastrointestinal Disorders: A Systematic Review. Diagnostics (Basel). 2023;13(2).

5. Shaffer F, Ginsberg JP. An Overview of Heart Rate Variability Metrics and Norms. Front Public Health. 2017;5:258.

6. Shaffer F, McCraty R, Zerr CL. A healthy heart is not a metronome: an integrative review of the heart's anatomy and heart rate variability. Front Psychol. 2014;5:1040.

7. Robbins R, Seixas A, Masters LW, Chanko N, Diaby F, Vieira D, Jean-Louis G. Sleep tracking: A systematic review of the research using commercially available technology. Curr Sleep Med Rep. 2019;5(3):156-63.

8. Cudney LE, Frey BN, McCabe RE, Green SM. Investigating the relationship between objective measures of sleep and self-report sleep quality in healthy adults: a review. J Clin Sleep Med. 2022;18(3):927-36.

9. Chen JD, Co E, Liang J, Pan J, Sutphen J, Torres-Pinedo RB, Orr WC. Patterns of gastric myoelectrical activity in human subjects of different ages. Am J Physiol. 1997;272(5 Pt 1):G1022-7.

10. Boucsein W, Fowles DC, Grimnes S, Ben-Shakhar G, roth WT, Dawson ME, Filion DL. Publication recommendations for electrodermal measurements. Psychophysiology. 2012;49(8):1017-34.

11. Jarrett ME, Cain KC, Barney PG, Burr RL, Naliboff BD, Shulman R, et al. Balance of Autonomic Nervous System Predicts Who Benefits from a Self-management Intervention Program for Irritable Bowel Syndrome. J Neurogastroenterol Motil. 2016;22(1):102-11.

12. Du H, Lin R, Xiao S, Zhao Y, Wu M, Chen W, et al. Improved Sleep Affects Epigastric Pain in Functional Dyspepsia by Reducing the Levels of Inflammatory Mediators. Dig Dis. 2023;41(6):835-44.

13. Zhang CX, Guo LK. Dalitong granule combined with electroacupuncture in the treatment of functional dyspepsia: A randomized controlled trial. Chin J Integr Med. 2015;21(10):743-50.

14. Chen LJ, Burr R, Cain K, Kamp K, Heitkemper M. Age Differences in Upper Gastrointestinal Symptoms and Vagal Modulation in Women With Irritable Bowel Syndrome. Biol Res Nurs. 2024;26(1):46-55.

15. Topan R, Vork L, Fitzke H, Pandya S, Keszthelyi D, Cornelis J, et al. Poor Subjective Sleep Quality Predicts Symptoms in Irritable Bowel Syndrome Using the Experience Sampling Method. Am J Gastroenterol. 2024;119(1):155-64.

16. Nakata R, Tanaka F, Sugawara N, Kojima Y, Takeuchi T, Shiba M, et al. Analysis of autonomic function during natural defecation in patients with irritable bowel syndrome using real-time recording with a wearable device. PLoS One. 2022;17(12):e0278922.

17. Polster A, Friberg P, Gunterberg V, Öhman L, Le Nevé B, Törnblom H, et al. Heart rate variability characteristics of patients with irritable bowel syndrome and associations with symptoms. Neurogastroenterol Motil. 2018;30(7):e13320.

18. Jarrett ME, Han CJ, Cain KC, Burr RL, Shulman RJ, Barney PG, et al. Relationships of abdominal pain, reports to visceral and temperature pain sensitivity, conditioned pain modulation, and heart rate variability in irritable bowel syndrome. Neurogastroenterol Motil. 2016;28(7):1094-103.

19. Patel A, Hasak S, Cassell B, Ciorba MA, Vivio EE, Kumar M, et al. Effects of disturbed sleep on gastrointestinal and somatic pain symptoms in irritable bowel syndrome. Aliment Pharmacol Ther. 2016;44(3):246-58.

20. Tominaga K, Fujikawa Y, Tsumoto C, Kadouchi K, Tanaka F, Kamata N, et al. Disorder of autonomic nervous system and its vulnerability to external stimulation in functional dyspepsia. J Clin Biochem Nutr. 2016;58(2):161-5.

21. Buchanan DT, Cain K, Heitkemper M, Burr R, Vitiello MV, Zia J, Jarrett M. Sleep measures predict next-day symptoms in women with irritable bowel syndrome. J Clin Sleep Med. 2014;10(9):1003-9.

22. Dal K, Deveci OS, Kucukazman M, Ata N, Sen O, Ozkan S, et al. Decreased parasympathetic activity in patients with functional dyspepsia. Eur J Gastroenterol Hepatol. 2014;26(7):748-52.

23. Pfaffenbach B, Adamek RJ, Lux G. [The value of electrogastrography for the diagnosis of gastroenterologic function]. Dtsch Med Wochenschr. 1998;123(28-29):855-60.

24. Jarrett ME, Burr RL, Cain KC, Hertig V, Weisman P, Heitkemper MM. Anxiety and depression are related to autonomic nervous system function in women with irritable bowel syndrome. Dig Dis Sci. 2003;48(2):386-94.

25. Cain KC, Jarrett ME, Burr RL, Hertig VL, Heitkemper MM. Heart rate variability is related to pain severity and predominant bowel pattern in women with irritable bowel syndrome. Neurogastroenterol Motil. 2007;19(2):110-8.

26. Rotem AY, Sperber AD, Krugliak P, Freidman B, Tal A, Tarasiuk A. Polysomnographic and actigraphic evidence of sleep fragmentation in patients with irritable bowel syndrome. Sleep. 2003;26(6):747-52.

27. Heitkemper M, Jarrett M, Cain KC, Burr R, Levy RL, Feld A, Hertig V. Autonomic nervous system function in women with irritable bowel syndrome. Dig Dis Sci. 2001;46(6):1276-84.

28. Heitkemper M, Burr RL, Jarrett M, Hertig V, Lustyk MK, Bond EF. Evidence for autonomic nervous system imbalance in women with irritable bowel syndrome. Dig Dis Sci. 1998;43(9):2093-8.

29. Orr WC, Crowell MD, Lin B, Harnish MJ, Chen JD. Sleep and gastric function in irritable bowel syndrome: derailing the brain-gut axis. Gut. 1997;41(3):390-3.

30. Law M, Schamberg G, Gharibans A, Sebaratnam G, Foong D, Varghese C, et al. Short- and long-term reproducibility of body surface gastric mapping using the Gastric Alimetry® system. Neurogastroenterol Motil. 2024;36(7):e14812.

31. Pfaffenbach B, Adamek RJ, Bartholomäus C, Wegener M. Gastric dysrhythmias and delayed gastric emptying in patients with functional dyspepsia. Dig Dis Sci. 1997;42(10):2094-9.

32. Gharibans AA, Calder S, Varghese C, Waite S, Schamberg G, Daker C, et al. Gastric dysfunction in patients with chronic nausea and vomiting syndromes defined by a noninvasive gastric mapping device. Sci Transl Med. 2022;14(663):eabq3544.

33. Wang WJ, Foong D, Calder S, Schamberg G, Varghese C, Tack J, et al. Gastric Alimetry Expands Patient Phenotyping in Gastroduodenal Disorders Compared with Gastric Emptying Scintigraphy. Am J Gastroenterol. 2024;119(2):331-41.

34. Durakoğlugil ME, Canga A, Kocaman SA, Akdoğan RA, Durakoğlugil T, Ergül E, et al. The effect of irritable bowel syndrome on carotid intima-media thickness, pulse wave velocity, and heart rate variability. Anadolu Kardiyol Derg. 2014;14(6):525-30.

35. Ochi M, Tominaga K, Tanaka F, Tanigawa T, Yamagami H, Watanabe K, et al. Clinical classification of subgroups according to the Rome III criteria cannot be used to distinguish the associated respective pathophysiology in Japanese patients with functional dyspepsia. Intern Med. 2013;52(12):1289-93.

36. Ono S, Komada Y, Kamiya T, Shirakawa S. A pilot study of the relationship between bowel habits and sleep health by actigraphy measurement and fecal flora analysis. J Physiol Anthropol. 2008;27(3):145-51.

37. Lorena SL, Figueiredo MJ, Almeida JR, Mesquita MA. Autonomic function in patients with functional dyspepsia assessed by 24-hour heart rate variability. Dig Dis Sci. 2002;47(1):27-31.

38. Riezzo G, Chiloiro M, Russo F, Clemente C, Di Matteo G, Guerra V, Di Leo A. Gastric electrical activity and gastrointestinal hormones in dyspeptic patients. Digestion. 2001;63(1):20-9.

39. Zhao Y, Ke M, Wang Z, Wei J, Zhu L, Sun X, Zhang J. Pathophysiological and psychosocial study in patients with functional vomiting. J Neurogastroenterol Motil. 2010;16(3):274-80.

40. Lacy BE, Cangemi DJ, Accurso JM, Axelrod S, Axelrod L, Navalgund A. A novel pilot study to evaluate the efficacy and safety of a wireless patch system in patients with chronic nausea and vomiting. Neurogastroenterol Motil. 2024;36(9):e14862.

41. Parkman HP, Miller MA, Trate D, Knight LC, Urbain JL, Maurer AH, Fisher RS. Electrogastrography and gastric emptying scintigraphy are complementary for assessment of dyspepsia. J Clin Gastroenterol. 1997;24(4):214-9.

42. Miyaji H, Azuma T, Ito S, Abe Y, Ono H, Suto H, et al. The effect of helicobacter pylori eradication therapy on gastric antral myoelectrical activity and gastric emptying in patients with non-ulcer dyspepsia. Aliment Pharmacol Ther. 1999;13(11):1473-80.

43. Hocke M, Seidel T, Sprott H, Oelzner P, Eitner K, Bosseckert H. Ambulatory electrogastrography in patients with sclerodermia, delayed gastric emptying, dyspepsia, and irritable bowel syndrome. Is there any clinical relevance? Eur J Intern Med. 2001;12(4):366-71.

44. Schamberg G, Calder S, Varghese C, Xu W, Wang WJ, Ho V, et al. Comparison of Gastric Alimetry(®) body surface gastric mapping versus electrogastrography spectral analysis. Sci Rep. 2023;13(1):14987.

45. Shapiro A, Bradshaw B, Landes S, Kammann P, Bois De Fer B, Lee WN, Lange R. A novel digital approach to describe real world outcomes among patients with constipation. NPJ Digit Med. 2021;4(1):27.
